# A structural model for (GlcNAc)_2_ translocation *via* a periplasmic chitooligosaccharide-binding protein from marine *Vibrio* bacteria

**DOI:** 10.1016/j.jbc.2021.101071

**Published:** 2021-08-13

**Authors:** Yoshihito Kitaoku, Tamo Fukamizo, Sawitree Kumsaoad, Prakayfun Ubonbal, Robert C. Robinson, Wipa Suginta

**Affiliations:** 1 School of Biomolecular Science and Engineering (BSE), Vidyasirimedhi Institute of Science and Technology (VISTEC), Rayong, Thailand; 2 Research Institute of Interdisciplinary Science (RIIS), Okayama University, Okayama, Japan

**Keywords:** chitin metabolism, chitin-binding protein, isothermal microcalorimetry, marine bacteria, periplasmic solute-binding proteins, sugar translocation, *Vibrio* spp., X-ray crystallography, CBP, chitooligosaccharide-binding protein, ChiP, chitoporin, ITC, isothermal titration calorimetry, SBP, solute-binding protein, *Tm*CeBP, cellooligosaccharide-specific SBP from *Thermotoga maritima*, *Tm*MnBP, mannooligosaccharide-specific SBP from *T. maritima*, *Vc*CBP, CBP from *Vibrio cholerae*, *Vh*CBP, CBP from *Vibriocampbellii* (formerly *V. harveyi*) type strain ATCC BAA-1116

## Abstract

*Vh*CBP is a periplasmic chitooligosaccharide-binding protein mainly responsible for translocation of the chitooligosaccharide (GlcNAc)_2_ across the double membranes of marine bacteria. However, structural and thermodynamic understanding of the sugar-binding/-release processes of *Vh*CBP is relatively less. *Vh*CBP displayed the greatest affinity toward (GlcNAc)_2_, with lower affinity for longer-chain chitooligosaccharides [(GlcNAc)_3–4_]. (GlcNAc)_4_ partially occupied the closed sugar-binding groove, with two reducing-end GlcNAc units extending beyond the sugar-binding groove and barely characterized by weak electron density. Mutation of three conserved residues (Trp^363^, Asp^365^, and Trp^513^) to Ala resulted in drastic decreases in the binding affinity toward the preferred substrate (GlcNAc)_2_, indicating their significant contributions to sugar binding. The structure of the W513A–(GlcNAc)_2_ complex in a ‘half-open’ conformation unveiled the intermediary step of the (GlcNAc)_2_ translocation from the soluble CBP in the periplasm to the inner membrane–transporting components. Isothermal calorimetry data suggested that *Vh*CBP adopts the high-affinity conformation to bind (GlcNAc)_2_, while its low-affinity conformation facilitated sugar release. Thus, chitooligosaccharide translocation, conferred by periplasmic *Vh*CBP, is a crucial step in the chitin catabolic pathway, allowing *Vibrio* bacteria to thrive in oceans where chitin is their major source of nutrients.

*Vibrio* spp. are Gram-negative, ubiquitous bacteria that live in oceanic, estuarine, and fresh waters. Several *Vibrio* species are serious pathogens of humans, aquatic animals, and invertebrates, including crustaceans and zooplankton (1). In marine ecosystems, *Vibrio* spp. are the major consumers of chitin, the most abundant polysaccharide, and thus control the carbon and nitrogen balance on earth (2). Studies on chitin assimilation by *Vibrio* spp. revealed the expression profile of genes involved in chitin utilization and chitin-signal transduction (3, 4, 5). Chitin, a β-1,4-linked polysaccharide of GlcNAc, is hydrolyzed by various types of chitinases (6), β-*N*-acetyl-glucosaminidase (7, 8) and chitin deacetylases (9). *Vibrio* spp. possess all of these enzymes, efficiently catabolizing chitin as their nutritional source. The enzymatic degradation of chitin produces chitooligosaccharides (GlcNAc)_n_ (n = 2, 3, 4, 5, and 6), which are transported into the periplasmic space through an outer membrane porin, namely chitoporin (ChiP) (10, 11, 12) The transported (GlcNAc)_n_ are further recognized by a periplasmic solute-binding protein (SBP) specific for (GlcNAc)_n_, namely chitooligosaccharide-binding protein (CBP) (13). CBP itself is a biological component of the chitooligosaccharide-specific ABC transporter localized to the inner membrane (5).

According to the classification proposed by Scheepers *et al.* (14), SBPs are structurally classified in seven clusters (A, B, C, D, E, F, and G), and cluster C is subdivided into five subclusters (I, II, III, IV, and V) (15). SBPs share a common structural feature comprising two domains connected by a flexible hinge region (16). The flexibility of the hinge enables them to admit specific solutes into the substrate-binding cavity between the two domains. The chitooligosaccharide-specific SBP is localized to the periplasmic compartment of the bacterial cell wall and serves as a mobile component of the (GlcNAc)_2_-specific ABC transporter (5, 11). Although the first CBP has been identified in *Vibrio cholerae* (*Vc*CBP), no functional/mechanistic details of *Vc*CBP were reported (5). Recently, Suginta *et al.* (13) reported the crystal structure of CBP from *Vibrio campbellii* (formerly *V. harveyi*) type strain ATCC BAA-1116 (*Vh*CBP) and the functional characterization indicated that *Vh*CBP strongly binds (GlcNAc)_2,3,4_. *Vc*CBP and *Vh*CBP belong to cluster C/subcluster IV (C-IV), members of which are specific for oligosaccharides, including mannooligosaccharides, cellooligosaccharides, and chitooligosaccharides (5).

Chitooligosaccharide binding to *Vibrio* CBPs has been proposed to trigger not only translocation of the bound chitooligosaccharides through the inner membrane by the (GlcNAc)_2_-specific ABC transporter but also activation of the gene cluster involved in the chitinolytic cascade, releasing CBPs from chitin-sensor kinase, ChiS, localized to the inner membrane (17). Both biological processes have been shown to require chitooligosaccharide-induced conformational changes by the CBPs. Although the structure and mechanism of a ChiP from *Vibrio campbellii* (formerly *V. harveyi*) type strain ATCC BAA-1116 were intensively studied by crystallography in combination with physicochemical and electrochemical techniques (10, 12, 18, 19, 20), the molecular mechanism of the periplasmic CBP component involved in sugar translocation and control of gene activation is unknown. In this study, we have focused on the molecular basis of chitooligosaccharide binding and translocation by CBP, a periplasmic SBP specific for chitooligosaccharides. The specific interactions of *Vh*CBP with chitooligosaccharides (GlcNAc)_2–4_ were analyzed from the crystal structures. Furthermore, conserved amino acids identified as sugar-binding residues were mutated, and the mutant proteins were characterized with respect to their (GlcNAc)_2_-binding properties. Thermodynamic data obtained from isothermal titration calorimetry (ITC) binding experiments are discussed in the light of crystal structures of the individual *Vh*CBP mutants. Based on these findings, we here propose a ‘ping-pong’ model for binding/release during (GlcNAc)_2_ translocation within the periplasmic space.

## Results

### Mutational design, recombinant expression, and purification

**Figure 1:**
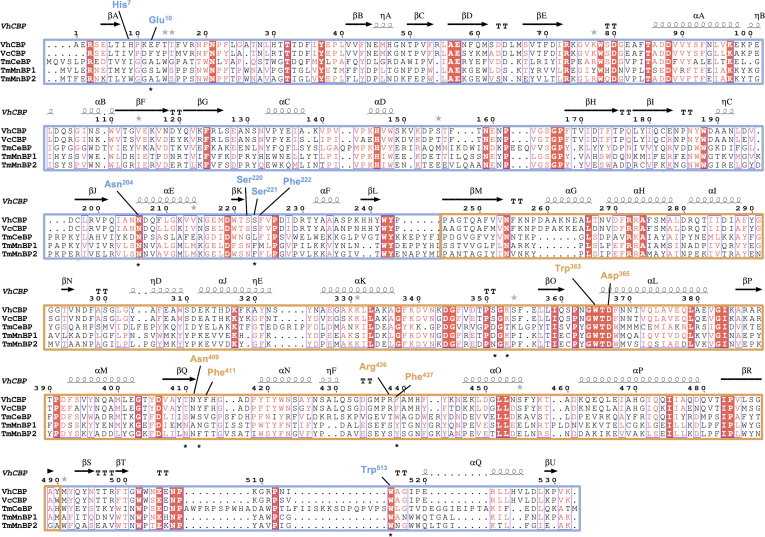
**Structure-based sequence alignment of the sugar-specific SBPs belonging to cluster C/subcluster IV.** The alignment was performed by MUSCLE and depicted with ESPript *v*.3.0. The sequences corresponding to the *upper domain* are *boxed* by the *lines* colored in *marine* and the *lower domain* in *orange*. Mutation sites are labeled with *asterisks*, and amino acid notations with the sequence number are colored in *blue* for the *upper domain* and in *orange* the for the *lower domain*. The secondary structural elements of *Vh*CBP (5YQW) are also aligned with the amino acid sequence of *Vh*CBP. SBPs, solute-binding proteins; *Vh*CBP, CBP from *V. campbellii* type strain ATCC BAA-1116.

To identify important amino acid residues in SBPs belonging to the cluster C/subcluster IV, we carried out multiple sequence alignments of five orthologs, including *Vh*CBP, *Vc*CBP, cellooligosaccharide-specific SBP from *Thermotoga maritima* (*Tm*CeBP), and mannooligosaccharide-specific SBPs from *T. maritima* (*Tm*MnBP1 and *Tm*MnBP2) (Fig. 1). Based on the crystal structure of *Vh*CBP in complex with (GlcNAc)_2_ (13), Glu^10^, Asn^204^, Ser^221^, Trp^363^, Asp^365^, Asn^409,^ Phe^411^, Arg^436^, Phe^437^, and Trp^513^ were found to interact with (GlcNAc)_2_ and were therefore selected as targets for mutation. Four of these residues, Asn^204^, Trp^363^, Asp^365^, and Trp^513^, are completely conserved, and the others are partially conserved. *Vh*CBP and the individual mutants were successfully expressed and purified, and all purified *Vh*CBPs migrated as single protein bands on SDS-PAGE. The yields of the individual proteins from 1 l of the culture medium were 20 to 30 mg for WT and 9 to 15 mg for the mutants. We failed to produce R436A in quantities sufficient for further crystallographic and ITC binding experiments. Arg^436^ may be involved in the correct folding of the protein because protein was mostly present in the ‘inclusion bodies.’

### Evaluation of binding affinity of WT *Vh*CBP to (GlcNAc)_2–4_

We previously reported ITC data for (GlcNAc)_2–4_ binding to *Vh*CBP in 20 mM potassium phosphate, pH 8.0, at 4 °C (13). Here, we re-evaluated the ITC data by conducting binding experiments in Tris HCl, pH 8.0, at 25 °C using (GlcNAc)_n_ (n = 2–4) as the ligands. The measurements are summarized in Table 1. The binding interactions for individual ligands were determined assuming a 1:1 stoichiometry. Favorable enthalpy changes (−3.9 to −4.8 kcal mol^−1^) and entropy changes (−6.4 to −4.0 kcal mol^−1^) contributed similarly to the interactions. From these data, we obtained the Δ*G*° values and the dissociation constants (*K*_d_) for (GlcNAc)_2–4_: the binding affinities were fairly dependent on the degree of polymerization of the ligands, with (GlcNAc)_3_ exhibiting 3-fold and (GlcNAc)_4_ 12-fold lower affinity than (GlcNAc)_2_.

**Table 1:** Thermodynamic parameters of *Vh*CBP titrated with (GlcNAc)_2–4_

Ligand	n	*K*_d_ (μM)	Δ*H*^*ο*^ (kcal.mol^−1^)	−*T*Δ*S*^*ο*^ (kcal.mol^−1^)	Δ*G*^*ο*^ (kcal.mol^−1^)
(GlcNAc)_2_	1	0.03 ± 0.01	−3.9 ± 0.1	−6.4 ± 0.2	−10.3 ± 0.3
(GlcNAc)_3_	1	0.09 ± 0.05	−4.7 ± 0.1	−5.0 ± 0.3	−9.7 ± 0.3
(GlcNAc)_4_	1	0.40 ± 0.24	−4.8 ± 0.6	−4.0 ± 1.0	−8.8 ± 0.3

Thermodynamic parameters for WT were obtained by ITC experiments. The titration conditions for the tested chitooligosaccharides and the *Vh*CBP variants are described in text. All thermodynamic values presented in Figure 6*A* are the mean ± SD, which were obtained from three separate sets of ITC experiments.

### Crystal structures of *Vh*CBP in complex with (GlcNAc)_3_ or (GlcNAc)_4_

**Figure 2:**
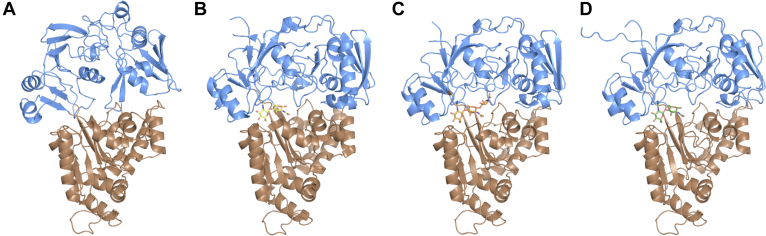
***Cartoon* representations of the structures of *Vh*CBP variants.***A*, apo-*Vh*CBP model and structures of *Vh*CBP in complex with (*B*) (GlcNAc)_2_, (*C*) (GlcNAc)_3_, and (*D*) (GlcNAc)_4_. The *upper domain* is colored in *blue* and the *lower domain* in *brown*. The bound (GlcNAc)_n_ (n = 2, 3, and 4) are represented by *stick* models colored in *yellow*, *orange*, and *green*, respectively. The apo structure model shown in *A* was obtained by homology modeling using the *Vc*CBP structure (4GF8) as a template. *Vc*CBP, CBP from *Vibrio cholerae*; *Vh*CBP, CBP from *Vibrio campbellii* type strain ATCC BAA-1116.

In the previous article, we reported the mode of binding of (GlcNAc)_2_ to *Vh*CBP, based on the crystal structure (13). To further analyze the sugar residue interactions in the groove extending beyond the (GlcNAc)_2_-binding site, we solved the crystal structures of *Vh*CBP in complex with (GlcNAc)_3_ and (GlcNAc)_4_. Structural statistics are listed in Table 2. The resolutions of the structures range from 1.5 to 1.8 Å. Figure 2 shows the bound *Vh*CBP structures with (GlcNAc)_n_ (n = 2, 3, and 4) and the apo-*Vh*CBP structure model, obtained by homology modeling using the apo-structure of *Vc*CBP (1ZTY). The overall modeled structure of apo-*Vh*CBP (Fig. 2*A*) was different from those of *Vh*CBP with bound (GlcNAc)_2_, (GlcNAc)_3_, or (GlcNAc)_4_ (Fig. 2, *B*–*D*, respectively), especially in the upper domain colored in blue. The three ligand-bound structures were essentially identical to each other; however, minor conformational differences between the (GlcNAc)_2_-bound and (GlcNAc)_3_/(GlcNAc)_4_-bound structures were found at the GlcNAc-binding site of the lower domain (colored in brown).

**Table 2:** X-ray data collection and refinement statistics for *Vh*CBP variants in complex with (GlcNAc)_2–4_

VhCBP variant	WT	WT	W363A	N409A	F411A	F437A	W513A
Ligand	(GlcNAc)_3_	(GlcNAc)_4_	(GlcNAc)_2_	(GlcNAc)_2_	(GlcNAc)_2_	(GlcNAc)_2_	(GlcNAc)_2_
PDB code	6LZQ	7EBI	7EBM	6LZT	6LZU	6LZV	6LZW
Data collection							
Space group	*P*12_1_1	*P*12_1_1	*P*12_1_1	*P*12_1_1	*P*12_1_1	*P*12_1_1	*P*12_1_1
Molecule/asymmetric unit	1	1	1	1	1	1	1
Unit-cell parameters							
a, b, c (Å)	61.8, 56.8, 81.9	61.9, 57.1, 82.1	58.6, 56.0, 80.3	59.5, 56.4, 80.9	62.3, 57.2, 82.0	59.5, 57.0, 85.2	61.7, 57.1, 81.5
α, β, γ (°)	90.0, 99.4, 90.0	90.0, 99.5, 90.0	90.0, 101.8, 90.0	90.0, 101.0, 90.00	90.0, 100.5, 90.0	90.0, 101.4, 90.0	90.0, 101.7, 90.0
Wavelength (Å)	0.99984						
Resolution range (Å)	50.0–1.80 (1.83–1.80)	50.0–1.50 (1.53–1.50)	50.0–2.00 (2.03–2.00)	50.0–1.85 (1.88–1.85)	50.0–1.90 (1.93–1.90)	20.0–2.20 (2.24–2.20)	50.0–1.90 (1.93–1.90)
Completeness (%)	92.2 (85.5)	97.8 (95.7)	99.9 (100.0)	99.8 (100.0)	98.2 (91.8)	99.6 (95.2)	95.0 (91.7)
*R*_merge_	0.128 (1.114)	0.065 (0.396)	0.184 (1.863)	0.112 (0.687)	0.105 (0.552)	0.066 (0.593)	0.105 (0.757)
*R*_*pim*_	0.085 (0.765)	0.040 (0.312)	0.075 (0.796)	0.067 (0.423)	0.068(0.444)	0.040 (0.376)	0.075 (0.630)
Multiplicity	3.2 (2.9)	3.4 (2.3)	6.9 (5.8)	3.7 (3.6)	3.2 (2.1)	3.7 (3.3)	2.9 (2.5)
*I*/*σ*(*I*)	8.8 (0.8)	20.0 (2.6)	20.4 (2.1)	27.0 (1.9)	10.8 (1.4)	18.5 (1.8)	9.8 (4.2)
Refinement							
Resolution range (Å)	36.5–1.80	24.5–1.50	23.7–1.90	23.6–1.85	24.1–1.90	19.9–2.20	26.9–1.90
No. of reflections used	43,328	87,087	38,780	44,261	40,989	26,982	38,988
*R*_work_/*R*_free_	0.164/0.206	0.174/0.192	0.183/0.249	0.236/0.296	0.186/0.234	0.171/0.225	0.221/0.289
No. of atoms							
Protein	4301	4338	4250	4289	4158	4301	4156
Ligand	118	81	32	29	41	33	33
Ion	8	8	4	2	4	6	3
Water	337	308	261	135	256	133	134
RMSD							
Bonds (Å)	0.007	0.006	0.008	0.008	0.008	0.009	0.008
Angles (°)	1.052	1.067	1.073	1.115	1.037	1.162	1.134
Ramachandran plot							
Favored (%)	96.5	97.8	96.8	95.3	96.2	94.04	93.5
Allowed (%)	3.2	2.2	3.0	4.5	3.4	5.59	5.6
Disallowed (%)	0.4	0.0	0.2	0.2	0.4	0.37	1.0

**Figure 3:**
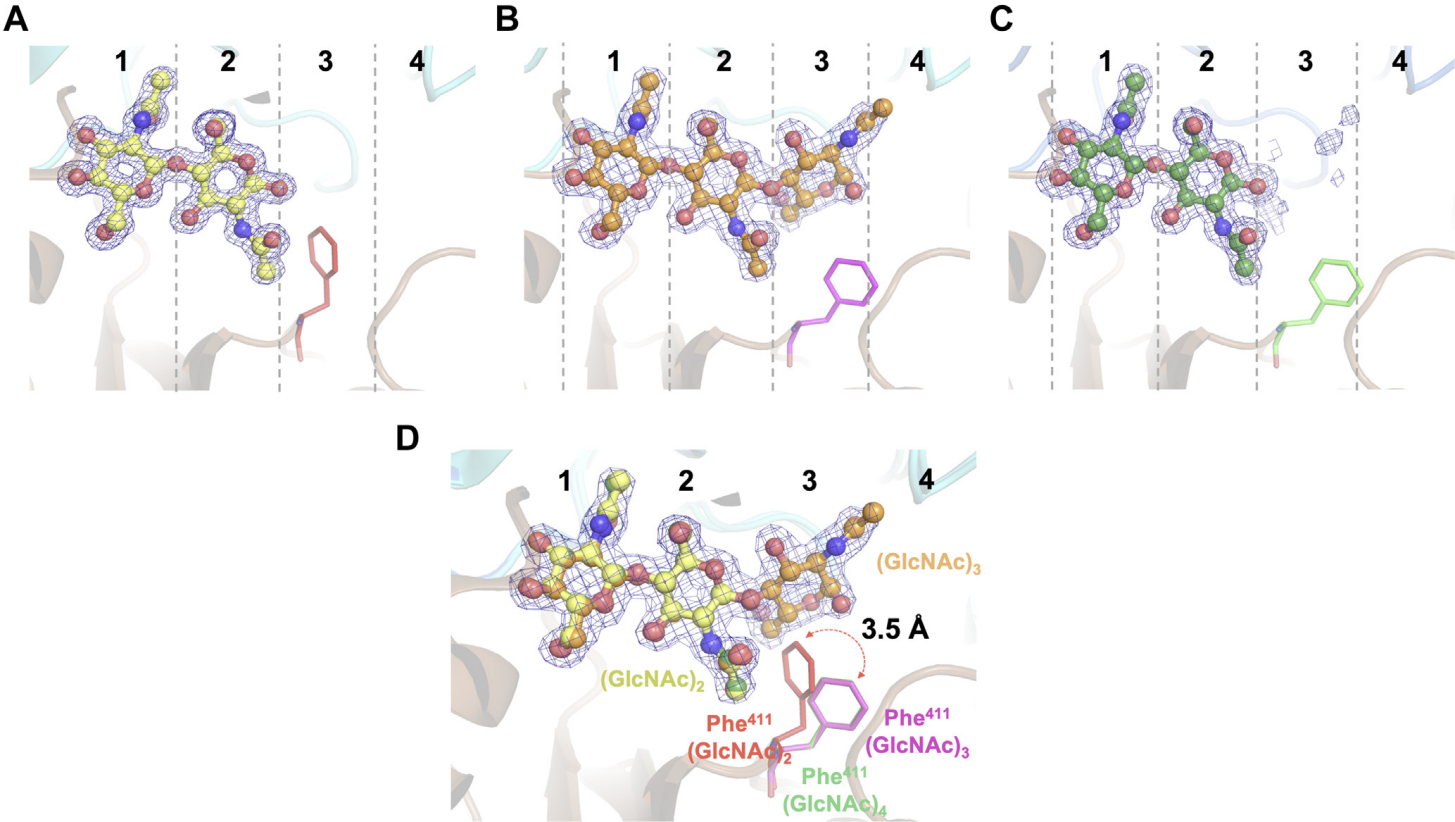
**Close-up view of bound (GlcNAc)**_**n**_**(n = 2, 3, and 4) in the binding groove of *Vh*CBP.***A*, (GlcNAc)_2_ (5YQW), (*B*) (GlcNAc)_3_ (6LZQ), and (*C*) (GlcNAc)_4_ (7EBI). The molecules of (GlcNAc)_n_ in the complex structures are represented by *stick* models colored *yellow* for (GlcNAc)_2_, *orange* for (GlcNAc)_3_, and *forest green* for (GlcNAc)_4_. The Phe^411^ side chains are depicted in *stick* models and colored *brown* for the *Vh*CBP–(GlcNAc)_2_ complex, *magenta* for *Vh*CBP–(GlcNAc)_3_, and *green* for (GlcNAc)_4_. The affinity subsites are designated by numbering from the inner (left)-most site (1, 2, 3, and 4). The 2*F*_o_-*F*_c_ map at *σ* = 1.0 (*blue*) around ligand molecules is depicted in a *mesh* representation. The main-chain structure of *Vh*CBP is represented by a *cartoon* model colored *marine blue* for the *upper domain* and *brown* for the *lower domain*. *D*, superimposition of the structures of *Vh*CBP in complex with (GlcNAc)_2_, (GlcNAc)_3_, or (GlcNAc)_4_. The Phe^411^ side chain flips away from the bound GlcNAc by 3.5 Å upon binding of (GlcNAc)_3_ and (GlcNAc)_4_, probably due to steric hindrance of the bound GlcNAc at affinity site 3. *Vh*CBP, CBP from *Vibrio campbellii* type strain ATCC BAA-1116.

Close-up views of the bound (GlcNAc)_n_ are shown in Figure 3. The arrangement of amino acid residues surrounding (GlcNAc)_3_ or (GlcNAc)_4_ was almost identical to that in the *Vh*CBP–(GlcNAc)_2_ complex (13). (GlcNAc)_2_ was found at affinity sites 1 and 2 in the protein’s binding groove (Fig. 3*A*), where the nonreducing end of the dimer occupied the inner-most affinity site 1, while the reducing end was found at the outer affinity site 2. The electron density of (GlcNAc)_3_ (Fig. 3*B*) in the *Vh*CBP–(GlcNAc)_3_ complex was clearly defined at the affinity sites 1, 2, and 3. The main-chain carbonyl of Ala^514^ was found to form a hydrogen bond with O6 of the reducing-end GlcNAc of the bound (GlcNAc)_3_. By contrast, the electron density of the reducing-end two GlcNAc residues of the bound (GlcNAc)_4_ was weak and uninterpretable (Fig. 3*C*) at the imaginary sites 3 and 4.

The absence of electron density of pyranose rings may be caused by the disorder of the two GlcNAc residues on the reducing-end side. In fact, the 2*F*o-*F*c map of the reducing-end GlcNAc of the bound (GlcNAc)_3_ was also patchy, allowing only partial occupancy of this sugar ring to be set (Fig. S1). Figure 3*D* shows the superimposition of the complex structures of *Vh*CBP–(GlcNAc)_2_, *Vh*CBP–(GlcNAc)_3_, and *Vh*CBP–(GlcNAc)_4_. The GlcNAc residues at affinity sites 1 and 2 overlapped completely with each other, indicating identical conformations of the bound two GlcNAc residues at these sites. We noticed that the glycosidic link between the two GlcNAc residues at affinity sites 2 and 3 was twisted, indicating that the link may have a higher conformational energy than that of the neighboring glycosidic link between sites 1 and 2. As noted above, the aromatic side chain of Phe^411^ is flipped away from the bound GlcNAc by 3.5 Å in the complexed structures with (GlcNAc)_3_ or (GlcNAc)_4_ with full occupancy.

### Binding mode of (GlcNAc)_4_ to *Vh*CBP predicted by docking simulation

**Figure 4:**
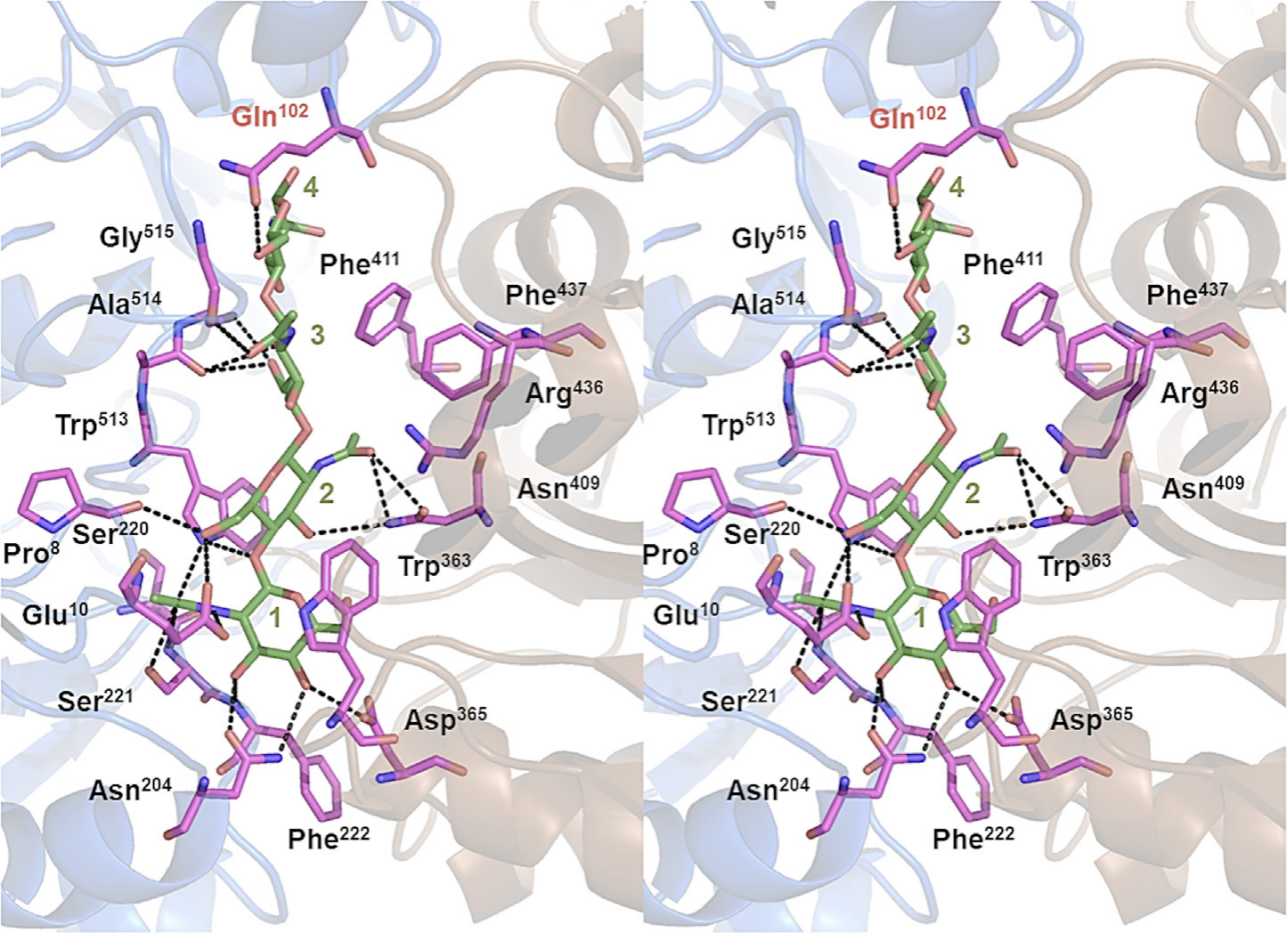
**Stereo view of the modeled structure of *Vh*CBP in complex with (GlcNAc)**_**4**_**.** The (GlcNAc)_4_ structure was docked into the crystal structure of *Vh*CBP in complex with (GlcNAc)_4_ (7EBI) using AutoDock Vina. *Upper* (*marine*) and *lower* (*brown*) domains of the *Vh*CBP molecule are differently shown as a *cartoon* model. The bound (GlcNAc)_4_ (*forest green*) is shown in a *ball-and-stick* model, and amino acid residues directly interacting with (GlcNAc)_4_ (*magenta*) are shown in *stick* models. An amino acid interacting with the reducing-end GlcNAc of the bound (GlcNAc)_4_ is labeled *red*. *Vh*CBP, CBP from *Vibrio campbellii* type strain ATCC BAA-1116.

To predict the structure of the two missing reducing-end residues of bound (GlcNAc)_4_, we conducted docking simulation using AutoDock Vina. Among nine conformers, the most stable provided −10.9 kcal mol^−1^ of binding energy. However, the dihedral bond angle, defined by atoms O5-C1-O1-C4 at the glycosidic bond between the GlcNAc residues at the affinity sites 3 and 4, was −23.3°. This value is far smaller than those of the most stable conformations reported previously (about −90°) (21). Therefore, we selected the second-most stable conformer, with −10.5 kcal mol^−1^ of binding energy, which exhibited reasonable dihedral angles ranging from −96.4° to −73.5°. Figure 4 shows the stereo view of the simulated complex structure.

### ITC binding experiments

**Figure 5:**
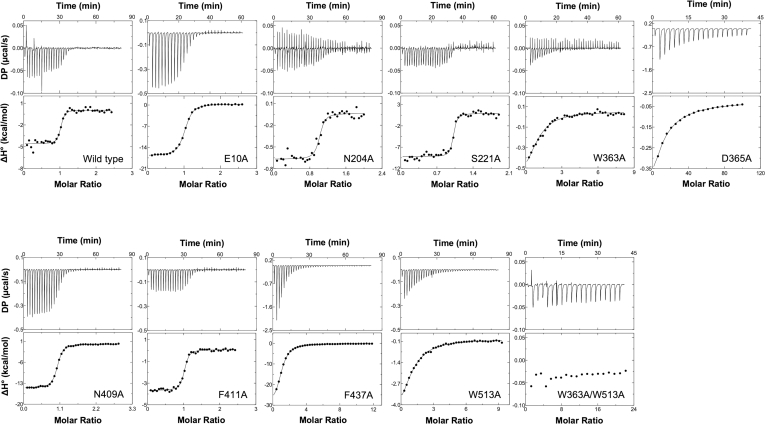
**ITC profiles obtained from (GlcNAc)**_**2**_**titration using *Vh*CBP WT and mutants.** Thermograms (*upper*) and binding isotherms with theoretical fits (*lower*) of the individual titrations are presented. Titration experiments were conducted in 20 mM Tris HCl, pH 8.0, at 25 °C. ITC, isothermal titration calorimetry. *Vh*CBP, CBP from *Vibrio campbellii* type strain ATCC BAA-1116.

**Figure 6:**
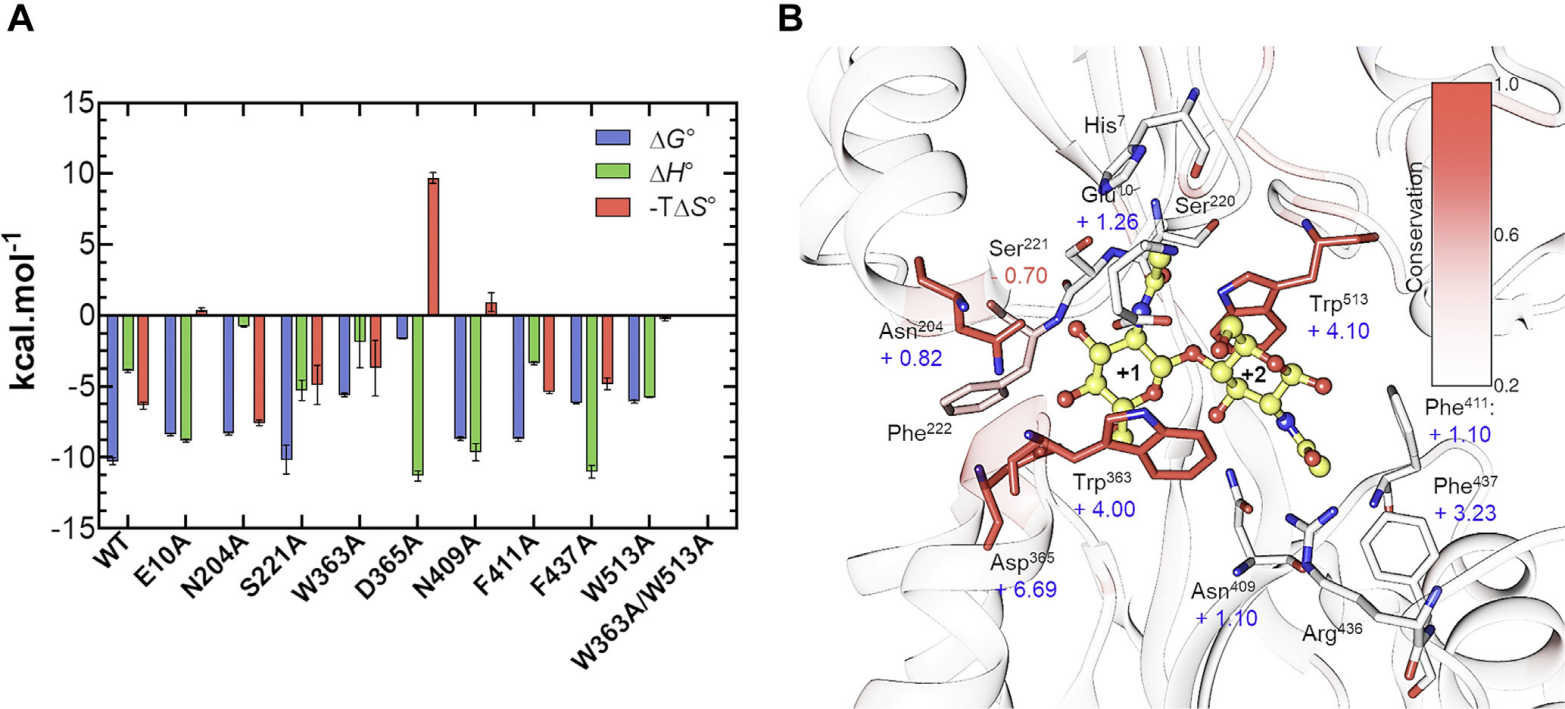
**Thermodynamic signature and degrees of conservation of the sugar-binding residues.***A*, bar diagrams representing Δ*G*°, Δ*H*°, and −*T*Δ*S*° for individual *Vh*CBP mutants. Individual parameter values were obtained from the ITC data shown in Figure 5 and are depicted in *blue*, *green*, and *red*, respectively. *B*, relationship between reductions in binding affinity by mutations (ΔΔ*G*°) and degree of conservation of individual mutated amino acids. ΔΔ*G*° values are stated near the individual mutated amino acids. The mutated amino acids are colored according to the degree of conservation with brightness of *red*, with color labeled 0.2 for modestly conserved to 1 for totally conserved. The degree of amino acid conservation was calculated by dividing the number of the conserved residues in the sequence alignment by the total number of the corresponding residues in the aligned sequences (Fig. 1). The indicator of the degree of conservation is shown on the *upper-right* region of this figure. Values presented in Figure 6*A* are the mean ± SD, which were obtained from three separate sets of ITC experiments. ITC, isothermal titration calorimetry; *Vh*CBP, CBP from *Vibrio campbellii* type strain ATCC BAA-1116.

To evaluate the contributions of the amino acid residues in (GlcNAc)_2_ binding, we carried out site-directed mutagenesis to substitute the designated residues and successfully produced nine single mutants, E10A, N204A, S221A, W363A, D365A, N409A, F411A, F437A, and W513A, and one double mutant, W363A/W513A. All proteins were purified to homogeneity by Ni-NTA affinity chromatography, followed by gel filtration, and their purities were checked by SDS-PAGE analysis (Fig. S2). These proteins were used for ITC binding experiments with (GlcNAc)_2_ as the ligand. The ITC results are shown in Figure 5, and the thermodynamic parameters are listed in Table 3. The bar diagrams of the thermodynamic parameters, Δ*G*°, Δ*H*°, and −*T*Δ*S*°, are also shown in Figure 6*A*. All binding interactions showed exothermic profiles (Fig. 5), but the heat generation varied in different mutants. For the double mutant W363A/W513A, (GlcNAc)_2_ titration did not exhibit any clear heat release. Exceptional affinity changes (ΔΔ*G*°) were observed for the W363A, W513A, and D365A mutants. F437A also showed a relatively large change in the binding affinity. The effects on Δ*G*° in the other mutants (E10A, N204A, S221A, N409A, and F411A) were moderate, although one mutation had been introduced into an amino acid with a high degree of conservation (Asn^204^). Changes in Δ*G*° (ΔΔ*G*°) resulting from all the mutations were mapped on the structure of *Vh*CBP in complex with (GlcNAc)_2_ as shown in Figure 6*B*. Degrees of conservation among five members of cluster/subcluster C-IV SBPs (*Vh*CBP, *Vc*CBP, *Tm*CeBP, *Tm*MnBP1, and *Tm*MnBP2) are also represented by coloring dark red (high degree of conservation) to pale red (moderate degree of conservation) to white (low degree of conservation). As seen from this figure, the effects of mutations on the changes in the binding energy (ΔΔ*G*°) were not always consistent with the degrees of conservation, especially in Asn^204^ and Phe^437^.

**Table 3:** Thermodynamic parameters obtained for (GlcNAc)_2_ binding to *Vh*CBP variants

*Vh*CBP variant	n	*K*_d_ (μM)	Δ*H*^*ο*^ (kcal.mol^−1^)	−*T*Δ*S*^*ο*^ (kcal.mol^−1^)	Δ*G*^*ο*^ (kcal.mol^−1^)	ΔΔ*G*^*ο*^ (kcal.mol^−1^)
WT	1	0.03 ± 0.01	−3.9 ± 0.1	−6.4 ± 0.2	−10.3 ± 0.3	-
E10A	1	0.71 ± 0.07	−8.8 ± 0.1	0.4 ± 0.2	−8.4 ± 0.1	+1.9 ± 0.2
N204A	1	0.83 ± 0.17	−0.75 ± 0.05	−7.6 ± 0.2	−8.3 ± 0.1	+2.1 ± 0.3
S221A	1	0.07 ± 0.06	−5.3 ± 0.7	−4.9 ± 1.4	−10.2 ± 1.0	+0.1 ± 1.0
W363A	1	80.20 ± 19.9	−1.9 ± 1.8	−3.7 ± 2.0	−5.6 ± 0.1	+4.7 ± 0.4
D365A	1	65,033 ± 4550	−11.3 ± 0.4	9.7 ± 0.4	−1.6 ± 0.0	+8.6 ± 0.3
N409A	1	0.45 ± 0.1	−9.6 ± 0.6	1.0 ± 0.7	−8.7 ± 0.1	+1.6 ± 0.2
F411A	1	0.40 ± 0.07	−3.3 ± 0.1	−5.4 ± 0.1	−8.7 ± 0.1	+1.6 ± 0.3
F437A	1	29.73 ± 0.5	−11.0 ± 0.4	−4.8 ± 0.4	−6.2 ± 0.0	+4.1 ± 0.3
W513A	1	36.83 ± 7.4	−5.8 ± 0.0	−0.3 ± 0.1	−6.1 ± 0.1	+4.2 ± 0.3
W363A/W513A	ND	-	-	-	-	-

ND represents no detectable binding.

*Vh*CBP variants were titrated with (GlcNAc)_2_ under the conditions given in Experimental procedures. Thermodynamic values (*K*_d_, Δ*G*^*ο*^, Δ*H*^*ο*^, −*T*Δ*S*^*ο*^) are derived from the corresponding thermograms shown in Figure 5 and are presented as the mean ± SD, which were obtained from three separate sets of experiments.

### Crystal structures of the *Vh*CBP mutants in complex with (GlcNAc)_2_

**Figure 7:**
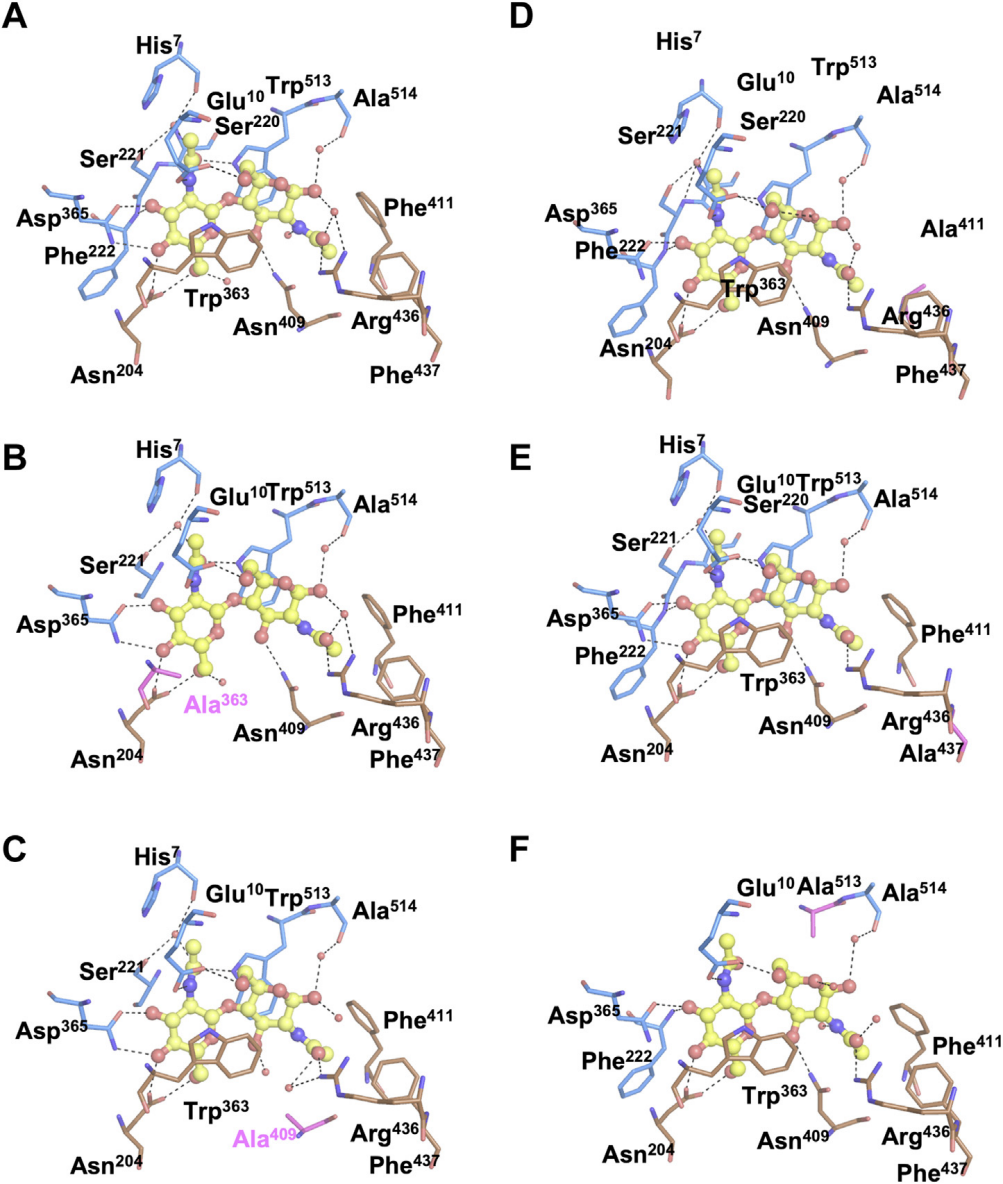
**Close-up views of the (GlcNAc)**_**2**_**-bound crystal structures.***A*, WT, (*B*) W363A, (*C*) N409A, (*D*) F411A, (*E*) F437A, and (*F*) W513A. (GlcNAc)_2_ is shown in a *ball-and-stick* model and colored *yellow*. The sugar-binding residues from the *upper domain* are shown as *sticks* and colored *marine blue*, while those from the *lower domain* are colored *brown*. *Broken lines* represent possible hydrogen bonds.

To explore the effects of mutations on the structure, the mutated *Vh*CBPs were co-crystallized with (GlcNAc)_2_ and single crystals selected for X-ray diffraction experiments. It should be mentioned that the quality of the crystals of E10A, N204A, S221A, D365A, and E363A/W513A in complex with (GlcNAc)_2_ was insufficient for X-ray diffraction experiments, and we failed to obtain high-quality datasets for structural determination. Although crystals for all the other mutants were grown under similar conditions, some variations in the crystal packing were observed (Table 2). Individual structures were solved with the space group of *P*12_1_1. Close-up views of the (GlcNAc)_2_-binding site of the WT and mutated *Vh*CBPs are shown in Figure 7. Compared with the WT *Vh*CBP (Fig. 7*A*), W363A not only eliminated the CH-π stacking interaction with the nonreducing end GlcNAc (Fig. 7*B*) but also slightly shifted the Trp^513^ side chain and the bound (GlcNAc)_2_ (Fig. S3). These shifts may have altered the interaction of GlcNAc with amino acid side chains from the upper domain, including Trp^513^. The N409A mutation eliminated the hydrogen bond with the reducing-end GlcNAc, but simultaneously, two water molecules fit into the corresponding position (Fig. 7*C*). One water molecule interacts with O3 of the reducing-end GlcNAc, whereas the other bridges the *N*-acetyl carbonyl group and Nη of Arg^436^. The F411A mutation did not affect the hydrogen bonds but eliminated the hydrophobic interaction with the *N*-acetyl methyl group of the reducing-end GlcNAc (Fig. 7*D*). A similar effect was observed in F437A, that is, the hydrophobic interaction with the *N*-acetyl methyl group of the reducing-end GlcNAc was likely eliminated by the F437A mutation (Fig. 7*E*); however, in this case, the hydrogen bonding network formed by the Arg^436^ side chain was partly affected by the Ala mutation of Phe^437^. As seen in Figure 7*F*, the W513A mutant was found to eliminate the hydrogen bond with the acetamido -NH_2_ of the nonreducing end GlcNAc and the CH-π stacking interaction with the pyranose ring of the reducing-end GlcNAc, most likely causing the strong reduction of the binding affinity (ΔΔ*G*°, +4.2 ± 0.3 kcal mol^−1^).

**Figure 8:**
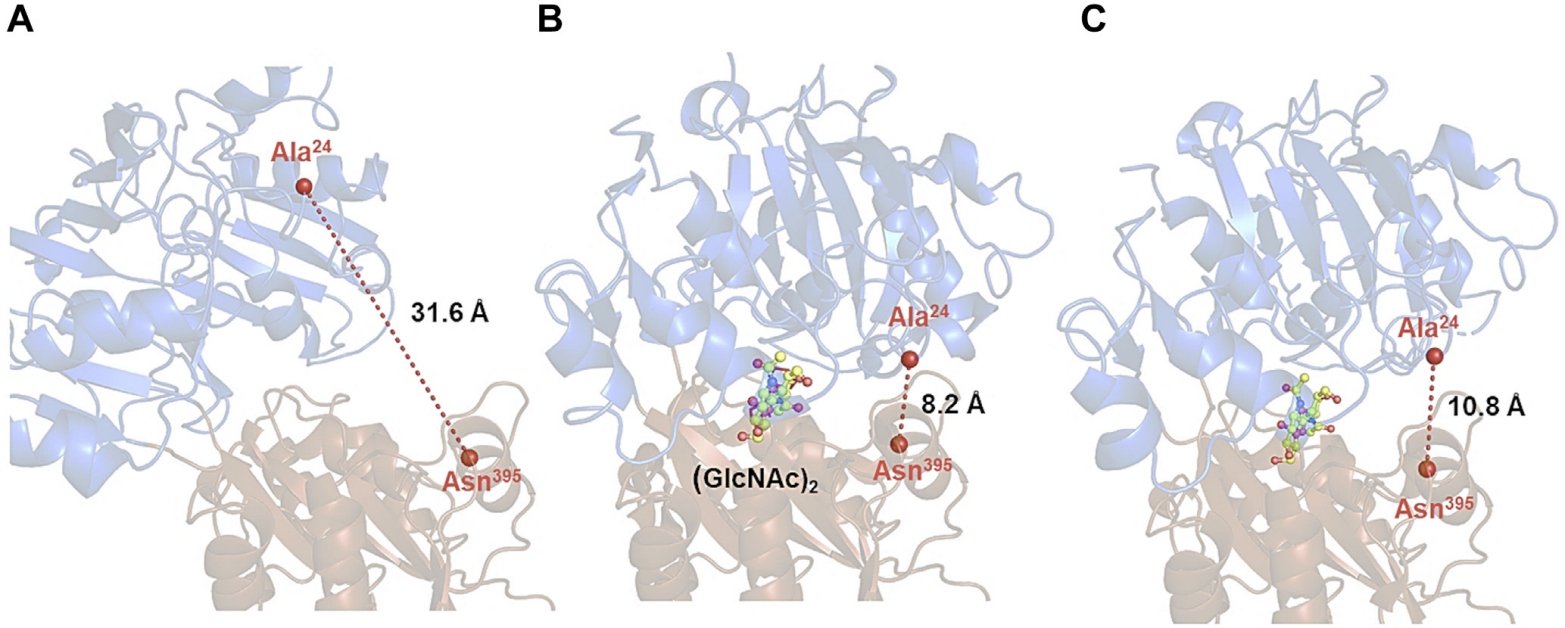
**Structural movement of the WT and W513A mutant in complex with (GlcNAc)**_**2**_**.***A*, apo-WT *Vh*CBP model, (*B*) (GlcNAc)_2_-bound WT *Vh*CBP, and (*C*) (GlcNAc)_2_-bound W513A structures. The *upper domain* is colored *blue* and the *lower domain brown*. The (GlcNAc)_2_ molecule is depicted in a *ball-and-stick* model (*yellow*). Amino acid residues of interest are labeled and depicted as *stick* models. *Broken lines* represent the distances between the corresponding C_α_ atoms, which are represented as bigger *red spheres*. Numerals 1 and 2 represent the affinity sites 1 and 2, respectively. *Vh*CBP, CBP from *Vibrio campbellii* type strain ATCC BAA-1116.

A remarkable structure was seen with W513A, in which the substrate-binding groove was partially open at the reducing-end side (site 2). The distance between *C*_α_ carbons of Ala^24^ and Asn^395^ located on the upper and lower domains of the apo WT *Vh*CBP (homology structure) was 31.6 Å (‘fully open’, Fig. 8*A*), while in WT *Vh*VBP in complex with (GlcNAc)_2_, it was 8.2 Å (‘fully closed’, Fig. 8*B*) and in W513A in complex with (GlcNAc)_2_, it was 10.8 Å (‘half-open’, Fig. 8*C*). The domain movement of W513A released bound water molecules trapped by several hydrogen bonds with the main-chain carbonyl of His^7^, the main-chain -NH of Ser^220^, the side chain Oγ of Ser^221^, and the N-acetyl carbonyl group of the nonreducing end GlcNAc, while the water molecules were retained in the other mutant structures. This conformational state of *Vh*CBP was designated a ‘half-open’ conformation.

## Discussion

### The mode of binding of (GlcNAc)_3_ and (GlcNAc)_4_ to *Vh*CBP

In most carbohydrate-related proteins that have long and extended binding grooves, the binding affinities depend strongly on the degree of polymerization of the oligosaccharide substrates (22); the higher the polymerization degree of the oligosaccharide, the greater the binding affinity. However, we report here that *Vh*CBP binds (GlcNAc)_2_, (GlcNAc)_3_, and (GlcNAc)_4_ with Δ*G*° values of −10.3 ± 0.3, −9.7 ± 0.3, and −8.8 ± 0.3 kcal mol^−1^, respectively (Table 1). The binding affinity did not correspond to the degree of polymerization and was the lowest in the longest ligand, (GlcNAc)_4_. To rationalize the unique dependency on the degree of polymerization of ligands, we analyzed the crystal structures of *Vh*CBP in complex with (GlcNAc)_3_ and (GlcNAc)_4_ (Figs. 2 and 3). A twist of the glycosidic linkage was observed between the GlcNAc residues at the affinity sites 2 and 3 of the bound (GlcNAc)_3_ (Fig. 3*B*), suggesting a potential loss in the stabilization energy of the complexed structure. Furthermore, the Phe^411^ side chain was found to flip away from the bound GlcNAc (3.5 Å) in the complex with (GlcNAc)_3_ (Fig. 3*D*). A water molecule interacting with the carbonyl group of Ala^514^ through a hydrogen bond is replaced by O6 of the reducing-end GlcNAc (affinity site 3) in the *Vh*CBP–(GlcNAc)_3_ complex. This can be seen from the modeled structure of the *Vh*CBP–(GlcNAc)_4_ complex (Fig. 4). No other interactions were found in the third GlcNAc, indicating only a weak interaction of the GlcNAc at site 3. This was confirmed from the patchy electron density of the reducing-end GlcNAc (Fig. S1), suggesting flexibility of this GlcNAc. The enthalpy/entropy gain derived from the additional hydrogen bond and the flexible GlcNAc residue at site 3 appear to be almost compensated by the disadvantages incurred by the flipping of the Phe^411^ side chain and twisting of the glycosidic bond, resulting in affinity for (GlcNAc)_3_ that is similar to that for (GlcNAc)_2_.

The electron density of (GlcNAc)_4_ on the reducing-end side was too weak to place the two GlcNAc moieties at the imaginary affinity sites 3 and 4 (Fig. 3*C*). This suggests that the mobility of the fourth sugar has a knock-on effect to weaken the binding of the third sugar. Flipping of the Phe^411^ side chain was also observed in the complex with (GlcNAc)_4_, as in the complex with (GlcNAc)_3_, and appears to widen the space at sites 3 and 4. It is most likely that GlcNAc residues are present at sites 3 and 4 and are disordered because of the large space on these two sites. Docking simulation of the structure of *Vh*CBP in complex with (GlcNAc)_4_ revealed that interactions are formed mostly with the two GlcNAc units at the affinity sites 1 and 2, whereas on sites 3 and 4, only two hydrogen bonds are formed, by the main-chain carbonyl of Ala^514^ and the Oδ side chain of Gln^102^, respectively (Fig. 4). The middle glycosidic linkage of the bound (GlcNAc)_4_ was also twisted, as in (GlcNAc)_3_ bound to *Vh*CBP (Fig. 3*B*). The disadvantage derived from the twist of the glycosidic linkage appears to predominate over the advantages derived from additional interactions/disorder of the GlcNAc residues at sites 3 and 4, resulting in the lower affinity for (GlcNAc)_4_. A similar observation was reported for *Tm*CeBP in complex with cellopentaose, where cellopentaose was bound through water molecules, but not by direct interaction at the same affinity sites (23). A longer and more versatile binding cleft was observed for maltose-binding protein (MBP) from *Escherichia coli* (24, 25). MBP was identified to be able to bind maltooligomers of 2 to 7 glucose units. The crystal structure of MBP in complex with maltodextrin indicated that the strong affinity sites of MBP are more extended to sites S1, S2, and S3, enabling maltotriose to be tightly accommodated (25).

### Major contributions of Asp^365^, Phe^437^, Trp^363^, and Trp^513^

Asp^365^, Trp^363^, and Trp^513^ are completely conserved among the cluster C-VI SBPs (Figs. 1 and 6*B*), suggesting their importance in the binding of sugars to SBPs. We already reported the importance of the two tryptophan residues^16^, which appear to sandwich the oligosaccharide ligand (Fig. 6*B*). The mutational effects of Trp^363^ and Trp^513^ on Δ*G*° were similar (ΔΔ*G*° = +4.7 ± 0.4 and +4.2 ± 0.3 kcal mol^−1^, respectively), but the effects of mutations on enthalpy/entropy contributions are significantly different between the two Trp mutants. The W363A mutation had a large effect on the enthalpic term, while in W513A, a large change was observed in the entropic contribution (Table 3). This can be attributed to the difference in the interaction modes between the two residues. Trp^363^ interacts purely through CH-π stacking, while Trp^513^ interacts through both CH-π stacking and hydrogen bonding with the *N*-acetyl group of the GlcNAc in site 1. As demonstrated from the crystal structure of W363A (Fig. S3), the Trp^513^ side chain is shifted by the mutation of Trp^363^ by 0.6 Å, and the bound (GlcNAc)_2_ also shifted slightly. These rearrangements probably affect the binding interactions provided by amino acids in the upper domain, including Glu^10^, Asn^204^, Ser^221^, and Trp^513^. Similar phenomena may take place on mutation of Trp^513^. These effects may enhance ΔΔ*G*° caused by the mutations of the two important tryptophan residues.

The greatest impact on Δ*G*° was observed in the Asp^365^ mutation (ΔΔ*G*° = +8.6 ± 0.3 kcal mol^−1^). Specifically, this mutation affected the entropic term much more than the enthalpic term, not only eliminating the two hydrogen bonds that were observed in the crystal structure (13) but also affecting the state of solvation. The number of bound water molecules around Asp^365^ was strongly reduced by the mutation to alanine; hence, the entropy gain due to the desolvation upon GlcNAc residue binding was much lower in the D365A mutant than in the WT. This enhanced ΔΔ*G*° in the D365A mutant. By contrast, the reduction in hydrophobicity in the F437A mutation may not only have weakened hydrophobic interaction with the *N*-acetyl methyl group of the reducing-end GlcNAc at the site 2 but also added a conformational restraint to Phe^411^. The F437A mutation may have also affected the hydrogen bond network formed by the nearby amino acid, Arg^436^, with the *N*-acetyl carbonyl group of the same GlcNAc and with a bound water molecule. These subtle changes resulted in a rather large impact on Δ*G*° (ΔΔ*G*° = +4.1 ± 0.3 kcal mol^−1^). It is notable that Phe^437^ is conserved only among the chitin-specific SBPs, that is, *Vc*CBP and *Vh*CBP, and Arg^436^ and Phe^437^ recognize the *N*-acetyl group of the ligand. The specificity toward GlcNAc may be mainly attributed to these two amino acids, indicating that mutational study of Arg^436^ and Phe^437^ may lead to specificity-engineered sugar-specific SBPs. Here, we concluded that the major contributors, Asp^365^, Phe^437^, Trp^363^, and Trp^513^, do not simply interact with GlcNAc but control the state of the surrounding amino acids or bound water molecules.

### Minor contributions of Glu^10^, Asn^204^, Ser^221^, Asn^409^, and Phe^411^

The other mutational effects (of E10A, N204A, S221A, N409A, and F411A) on Δ*G*° were moderate, although one mutation involved the amino acid with the highest degree of conservation, Asn^204^ (Fig. 6*B*). Glu^10^, Asn^204^, and Ser^221^ interact with the GlcNAc residue at site 1 with a complicated hydrogen bonding network in the narrow binding cavity. The effect of eliminating the interactions of one of these amino acids may be compensated by the interactions of the other amino acids located close to the bound (GlcNAc)_2_; for example, the interaction of O4 of the nonreducing GlcNAc is shared by Asn^204^ and Asp^365^, while O3 of the same GlcNAc is shared by Asn^204^ and the main-chain NH of Phe^222^. Thus, the mutational effect of Asn^204^ is compensated by Asp^365^ and Phe^222^. Similar observations were made for Glu^10^ and Ser^221^. A hydrogen bond of Asn^409^ with O3 of the GlcNAc at site 2 is eliminated by the N409A mutation; however, two water molecules were introduced into the *Vh*CBP–(GlcNAc)_2_ interface. This may be the cause of the decreased enthalpic term and increased entropic term listed in Table 3. Finally, the N409A mutation resulted in a small decrease in the binding affinity. Mutation of Phe^411^ lowered the favorable contribution of the enthalpy change, suggesting a role of Phe^411^ in masking the *N*-acetyl group from water molecules, avoiding direct contact with water and forming a hydrophobic interaction with the *N*-acetyl methyl group.

### A ‘ping-pong’ model for (GlcNAc)_2_ translocation by CBPs from *Vibrio* spp.

**Figure 9:**
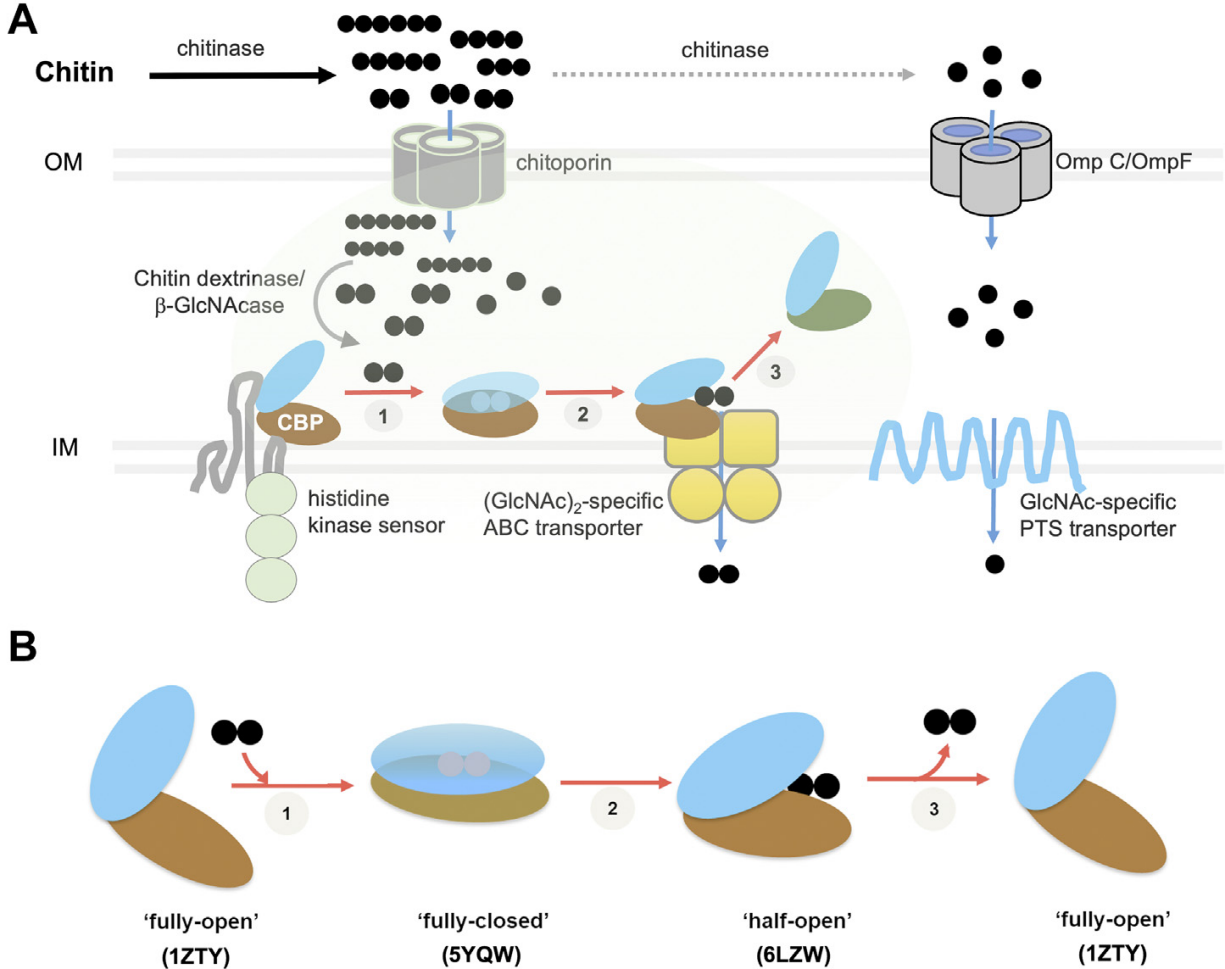
**The structural model for (GlcNAc)**_**2**_**translocation assisted by CBP in the chitin catabolism of *Vibrio* spp.***A*, (GlcNAc)_2_ and other chitooligosaccharides derived from the degradation of environmental chitin by chitinase are transported across outer membrane through chitoporin. (GlcNAc)_2_ and GlcNAc are further generated in the periplasm by chitin dextrinase/β-GlcNAcase (5). Binding of (GlcNAc)_2_ releases CBP from an inner membrane-bound histidine kinase sensor. (GlcNAc)_2_ is translocated to the (GlcNAc)_2_-specific ABC transporter. GlcNAc, on the other hand, is transported across outer membrane through OmpC/OmpF and then into the cytoplasm by a carbohydrate phosphotransferase system-driven permease. The (GlcNAc)_2_ translocation by CBP is proposed as a three-step process (*oval*, *shaded area*); step 1, (GlcNAc)_2_ binds the ‘fully open’ form of CBP and induces the ‘fully closed’ form of CBP; step 2, CBP translocates (GlcNAc)_2_ to the (GlcNAc)_2_-specific transporter; step 3, the CBP releases (GlcNAc)_2_ to the transporter. *B*, the ‘ping-pong’ model for the (GlcNAc)_2_ translocation suggested by the crystal structures of CBP in the absence or presence of (GlcNAc)_2._ The ‘fully open’ form was observed for the ligand-free CBP; the ‘fully closed’ form for the CBP tightly bound to (GlcNAc)_2_; and the ‘half open’ form for the CBP loosely bound to (GlcNAc)_2_. *Blue* indicates the *upper lobe* and *dark brown* the *lower lobe*. GlcNAc and chitooligosaccharides are presented in *black*, *filled circles*. CBP, chitooligosaccharide-binding protein.

The chitin catabolic cascade of *Vibrio* spp. was previously proposed by Li and Roseman (5) to be rigorously controlled by the environment signal (GlcNAc)_2_. Figure 9*A* illustrates how (GlcNAc)_2_ plays a central role in the rigorous control of the chitin catabolic cascade. (GlcNAc)_2_ and other chitooligosaccharides are transiently generated from chitin degradation by chitinase (26, 27) and then transported across the outer membrane through ChiP. The final degradation products (GlcNAc)_2_ and GlcNAc are generated by periplasmic enzymes, chitin dextrinase and β-GlcNAcase, respectively (5). Binding of (GlcNAc)_2_ releases the CBP from a membrane-bound histidine kinase sensor (ChiS), which essentially activates the gene cluster that is involved in the downstream cascade. CBP translocates (GlcNAc)_2_ to the (GlcNAc)_2_-specific ABC transporter, for further energy production. An alternative route of chitin catabolism involves GlcNAc transport through a general diffusion porin, followed by (GlcNAc)-specific phosphoenolpyruvate:carbohydrate phosphotransferase system permease. In the chitin catabolic cascade, (GlcNAc)_2_ acts as the intrinsic signaling molecule and a key controller of the chitin catabolic cascade.

Although we failed to obtain the crystal structure of apo-*Vh*CBP, the crystal structure of the apo-*Vc*CBP from *V. cholerae* (1ZTY), which is highly homologous to *Vh*CBP (83% identity) was observed in a ‘fully open’ form (Fig. 8*A*), in which the upper and lower lobes are widened. Binding of (GlcNAc)_2_ induced domain movement, as observed in the crystal structure of *Vh*CBP in complex with (GlcNAc)_2_ (5YQW)^16^ and agreeing with the structure of *Vc*CBP in complex with the same substrate (1ZU0). Such a ‘sugar-induced-fit’ produced a ‘fully closed’ state. The most interesting finding in our study was made with W513A in complex with (GlcNAc)_2_. Trp^513^ is located at the mouth of the GlcNAc-binding groove, and its position is thought to regulate the translocation of (GlcNAc)_2_ to the (GlcNAc)_2_-specific ABC transporter. In fact, the crystal structure of the W513A mutant showed a ‘half-open’ conformation (Fig. 8*C*), which effectively provides a snapshot of the sugar-releasing process that facilitates translocation. An uptake system for an acidic polysaccharide, alginate, in *Sphingomonas* sp. A1 was thoroughly investigated by crystallography (28, 29). The crystal structure of alginate-specific SBP (AlgQ2) in complex with an alginate-specific ABC transporter (AlgM1M2SS) was solved in the presence of alginate oligosaccharide. AlgQ2 and AlgM1M2SS form a tunnel-like binding cavity at their interface, and the nonreducing end of bound alginate oligosaccharide interacts strongly with the inner part (closed end, affinity sites 1 and 2) of the binding cavity in the closed conformation of AlgQ2. However, the reducing-end residue is exposed from the exit of the tunnel-like binding cavity (open end, affinity sites 3, 4, 5…) and the oligosaccharide appears to be translocated into the charged surface of the transmembrane domain of AlgM1M2SS (29). Although the sequence similarity is quite low, the ‘half-open’ conformation of W513A obtained for *Vh*CBP is analogous to AlgQ2 in complex with AlgM1M2SS. *Vh*CBP interacts strongly with (GlcNAc)_n_ at the nonreducing end, while the reducing end interacts loosely or unfavorably with *Vh*CBP. After binding to the corresponding transporter, *Vh*CBP in complex with (GlcNAc)_n_ may undergo a subtle change in its conformation from ‘closed’ to ‘half-open’ by eliminating the interaction with Trp^513^. This half-opening may further enhance the looseness of the interaction, facilitating the translocation of (GlcNAc)_n_. The bound (GlcNAc)_n_ may then be released from the substrate-binding groove, like a ball bouncing off a racket. Similar observations were reported for other oligosaccharide-specific SBPs, such as *Tm*CeBP (23) and *E. coli* MBP (24, 25). This common feature of binding grooves in SBPs suggests an energetic advantage of the ‘half-open’ binding groove on the reducing-end side on the sliding motion of oligosaccharides during sugar translocation.

The structural data obtained from our previous report 16 and current studies together suggest the ‘ping-pong’ model for (GlcNAc)_2_ binding/release as depicted in Figure 9*B*. Such a model involves three steps: step 1, the apo-*Vh*CBP in the ‘fully open’ form binds (GlcNAc)_2_, which enters the substrate-binding groove, its binding inducing a ‘fully closed’ conformation of *Vh*CBP. Step 2, the fully closed conformation subsequently undergoes transition to the intermediate ‘half-open’ conformation, and in step 3, (GlcNAc)_2_ leaves the binding sites, triggering the return of *Vh*CBP to its original open conformation. The crystal structure of the W513A–(GlcNAc)_2_ complex observed in the present report is the first experimental evidence capturing an intermediary conformation between fully closed and fully open conformations in the sugar translocation pathway. Our ITC data suggested the closed (sugar-bound) state has high affinity to the (GlcNAc)_2_ substrate (*K*_d_ of 0.03 μM), while the semiopen state has 1200 lower affinity than the closed state (*K*_d_ of ~37 μM). We propose that this decrease in the binding affinity facilitates the sugar release from the *Vh*CBP to its membrane-transporting partner. In conclusion, the proposed structural model is thought to be a general mechanism in the *Vibrio* system, facilitating the translocation of small chitooligosaccharides from the periplasm to inner membrane-transporting components, with (GlcNAc)_2_ being the preferred molecule, allowing the bacteria to utilize chitin as their source of energy.

## Experimental procedures

### Multiple sequence alignment of SBPs

*Vh*CBP was found to belong to cluster C/subcluster IV (C-IV) (15). Four other SBP members occur in the same cluster/subcluster, including *Vc*CBP from *Vibrio cholera* (PDB code, 4GFR), a *Tm*CeBP (PDB code, 2O7I), and *Tm*MnBP1 and *Tm*MnBP2 (PDB codes, 4PFY and 4PFW, respectively). Amino acid sequences of *Vh*CBP (PDB code 5YQW) and the four well-defined sugar-specific SBPs mentioned above were aligned by MUSCLE (29, 30). The secondary structural elements of *Vh*CBP were integrated and displayed on top of the aligned sequences using the program ESPript *v*. 3.0 (31) (Fig. 1).

### Construction of the mutated proteins of *Vh*CBP

Mutant plasmids were constructed using the previously generated pET23d(+)-*cbp* from *V. harveyi* as a DNA template (13). Mutation was introduced into the WT gene using a site-directed mutagenesis kit (Stratagene). Primers used for site-directed mutagenesis are listed in Table S1. Mutation sites are shown in bold, and genetic codons corresponding to the mutated residues are indicated by single underlining. For the double mutant W363A/W513A, the W363A mutation was introduced using the plasmid encoding W513A mutant as a DNA template. Correct mutations were verified by DNA sequencing service (Macrogen, Inc).

### Expression and purification of WT *Vh*CBP and mutants

The competent cells of *E. coli* Origami (DE3) (Novagen) were transformed with the recombinant plasmids harboring the WT or mutated *Vh*CBP genes. Each transformant was inoculated into the LB medium containing 100 μg ml^−1^ ampicillin and shaken at 37 °C until *OD*_600_ reached 0.8. Protein expression was induced by the addition of 0.05 mM IPTG to the cell culture that had been precooled on ice for 10 min. The IPTG-induced cell culture was shaken at 18 °C for a further 24 h, after which the cell pellet was harvested by centrifugation at 4500 rpm, 4 °C for 45 min, and the separated culture medium was discarded. The cell pellet was resuspended in 20 mM sodium phosphate buffer, pH 7.4, containing 150 mM NaCl, 10% (*v/v*) glycerol, 1 mM PMSF, and 0.1% Triton X-100, and the cells in the suspension were disrupted with an ultrasonic processor (Cole-Parmer Instrument Company, LLC). The cell lysate was harvested by centrifugation at 12,000 rpm at 4 °C for 45 min. For protein purification, the crude cell lysate was applied to a Ni-NTA column equilibrated with 20 mM sodium phosphate, pH 7.4, containing 150 mM NaCl, then washed twice with the buffer containing 10 mM imidazole, and eluted with the buffer containing 100 mM imidazole. Eluted protein fractions were collected and diluted three-fold with the same buffer and applied to a HiTrap Q FF column 5 ml (GE Healthcare). The column was washed twice with 20 mM sodium phosphate buffer, pH 7.4, containing 50 mM NaCl and adsorbed protein then eluted with a linear gradient of 50 to 500 mM NaCl. Protein fractions containing *Vh*CBP or the mutants were collected, concentrated by Amicon Ultra-15 (Millipore), and applied to a HiPrep 16/60 Sephacryl S-100 column (GE Healthcare) equilibrated with 20 mM sodium phosphate buffer, pH 7.4, containing 150 mM NaCl. Proteins were isocratically eluted and fractions collected and analyzed by SDS-PAGE. Fractions containing *Vh*CBP or the mutant were pooled and stored at 4 °C for further analysis. Protein concentration was determined from absorbance at 280 nm, using a molar extinction coefficient calculated from the equation proposed by Pace *et al.* (32).

### ITC binding experiments

ITC experiments were conducted by titrating 200 μM (GlcNAc)_n_ (n = 2, 3, or 4) solutions from the injection syringe into 20 μM WT *Vh*CBP in the sample cell. The titrations were performed in 20 mM Tris-HCl buffer, pH 8.0, by a PEAQ-ITC (Malvern Instruments) using the operational software installed in the equipment. Experimental conditions are summarized in Table S2. Briefly, the temperature in the sample cell was maintained at 25 °C, and the difference in temperature (Δ*T*) between the reference cell and the sample cell was compensated by heaters fitted to the cells. The compensation energy utilized per second was plotted against experimental time. Peak integrations of the individual troughs were replotted against the molar ratio of the ligand to protein. The binding isotherm thus obtained was used for data-fitting analysis using the ‘One-Set of Sites’ model defined in MicroCal PEAQ-ITC Analysis Software to obtain *K*_d_, Δ*G*°, Δ*H*°, and Δ*S*° terms. Stoichiometry *n* was fixed at 1.0 because the crystal structure of *Vh*CBP in complex with (GlcNAc)_n_ clearly indicated a 1:1 (protein:ligand) interaction. Titrations of (GlcNAc)_2_ to the individual *Vh*CBP mutants were performed as described for the WT protein. Because affinity and heat generation changed depending on the protein used, ligand and protein concentrations used for the experiments were varied (Table S2) to fulfill the criteria proposed by Turnbull and Daranas (33).

### Crystallization and structure determination of *Vh*CBP mutants in complex with (GlcNAc)_2_

Crystallization conditions for individual mutants were screened using Morpheus (Molecular Dimensions). Protein solution (10 mg ml^−1^) was freshly prepared in 10 mM Hepes buffer, pH 7.0, containing 100 mM NaCl and mixed with an equal volume of the precipitant solution to make a crystallization drop. Crystal screens were set up by a microbatch-under-oil technique using 100% (*v/v*) silicone oil (Hampton Research) at 20 °C. Single crystals were harvested from crystallization drops by a cryoloop and flash-frozen and stored under liquid nitrogen. *Vh*CBP was successfully cocrystallized with (GlcNAc)_3_ and (GlcNAc)_4_. Although cocrystallization with (GlcNAc)_2_ was attempted with all the mutants produced, only five mutants, W363A, N409A, F411A, F437A, and W513A, were cocrystallized with (GlcNAc)_2_ under the conditions described in Table S3. With the other mutants, crystallization was unsuccessful.

X-ray diffraction experiments were performed at the beamline, TPS-05A, in NSRRC (National Synchrotron Radiation Research Center, Taiwan). An automated system controlled by the software installed in the Linux machines at the beamline handled the crystals during experiments. Briefly, crystals were mounted onto the goniometer automatically by a robotic arm and irradiated with X-ray at *λ* = 0.999840 Å, and the diffraction images were collected by MX300-HS (Rayonix, LLC), integral to the beamline. Diffraction images were analyzed by HKL2000 (34). The resulting intensity file was transformed to structural factor file and phased by molecular replacement using the complex structure of *Vh*CBP and (GlcNAc)_2_ (PDB code, 5YQW) as the template by PHASER MR in CCP4i program suite *v*. 7.0 (35, 36). The modeled structures were refined by a combination of Coot (37) and Phenix.Refine (38, 39, 40, 41) in Phenix 1.9 (42). The statistics of data collection and structural refinement are shown in Table 2. The structures were displayed by PyMOL Molecular Graphics System *v*. 2.4.0. Structures were successfully solved with the highest resolutions below 2.2 Å and deposited in the PDB database under the PDB codes 6LZQ for *Vh*CBP–(GlcNAc)_3_, 7EBI for *Vh*CBP–(GlcNAc)_4_, 7EBM for W363A–(GlcNAc)_2_, 6LZT for N409A–(GlcNAc)_2_, 6LZU for F411A–(GlcNAc)_2_, 6LZV for F437A–(GlcNAc)_2_, and 6LZW for W513A–(GlcNAc)_2_.

### Docking of (GlcNAc)_4_ into *Vh*CBP

Because the electron density of two sugar residues on the reducing-end side of bound (GlcNAc)_4_ was not observed, (GlcNAc)_4_ was modeled into the *Vh*CBP structure extracted from the *Vh*CBP–(GlcNAc)_4_ complex (7EBI), generating the most plausible structure of the *Vh*CBP–(GlcNAc)_4_ complex. AutoDock Vina was used to simulate the complex structure (43, 44). The ligand was prepared using GLYCAM and PRODRG (45, 46). The ligand was docked into the *Vh*CBP molecule using Iterated Local Search global optimizer in AutoDock Vina. Conformers were searched within a grid box with 40, 40, and 30 Å for each dimension covering the entire binding pocket of *Vh*CBP. Finally, the conformation was converged to nine conformers with the energies calculated, −10.9, −10.5, −10.1, −9.3, −9.0, −8.8, −8.6, −8.5, and −8.5 kcal mol^−1^, respectively, from the most stable to unstable conformer.

## Data availability

The atomic co-ordinates and structural factor data of *Vh*CBP have been deposited in the PDB database under the accession codes 6LZQ for WT *Vh*CBP–(GlcNAc)_3_, 7EBI for WT *Vh*CBP–(GlcNAc)_4_, 7EBM for W363A–(GlcNAc)_2_, 6LZT for N409A–(GlcNAc)_2_, 6LZU for F411A–(GlcNAc)_2_, 6LZV for F437A–(GlcNAc)_2_, and 6LZW for W513A–(GlcNAc)_2_. The ITC data are available upon request to Wipa Suginta (wipa.s@vistec.ac.th).

## Supporting information

This article contains supporting information with supplementary experiments (Figs. S1, and S2) and full wwPDB X-ray structure validation reports for all the crystal structures presented in this study.

## Conflict of interest

The authors declare that they have no conflicts of interest with the contents of this article.
