# Supplementary material for: A structural model for (GlcNAc)2 translocation via a periplasmic chitooligosaccharide-binding protein from marine Vibrio bacteria
Source: J Biol Chem. 2021 Aug 13;297(3):101071. doi: 10.1016/j.jbc.2021.101071 (PMC8449061; doi:10.1016/j.jbc.2021.101071)
Supplement: Supplemental Figures S1–S3 and Tables S1–S3 [file mmc1.pdf]

# A structural model for $(GlcNAc)_2$ translocation via a periplasmic chitooligosaccharide binding protein from marine *Vibrio* bacteria

Yoshihito Kitaoku,[1] Tamo Fukamizo,[1*] Sawitree Kumsaoad,[1] Prakayfun Ubonbal,[1]

Robert C Robinson,[1,2*] Wipa Suginta[1*]

[1]School of Biomolecular Science and Engineering (BSE), Vidyasirimedhi Institute of Science and Technology (VISTEC), Payupnai, Wangchan, Rayong 21210 Thailand

[2]Research Institute of Interdisciplinary Science (RIIS), Okayama University, 3-1-1 Tsushimanaka, Kita-ku, Okayama-shi, Okayama 700-8530, Japan

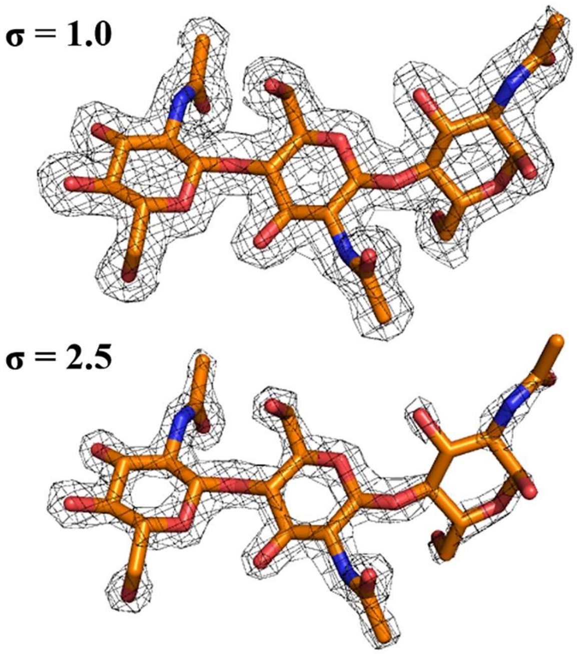


**Fig. S1:** $2F_o$-$F_c$ maps of the bound $(GlcNAc)_3$ in the crystal structure of the *Vh*CBP-$(GlcNAc)_3$ complex. The bound $(GlcNAc)_3$ molecule is represented by stick models colored in orange. The omit maps at σ = 1.0 and 2.5 are depicted by mesh representation (black).

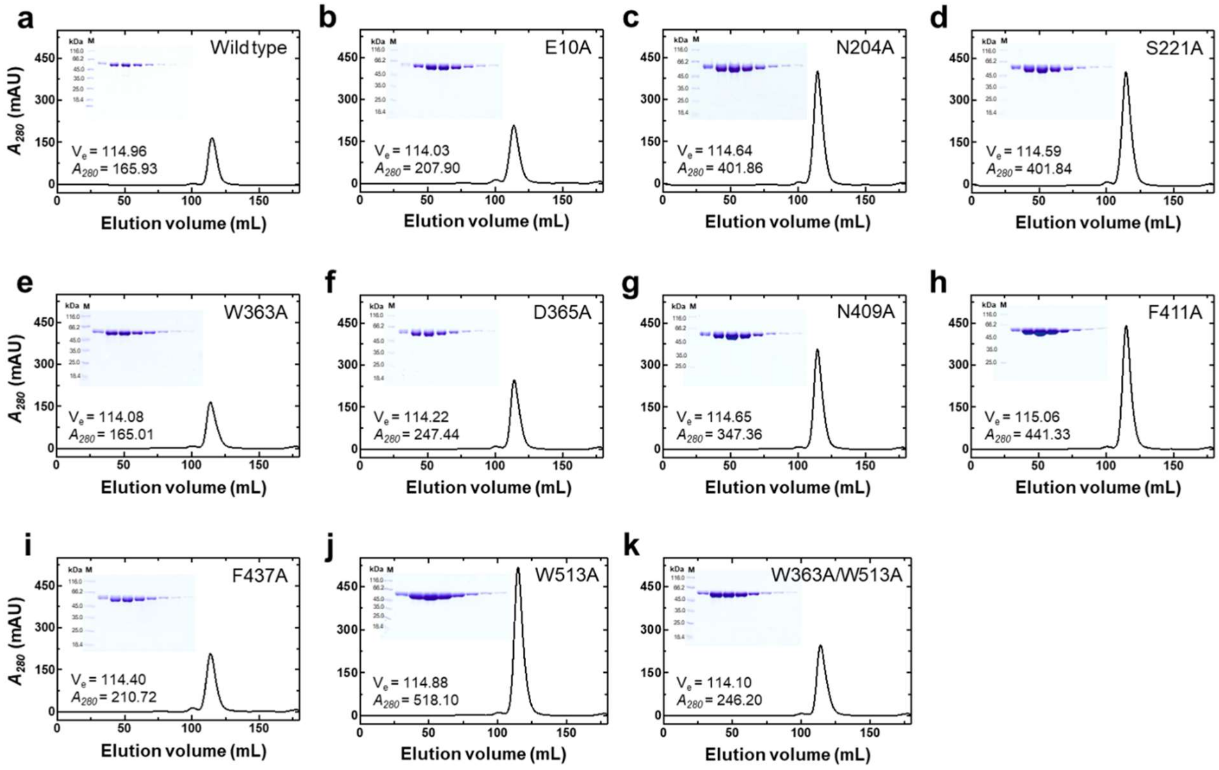


**Fig. S2:** Purification of *Vh*CBP variants and SDS-PAGE analysis. The heterologous *Vh*CBP WT and mutants were expressed and purified by Ni-NTA affinity chromatography, followed gel filtration chromatography on a Superdex™ 200 column, connected with ÄKTA pure FPLC system (see *Experimental* for details). Figures a-k are gel filtration elution profiles with an applied flow rate of 0.5 mL.min$^{-1}$. The purity of the *Vh*CBP containing fractions obtained from the A280 peak were resolved on 12% SDS-PAGE gel. Protein samples are: (a), WT; (b), E10A; (c), N204A; (d), S221A; (e), W363A; (f), D365A; (g), N406A; (h), F411A; (i), F437A; (j), W513A; (k), W363A/W513A. $V_e$ is elution volume (mL).

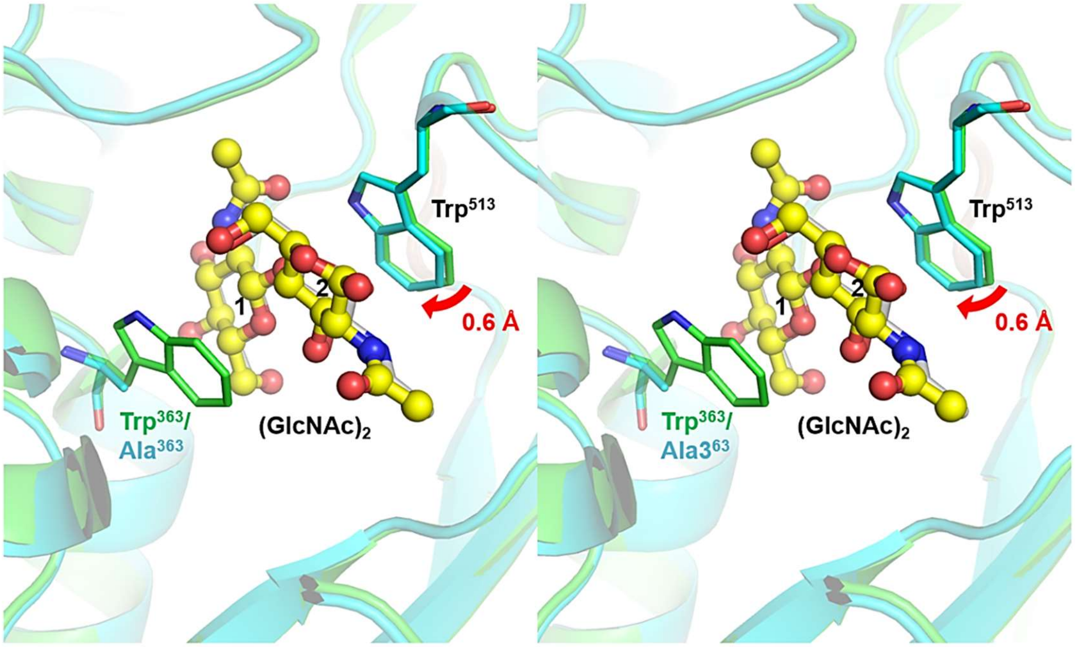


**Fig. S3:** Stereo view of the superimposed crystal structures of *Vh*CBP wild-type (green) and W363A mutant (cyan) in complex with $(GlcNAc)_2$. The $Trp^{513}$ side chain is shifted by 0.6 Å on mutation of $Trp^{363}$. The bound $(GlcNAc)_2$ is also shifted slightly. Stick models of two tryptophan side chains are colored using the same color scheme. $(GlcNAc)_2$ in the individual complex structures are shown by ball-and-stick models colored differently: wild type, white; W363A, yellow. Movement of Trp513 according to the $(GlcNAc)_2$ shift is indicated by arrows (red).

**Table S1: Oligonucleotides used for site-directed mutagenesis of *Vh*CBP.**

| Primer | Sequence |
|---|---|
| E10A Fwd. | 5’-GACTATCCACCCTAAAG**C**GTTTACAACTTTTGTTC-3’ |
| E10A Rev. | 5’-GAACAAAAGTTGTAAAC**G**CTTTAGGGTGGATAGTC-3’ |
| N204A Fwd. | 5’-CGTGTTCCACAAATCGCAAAC**GCA**GACCAATTCCTAGGTAAAG-3’ |
| N204A Rev. | 5’-CTTTACCTAGGAATTGGTC**TGC**GTTTGCGATTTGTGGAACACG-3’ |
| S221A Fwd. | 5’-GATGGACTGGACTTCT**G**CATTCGTTCCAGATATC-3’ |
| S221A Rev. | 5’-GATATCTGGAACGAATG**C**AGAAGTCCAGTCCATC-3’ |
| D365A Fwd. | 5’-CAAACGGTTGGACTG**C**CTTCAACAACACTGTACAG -3’ |
| D365A Rev. | 5’-CTGTACAGTGTTGTTGAAG**G**CAGTCCAACCGTTTG -3’ |
| N409A Fwd. | 5’-ACGATGTTGCGTACACC**GC**CTACTTCCACGGTGCAG-3’ |
| N409A Rev. | 5’-CTGCACCGTGGAAGTAG**GC**GGTGTACGCAACATCGT-3’ |
| F411A Fwd. | 5’-GCGTACACCAACTAC**GC**CCACGGTGCAGACCC-3’ |
| F411A Rev. | 5’-GGGTCTGCACCGTGG**GC**GTAGTTGGTGTACGC-3’ |
| R436A Fwd. | 5’-GGCGACGGTATGCCT**GC**TTTCGCGATGCACTTC-3’ |
| R436A Rev. | 5’-GAAGTGCATCGCGAAA**GC**AGGCATACCGTCGCC-3’ |
| F437A Fwd. | 5’-GGCGACGGTATGCCTCGT**GC**CGCGATGCACTTC-3’ |
| F437A Rev. | 5’-GAAGTGCATCGCG**GC**ACGAGGCATACCGTCGCC-3’ |
| W363A Fwd. | 5’-CAATCGCCAAACGGT**GC**GACTGACTTCAACAACACTG-3’ |
| W363A Rev. | 5’-CAGTGTTGTTGAAGTCAGTC**GC**ACCGTTTGGCGATTG-3’ |
| W513A Fwd. | 5’-GGGCCGTCCAAACATT**GCA**GC**A**GGTATCCCAGAGCGTC-3’ |
| W513A Rev. | 5’-GACGCTCTGGGATACC**T**GC**TGC**AATGTTTGGACGGCCC-3’ |

**Table S2: ITC experimental conditions for titration of *Vh*CBP and its mutants with $(GlcNAc)_{2-4}$.**

| Sample in cell | Conc. in cell (μM) | Sample in syringe | Conc. in syringe (mM) | Buffer | Temperature (K) | Ref. Power (μCal/sec) | Stirring Speed (rpm) | Titration |
|---|---|---|---|---|---|---|---|---|
| WT | 20 | $(GlcNAc)_2$ | 0.2 | 20 mM Tris-HCl, pH 8.0 | 298.15 | 1.0 | 700 | 0.6 μL + 1.0 μL x 39 |
| | | $(GlcNAc)_3$ | 0.2 | | | 1.0 | 700 | 0.6 μL + 1.0 μL x 39 |
| | | $(GlcNAc)_4$ | 0.2 | | | 1.0 | 700 | 0.6 μL + 1.0 μL x 39 |
| | | $(GlcNAc)_2$ | 0.4 | | | 10 | 500 | 0.6 μL + 1.3 μL x 29 |
| E10A | 60 | $GlcNAc)_2$ | 1.0 | | | 10 | 500 | 0.6 μL + 1.0 μL x 39 |
| N204A | 100 | | 0.2 | | | 10 | 500 | 0.4 μL + 0.5 μL x 39 |
| S221A | 20 | | 50 | | | 10 | 500 | 0.6 μL + 2.0 μL x 19 |
| D365A | 100 | | 0.5 | | | 10 | 500 | 0.6 μL + 1.0 μL x 39 |
| N409A | 60 | | 0.8 | | | 10 | 500 | 0.6 μL + 1.0 μL x 39 |
| F411A | 80 | | 2.0 | | | 10 | 500 | 0.6 μL + 1.0 μL x 39 |
| F437A | 100 | | 2.0 | | | 1.0 | 700 | 0.4 μL + 1.0 μL x 39 |
| W363A | 50 | | 2.0 | | | 1.5 | 700 | 0.4 μL + 1.0 μL x 39 |
| W513A | 50 | | 10 | | | 1.0 | 700 | 0.4 μL + 2.0 μL x 19 |

**Table S3: Conditions for co-crystallization of *Vh*CBP mutants with $(GlcNAc)_2$.**

| Complex | Conditions |
|---|---|
| W363A-$(GlcNAc)_2$ | 0.06 M Divalents, 0.1 M Buffer System 2, pH 7.5, 50 % v/v Precipitant Mix 4 |
| N409A-$(GlcNAc)_2$ | 0.06 M Divalents, 0.1 M Buffer System 1, pH 6.5, 50 % v/v Precipitant Mix 4 |
| F411A-$(GlcNAc)_2$ | 0.06 M Divalents, 0.1 M Buffer System 1, pH 6.5, 30 % v/v Precipitant Mix 2 |
| F437A-$(GlcNAc)_2$ | 0.06 M Divalents, 0.1 M Buffer System 2, pH 7.5, 50 % v/v Precipitant Mix 4 |
| W513A-$(GlcNAc)_2$ | 0.06 M Divalents, 0.1 M Buffer System 1, pH 6.5, 30 % v/v Precipitant Mix 2 |
