# Supplementary material for: A structural model for (GlcNAc)2 translocation via a periplasmic chitooligosaccharide-binding protein from marine Vibrio bacteria
Source: J Biol Chem. 2021 Aug 13;297(3):101071. doi: 10.1016/j.jbc.2021.101071 (PMC8449061; doi:10.1016/j.jbc.2021.101071)
Supplement: Supplemental Data 1 [file mmc2.pdf]

Full wwPDB X-ray Structure Validation Report (i)

Dec 22, 2020 – 01:42 PM JST

PDB ID	:	6LZQ
Title	:	Chitin-specific solute binding protein from Vibrio harveyi in complex with chitotriose.
Deposited on	:	2020-02-19
Resolution	:	1.80 Å(reported)

This is a Full wwPDB X-ray Structure Validation Report.

This report is produced by the wwPDB biocuration pipeline after annotation of the structure.

We welcome your comments at *validation@mail.wwpdb.org*
A user guide is available at
https://www.wwpdb.org/validation/2017/XrayValidationReportHelp
with specific help available everywhere you see the (i) symbol.

The following versions of software and data (see references (i)) were used in the production of this report:

MolProbity	:	4.02b-467
Mogul	:	1.8.5 (274361), CSD as541be (2020)
Xtriage (Phenix)	:	1.13
EDS	:	2.16
Percentile statistics	:	20191225.v01 (using entries in the PDB archive December 25th 2019)
Refmac	:	5.8.0158
CCP4	:	7.0.044 (Gargrove)
Ideal geometry (proteins)	:	Engh & Huber (2001)
Ideal geometry (DNA, RNA)	:	Parkinson et al. (1996)
Validation Pipeline (wwPDB-VP)	:	2.16

CONFIDENTIAL VALIDATION REPORT

1 Overall quality at a glance (i)

The following experimental techniques were used to determine the structure:
X-RAY DIFFRACTION

The reported resolution of this entry is 1.80 Å.

Percentile scores (ranging between 0-100) for global validation metrics of the entry are shown in the following graphic. The table shows the number of entries on which the scores are based.

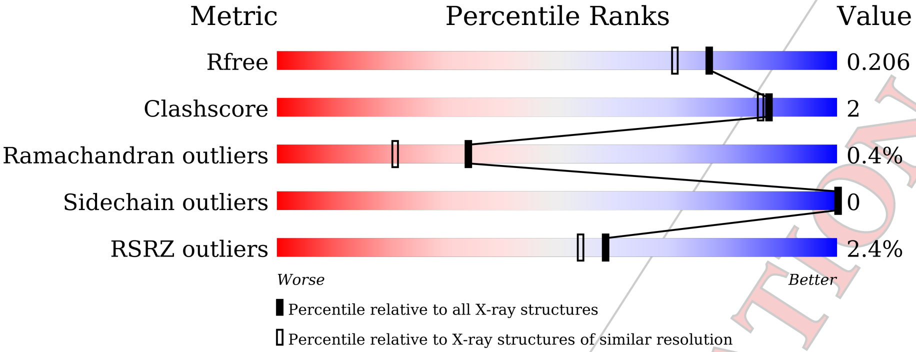

Metric	Whole archive (#Entries)	Similar resolution (#Entries, resolution range(Å))
R_{free}	130704	5950 (1.80-1.80)
Clashscore	141614	6793 (1.80-1.80)
Ramachandran outliers	138981	6697 (1.80-1.80)
Sidechain outliers	138945	6696 (1.80-1.80)
RSRZ outliers	127900	5850 (1.80-1.80)

The table below summarises the geometric issues observed across the polymeric chains and their fit to the electron density. The red, orange, yellow and green segments of the lower bar indicate the fraction of residues that contain outliers for >=3, 2, 1 and 0 types of geometric quality criteria respectively. A grey segment represents the fraction of residues that are not modelled. The numeric value for each fraction is indicated below the corresponding segment, with a dot representing fractions <=5% The upper red bar (where present) indicates the fraction of residues that have poor fit to the electron density. The numeric value is given above the bar.

Mol	Chain	Length	Quality of chain
1	A	540	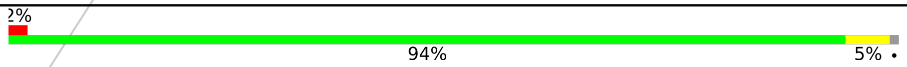
2	B	3	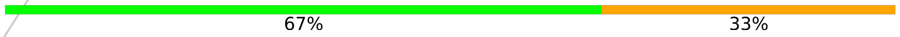

CONFIDENTIAL VALIDATION REPORT

2 Entry composition (i)

There are 7 unique types of molecules in this entry. The entry contains 4764 atoms, of which 0 are hydrogens and 0 are deuteriums.

In the tables below, the ZeroOcc column contains the number of atoms modelled with zero occupancy, the AltConf column contains the number of residues with at least one atom in alternate conformation and the Trace column contains the number of residues modelled with at most 2 atoms.

- Molecule 1 is a protein called Peptide ABC transporter, periplasmic peptide-binding protein.

Mol	Chain	Residues	Atoms					ZeroOcc	AltConf	Trace
			Total	C	N	O	S			
1	A	537	4301	2754	718	818	11	0	1	0

There are 8 discrepancies between the modelled and reference sequences:

Chain	Residue	Modelled	Actual	Comment	Reference
A	531	LEU	-	expression tag	UNP D0XC84
A	532	GLU	-	expression tag	UNP D0XC84
A	533	HIS	-	expression tag	UNP D0XC84
A	534	HIS	-	expression tag	UNP D0XC84
A	535	HIS	-	expression tag	UNP D0XC84
A	536	HIS	-	expression tag	UNP D0XC84
A	537	HIS	-	expression tag	UNP D0XC84
A	538	HIS	-	expression tag	UNP D0XC84

- Molecule 2 is an oligosaccharide called 2-acetamido-2-deoxy-beta-D-glucopyranose-(1-4)-2-acetamido-2-deoxy-beta-D-glucopyranose-(1-4)-2-acetamido-2-deoxy-alpha-D-glucopyranose.

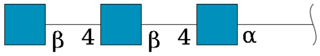

Mol	Chain	Residues	Atoms				ZeroOcc	AltConf	Trace
			Total	C	N	O			
2	B	3	86	48	6	32	0	3	0

- Molecule 3 is 1,2-ETHANEDIOL (three-letter code: EDO) (formula: $C_2H_6O_2$).

CONFIDENTIAL VALIDATION REPORT

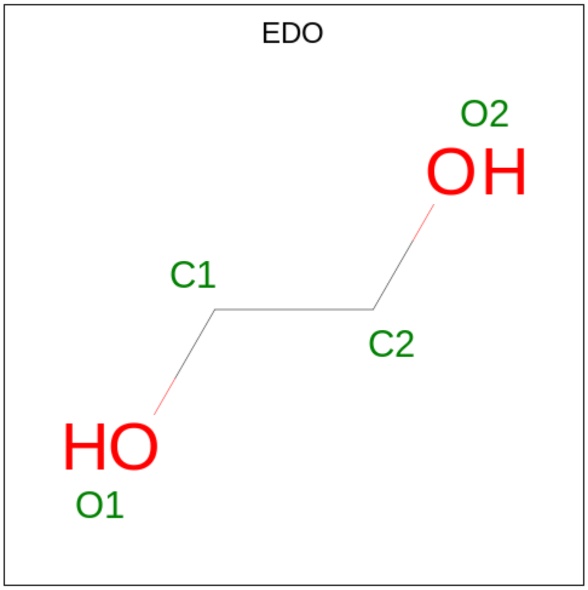

Mol	Chain	Residues	Atoms	ZeroOcc	AltConf
3	A	1	Total C O<br>4 2 2	0	0
3	A	1	Total C O<br>4 2 2	0	0
3	A	1	Total C O<br>4 2 2	0	0
3	A	1	Total C O<br>4 2 2	0	0
3	A	1	Total C O<br>4 2 2	0	0
3	A	1	Total C O<br>4 2 2	0	0
3	A	1	Total C O<br>4 2 2	0	0
3	A	1	Total C O<br>4 2 2	0	0

- Molecule 4 is MAGNESIUM ION (three-letter code: MG) (formula: Mg).

Mol	Chain	Residues	Atoms	ZeroOcc	AltConf
4	A	3	Total Mg<br>3 3	0	0

- Molecule 5 is CALCIUM ION (three-letter code: CA) (formula: Ca).

CONFIDENTIAL VALIDATION REPORT

Mol	Chain	Residues	Atoms	ZeroOcc	AltConf
5	A	2	Total Ca<br>2 2	0	0

- Molecule 6 is CHLORIDE ION (three-letter code: CL) (formula: Cl).

Mol	Chain	Residues	Atoms	ZeroOcc	AltConf
6	A	3	Total Cl<br>3 3	0	0

- Molecule 7 is water.

Mol	Chain	Residues	Atoms	ZeroOcc	AltConf
7	A	337	Total O<br>337 337	0	0

CONFIDENTIAL VALIDATION REPORT

3 Residue-property plots (i)

These plots are drawn for all protein, RNA, DNA and oligosaccharide chains in the entry. The first graphic for a chain summarises the proportions of the various outlier classes displayed in the second graphic. The second graphic shows the sequence view annotated by issues in geometry and electron density. Residues are color-coded according to the number of geometric quality criteria for which they contain at least one outlier: green = 0, yellow = 1, orange = 2 and red = 3 or more. A red dot above a residue indicates a poor fit to the electron density (RSRZ > 2). Stretches of 2 or more consecutive residues without any outlier are shown as a green connector. Residues present in the sample, but not in the model, are shown in grey.

- Molecule 1: Peptide ABC transporter, periplasmic peptide-binding protein

Chain A:

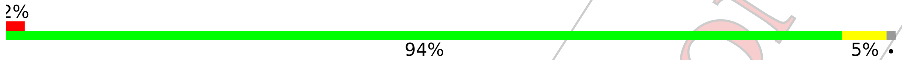

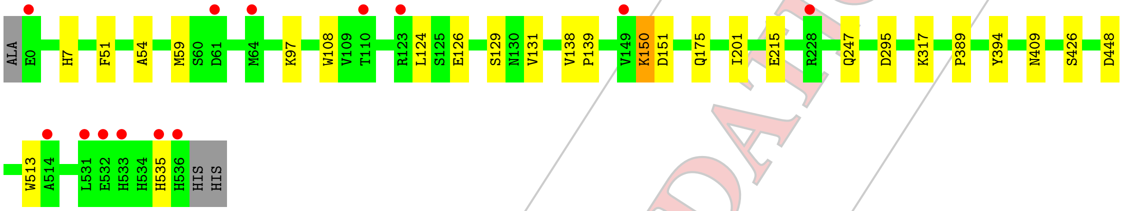

- Molecule 2: 2-acetamido-2-deoxy-beta-D-glucopyranose-(1-4)-2-acetamido-2-deoxy-beta-D-glucopyranose-(1-4)-2-acetamido-2-deoxy-alpha-D-glucopyranose

Chain B:

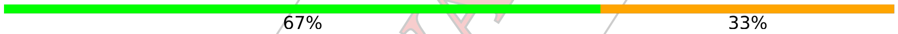

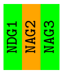

CONFIDENTIAL VALIDATION REPORT

4 Data and refinement statistics (i)

Property	Value	Source
Space group	P 1 21 1	Depositor
Cell constants<br>a, b, c, α, β, γ	61.83Å 56.84Å 81.86Å<br>90.00° 99.40° 90.00°	Depositor
Resolution (Å)	36.53 – 1.80<br>36.53 – 1.80	Depositor<br>EDS
% Data completeness<br>(in resolution range)	83.0 (36.53-1.80)<br>83.0 (36.53-1.80)	Depositor<br>EDS
R_{merge}	0.13	Depositor
R_{sym}	0.15	Depositor
$< I/\sigma(I) >$ [1]	2.32 (at 1.81Å)	Xtriage
Refinement program	PHENIX 1.9_1692	Depositor
R, R_{free}	0.164 , 0.206<br>0.166 , 0.206	Depositor<br>DCC
R_{free} test set	2123 reflections (4.90%)	wwPDB-VP
Wilson B-factor (Å^2)	20.4	Xtriage
Anisotropy	0.237	Xtriage
Bulk solvent k_{sol}(e/Å^3), B_{sol}(Å^2)	0.38 , 49.5	EDS
L-test for twinning[2]	$< \|L\| > = 0.49$, $< L^2 > = 0.32$	Xtriage
Estimated twinning fraction	No twinning to report.	Xtriage
F_o,F_c correlation	0.96	EDS
Total number of atoms	4764	wwPDB-VP
Average B, all atoms (Å^2)	24.0	wwPDB-VP

Xtriage's analysis on translational NCS is as follows: *The largest off-origin peak in the Patterson function is 5.27% of the height of the origin peak. No significant pseudotranslation is detected.*

[1] Intensities estimated from amplitudes.

[2] Theoretical values of $< |L| >$, $< L^2 >$ for acentric reflections are 0.5, 0.333 respectively for untwinned datasets, and 0.375, 0.2 for perfectly twinned datasets.

CONFIDENTIAL VALIDATION REPORT

5 Model quality (i)

5.1 Standard geometry (i)

Bond lengths and bond angles in the following residue types are not validated in this section: MG, NAG, CL, CA, EDO, NDG

The Z score for a bond length (or angle) is the number of standard deviations the observed value is removed from the expected value. A bond length (or angle) with $|Z| > 5$ is considered an outlier worth inspection. RMSZ is the root-mean-square of all Z scores of the bond lengths (or angles).

Mol	Chain	Bond lengths		Bond angles	
		RMSZ	$\#|Z|>5$	RMSZ	$\#|Z|>5$
1	A	0.38	0/4427	0.53	0/6033

There are no bond length outliers.

There are no bond angle outliers.

There are no chirality outliers.

There are no planarity outliers.

5.2 Too-close contacts (i)

In the following table, the Non-H and H(model) columns list the number of non-hydrogen atoms and hydrogen atoms in the chain respectively. The H(added) column lists the number of hydrogen atoms added and optimized by MolProbity. The Clashes column lists the number of clashes within the asymmetric unit, whereas Symm-Clashes lists symmetry-related clashes.

Mol	Chain	Non-H	H(model)	H(added)	Clashes	Symm-Clashes
1	A	4301	0	4069	16	0
2	B	86	0	72	1	0
3	A	32	0	47	2	0
4	A	3	0	0	0	0
5	A	2	0	0	0	0
6	A	3	0	0	0	0
7	A	337	0	0	2	0
All	All	4764	0	4188	17	0

The all-atom clashscore is defined as the number of clashes found per 1000 atoms (including hydrogen atoms). The all-atom clashscore for this structure is 2.

All (17) close contacts within the same asymmetric unit are listed below, sorted by their clash magnitude.

CONFIDENTIAL VALIDATION REPORT

Atom-1	Atom-2	Interatomic distance (Å)	Clash overlap (Å)
1:A:175:GLN:NE2	7:A:701:HOH:O	2.22	0.71
1:A:150:LYS:HD2	1:A:150:LYS:H	1.56	0.70
1:A:59:MET:HE3	1:A:129:SER:HA	1.89	0.54
1:A:215:GLU:HB3	3:A:608:EDO:H11	1.89	0.52
1:A:426:SER:OG	1:A:448:ASP:OD2	2.26	0.52
1:A:124:LEU:HD11	1:A:131:VAL:HG11	1.94	0.49
1:A:51:PHE:HB3	1:A:54:ALA:O	2.15	0.47
1:A:150:LYS:CD	1:A:150:LYS:H	2.21	0.45
1:A:295:ASP:OD2	1:A:317:LYS:HE3	2.18	0.43
1:A:389:PRO:HG2	1:A:394:TYR:HB2	2.01	0.42
1:A:97:LYS:NZ	1:A:151:ASP:OD1	2.49	0.42
1:A:247:GLN:HG2	1:A:409:ASN:HB3	2.01	0.42
1:A:7:HIS:CE1	1:A:201:ILE:HB	2.54	0.42
1:A:108:TRP:HZ3	1:A:126:GLU:HB3	1.84	0.42
3:A:601:EDO:H12	7:A:732:HOH:O	2.19	0.42
1:A:513:TRP:CD1	2:B:2[B]:NAG:H5	2.55	0.41
1:A:138:VAL:HA	1:A:139:PRO:HD3	1.95	0.41

There are no symmetry-related clashes.

5.3 Torsion angles (i)

5.3.1 Protein backbone (i)

In the following table, the Percentiles column shows the percent Ramachandran outliers of the chain as a percentile score with respect to all X-ray entries followed by that with respect to entries of similar resolution.

The Analysed column shows the number of residues for which the backbone conformation was analysed, and the total number of residues.

Mol	Chain	Analysed	Favoured	Allowed	Outliers	Percentiles
1	A	536/540 (99%)	517 (96%)	17 (3%)	2 (0%)	34 21

All (2) Ramachandran outliers are listed below:

Mol	Chain	Res	Type
1	A	150	LYS
1	A	535	HIS

CONFIDENTIAL VALIDATION REPORT

5.3.2 Protein sidechains (i)

In the following table, the Percentiles column shows the percent sidechain outliers of the chain as a percentile score with respect to all X-ray entries followed by that with respect to entries of similar resolution.

The Analysed column shows the number of residues for which the sidechain conformation was analysed, and the total number of residues.

Mol	Chain	Analysed	Rotameric	Outliers	Percentiles	
1	A	459/464 (99%)	459 (100%)	0	100	100

There are no protein residues with a non-rotameric sidechain to report.

Sometimes sidechains can be flipped to improve hydrogen bonding and reduce clashes. There are no such sidechains identified.

5.3.3 RNA (i)

There are no RNA molecules in this entry.

5.4 Non-standard residues in protein, DNA, RNA chains (i)

There are no non-standard protein/DNA/RNA residues in this entry.

5.5 Carbohydrates (i)

6 monosaccharides are modelled in this entry.

In the following table, the Counts columns list the number of bonds (or angles) for which Mogul statistics could be retrieved, the number of bonds (or angles) that are observed in the model and the number of bonds (or angles) that are defined in the Chemical Component Dictionary. The Link column lists molecule types, if any, to which the group is linked. The Z score for a bond length (or angle) is the number of standard deviations the observed value is removed from the expected value. A bond length (or angle) with $|Z| > 2$ is considered an outlier worth inspection. RMSZ is the root-mean-square of all Z scores of the bond lengths (or angles).

Mol	Type	Chain	Res	Link	Bond lengths			Bond angles		
					Counts	RMSZ	$\#\|Z\| > 2$	Counts	RMSZ	$\#\|Z\| > 2$
2	NDG	B	1[A]	2	15,15,15	0.15	0	21,21,21	0.47	0
2	NDG	B	1[B]	2	15,15,15	0.14	0	21,21,21	0.39	0
2	NAG	B	2[A]	2	14,14,15	0.40	0	17,19,21	0.93	1 (5%)
2	NAG	B	2[B]	2	14,14,15	0.40	0	17,19,21	0.78	1 (5%)
2	NAG	B	3[A]	2	14,14,15	0.39	0	17,19,21	0.66	0

CONFIDENTIAL VALIDATION REPORT

Mol	Type	Chain	Res	Link	Bond lengths			Bond angles		
					Counts	RMSZ	#|Z| > 2	Counts	RMSZ	#|Z| > 2
2	NAG	B	3[B]	2	14,14,15	0.36	0	17,19,21	0.26	0

In the following table, the Chirals column lists the number of chiral outliers, the number of chiral centers analysed, the number of these observed in the model and the number defined in the Chemical Component Dictionary. Similar counts are reported in the Torsion and Rings columns. '-' means no outliers of that kind were identified.

Mol	Type	Chain	Res	Link	Chirals	Torsions	Rings
2	NDG	B	1[A]	2	-	0/6/26/26	0/1/1/1
2	NDG	B	1[B]	2	-	0/6/26/26	0/1/1/1
2	NAG	B	2[A]	2	-	0/6/23/26	0/1/1/1
2	NAG	B	2[B]	2	-	0/6/23/26	0/1/1/1
2	NAG	B	3[A]	2	-	0/6/23/26	0/1/1/1
2	NAG	B	3[B]	2	-	0/6/23/26	0/1/1/1

There are no bond length outliers.

All (2) bond angle outliers are listed below:

Mol	Chain	Res	Type	Atoms	Z	Observed(^{o})	Ideal(^{o})
2	B	2[A]	NAG	C4-C3-C2	-3.16	106.38	111.02
2	B	2[B]	NAG	C4-C3-C2	-2.73	107.01	111.02

There are no chirality outliers.

There are no torsion outliers.

There are no ring outliers.

1 monomer is involved in 1 short contact:

Mol	Chain	Res	Type	Clashes	Symm-Clashes
2	B	2[B]	NAG	1	0

The following is a two-dimensional graphical depiction of Mogul quality analysis of bond lengths, bond angles, torsion angles, and ring geometry for oligosaccharide.

CONFIDENTIAL VALIDATION REPORT

Oligosaccharide Chain B

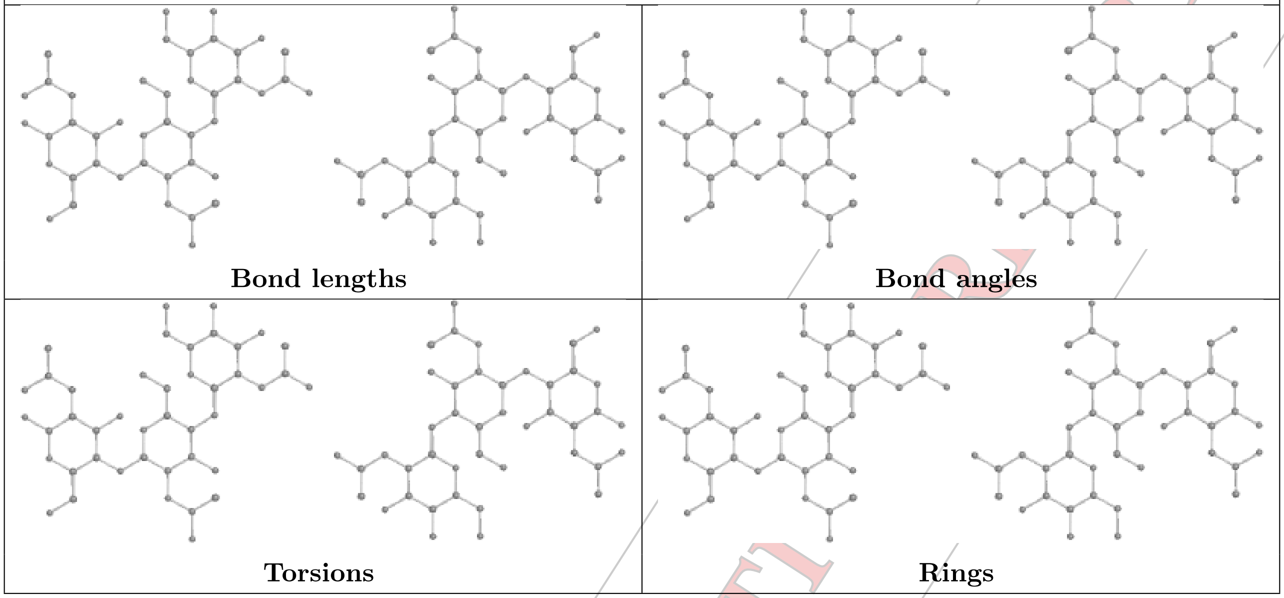

5.6 Ligand geometry (i)

Of 16 ligands modelled in this entry, 8 are monoatomic - leaving 8 for Mogul analysis.

In the following table, the Counts columns list the number of bonds (or angles) for which Mogul statistics could be retrieved, the number of bonds (or angles) that are observed in the model and the number of bonds (or angles) that are defined in the Chemical Component Dictionary. The Link column lists molecule types, if any, to which the group is linked. The Z score for a bond length (or angle) is the number of standard deviations the observed value is removed from the expected value. A bond length (or angle) with $|Z| > 2$ is considered an outlier worth inspection. RMSZ is the root-mean-square of all Z scores of the bond lengths (or angles).

Mol	Type	Chain	Res	Link	Bond lengths			Bond angles		
					Counts	RMSZ	$\#\|Z\| > 2$	Counts	RMSZ	$\#\|Z\| > 2$
3	EDO	A	602	-	3,3,3	0.42	0	2,2,2	0.53	0
3	EDO	A	607	-	3,3,3	0.46	0	2,2,2	0.65	0
3	EDO	A	603	4	3,3,3	0.46	0	2,2,2	0.32	0
3	EDO	A	604	-	3,3,3	0.46	0	2,2,2	0.25	0
3	EDO	A	605	-	3,3,3	0.50	0	2,2,2	0.25	0
3	EDO	A	601	-	3,3,3	0.47	0	2,2,2	0.39	0
3	EDO	A	608	-	3,3,3	0.48	0	2,2,2	0.44	0
3	EDO	A	606	4	3,3,3	0.41	0	2,2,2	0.48	0

In the following table, the Chirals column lists the number of chiral outliers, the number of chiral centers analysed, the number of these observed in the model and the number defined in the Chemical Component Dictionary. Similar counts are reported in the Torsion and Rings columns. '-' means no outliers of that kind were identified.

CONFIDENTIAL VALIDATION REPORT

Mol	Type	Chain	Res	Link	Chirals	Torsions	Rings
3	EDO	A	602	-	-	0/1/1/1	-
3	EDO	A	607	-	-	0/1/1/1	-
3	EDO	A	603	4	-	1/1/1/1	-
3	EDO	A	604	-	-	0/1/1/1	-
3	EDO	A	605	-	-	0/1/1/1	-
3	EDO	A	601	-	-	0/1/1/1	-
3	EDO	A	608	-	-	0/1/1/1	-
3	EDO	A	606	4	-	0/1/1/1	-

There are no bond length outliers.

There are no bond angle outliers.

There are no chirality outliers.

All (1) torsion outliers are listed below:

Mol	Chain	Res	Type	Atoms
3	A	603	EDO	O1-C1-C2-O2

There are no ring outliers.

2 monomers are involved in 2 short contacts:

Mol	Chain	Res	Type	Clashes	Symm-Clashes
3	A	601	EDO	1	0
3	A	608	EDO	1	0

5.7 Other polymers (i)

There are no such residues in this entry.

5.8 Polymer linkage issues (i)

There are no chain breaks in this entry.

CONFIDENTIAL VALIDATION REPORT

6 Fit of model and data (i)

6.1 Protein, DNA and RNA chains (i)

In the following table, the column labelled '#RSRZ> 2' contains the number (and percentage) of RSRZ outliers, followed by percent RSRZ outliers for the chain as percentile scores relative to all X-ray entries and entries of similar resolution. The OWAB column contains the minimum, median, 95th percentile and maximum values of the occupancy-weighted average B-factor per residue. The column labelled 'Q< 0.9' lists the number of (and percentage) of residues with an average occupancy less than 0.9.

Mol	Chain	Analysed	<RSRZ>	#RSRZ>2			OWAB(Å^2)	Q<0.9
1	A	537/540 (99%)	-0.26	13 (2%)	59	54	12, 22, 40, 57	0

All (13) RSRZ outliers are listed below:

Mol	Chain	Res	Type	RSRZ
1	A	531	LEU	5.1
1	A	536	HIS	4.7
1	A	61	ASP	3.3
1	A	535	HIS	2.6
1	A	64	MET	2.5
1	A	0	GLU	2.4
1	A	149	VAL	2.4
1	A	532	GLU	2.3
1	A	514	ALA	2.3
1	A	110	THR	2.2
1	A	123	ARG	2.2
1	A	533	HIS	2.1
1	A	228	ARG	2.0

6.2 Non-standard residues in protein, DNA, RNA chains (i)

There are no non-standard protein/DNA/RNA residues in this entry.

6.3 Carbohydrates (i)

In the following table, the Atoms column lists the number of modelled atoms in the group and the number defined in the chemical component dictionary. The B-factors column lists the minimum, median, 95th percentile and maximum values of B factors of atoms in the group. The column labelled 'Q< 0.9' lists the number of atoms with occupancy less than 0.9.

CONFIDENTIAL VALIDATION REPORT

Mol	Type	Chain	Res	Atoms	RSCC	RSR	B-factors($Å^2$)	Q<0.9
2	NDG	B	1[B]	15/15	0.95	0.14	17,24,28,29	15
2	NDG	B	1[A]	15/15	0.95	0.14	17,24,29,29	15
2	NAG	B	2[A]	14/15	0.97	0.16	14,16,17,17	14
2	NAG	B	2[B]	14/15	0.97	0.16	14,16,17,17	14
2	NAG	B	3[A]	14/15	0.98	0.13	12,14,15,16	14
2	NAG	B	3[B]	14/15	0.98	0.13	13,14,15,16	14

The following is a graphical depiction of the model fit to experimental electron density for oligosaccharide. Each fit is shown from different orientation to approximate a three-dimensional view.

Electron density around Chain B:

2mF$_o$-DF$_c$ (at 0.7 rmsd) in gray
mF$_o$-DF$_c$ (at 3 rmsd) in purple (negative)
and green (positive)

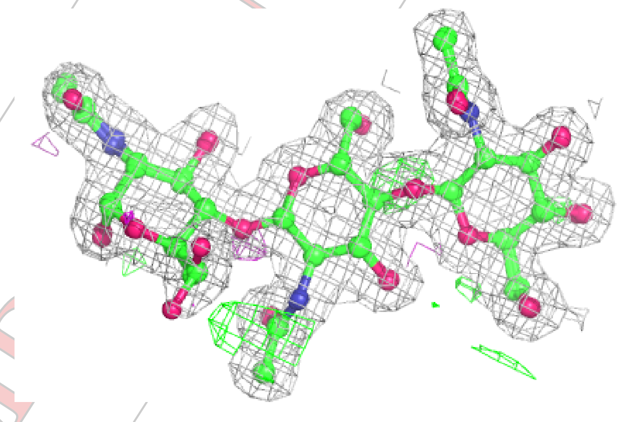

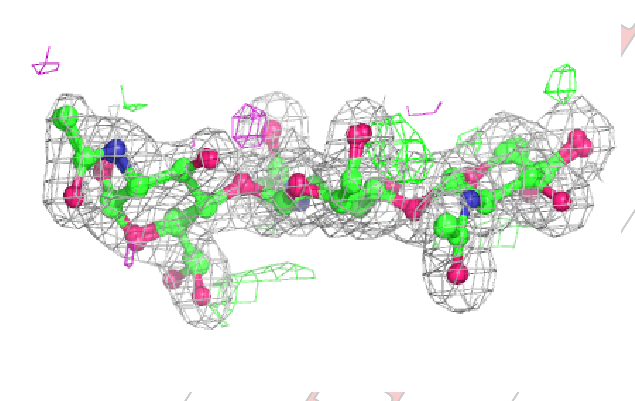

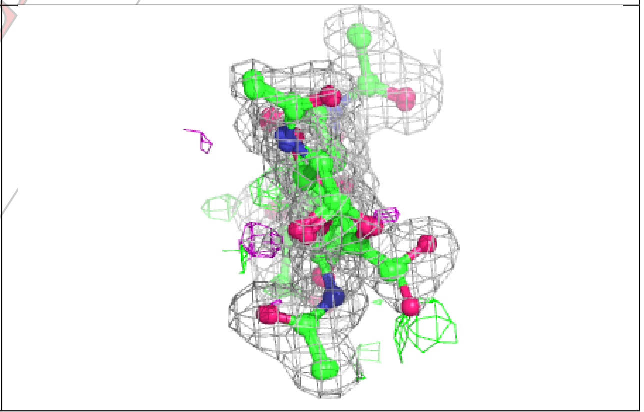

6.4 Ligands ⓘ

In the following table, the Atoms column lists the number of modelled atoms in the group and the number defined in the chemical component dictionary. The B-factors column lists the minimum, median, 95th percentile and maximum values of B factors of atoms in the group. The column labelled 'Q< 0.9' lists the number of atoms with occupancy less than 0.9.

Mol	Type	Chain	Res	Atoms	RSCC	RSR	B-factors($Å^2$)	Q<0.9
3	EDO	A	607	4/4	0.87	0.12	31,32,39,42	0
3	EDO	A	605	4/4	0.89	0.12	36,41,41,43	0
6	CL	A	615	1/1	0.90	0.08	51,51,51,51	0

Continued on next page...

CONFIDENTIAL REPORT

Continued from previous page...

Mol	Type	Chain	Res	Atoms	RSCC	RSR	B-factors(Å^2)	Q<0.9
3	EDO	A	602	4/4	0.92	0.12	37,39,39,40	0
3	EDO	A	606	4/4	0.93	0.23	32,36,41,46	0
3	EDO	A	603	4/4	0.94	0.08	29,30,31,33	0
3	EDO	A	601	4/4	0.94	0.11	24,25,29,34	0
3	EDO	A	608	4/4	0.94	0.08	29,33,33,35	0
5	CA	A	612	1/1	0.94	0.06	51,51,51,51	0
4	MG	A	611	1/1	0.97	0.05	22,22,22,22	0
3	EDO	A	604	4/4	0.97	0.06	26,30,30,37	0
6	CL	A	616	1/1	0.98	0.17	42,42,42,42	0
4	MG	A	610	1/1	0.98	0.05	20,20,20,20	0
4	MG	A	609	1/1	0.99	0.09	11,11,11,11	0
6	CL	A	614	1/1	1.00	0.06	18,18,18,18	0
5	CA	A	613	1/1	1.00	0.04	18,18,18,18	0

6.5 Other polymers (i)

There are no such residues in this entry.

CONFIDENTIAL VALIDATION REPORT

Full wwPDB X-ray Structure Validation Report (i)

Mar 16, 2021 – 04:13 PM JST

PDB ID	:	7EBI
Title	:	Chitin-specific solute binding protein from Vibrio harveyi co-crystalized with chitotetraose.
Deposited on	:	2021-03-09
Resolution	:	1.50 Å(reported)

This is a Full wwPDB X-ray Structure Validation Report.

This report is produced by the wwPDB biocuration pipeline after annotation of the structure.

We welcome your comments at *validation@mail.wwpdb.org*
A user guide is available at
https://www.wwpdb.org/validation/2017/XrayValidationReportHelp
with specific help available everywhere you see the (i) symbol.

CONFIDENTIAL VALIDATION REPORT

The following versions of software and data (see references (i)) were used in the production of this report:

MolProbity	:	4.02b-467
Mogul	:	1.8.5 (274361), CSD as541be (2020)
Xtriage (Phenix)	:	1.13
EDS	:	2.17.1
Percentile statistics	:	20191225.v01 (using entries in the PDB archive December 25th 2019)
Refmac	:	5.8.0158
CCP4	:	7.0.044 (Gargrove)
Ideal geometry (proteins)	:	Engh & Huber (2001)
Ideal geometry (DNA, RNA)	:	Parkinson et al. (1996)
Validation Pipeline (wwPDB-VP)	:	2.17.1

1 Overall quality at a glance (i)

The following experimental techniques were used to determine the structure:
X-RAY DIFFRACTION

The reported resolution of this entry is 1.50 Å.

Percentile scores (ranging between 0-100) for global validation metrics of the entry are shown in the following graphic. The table shows the number of entries on which the scores are based.

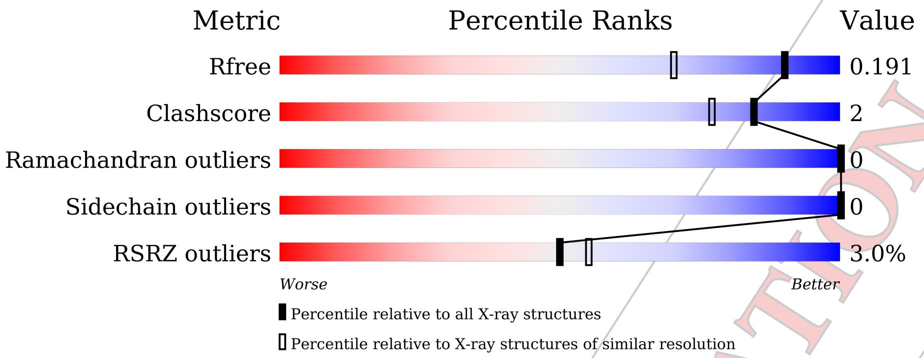

Metric	Whole archive (#Entries)	Similar resolution (#Entries, resolution range(Å))
R_{free}	130704	2936 (1.50-1.50)
Clashscore	141614	3144 (1.50-1.50)
Ramachandran outliers	138981	3066 (1.50-1.50)
Sidechain outliers	138945	3064 (1.50-1.50)
RSRZ outliers	127900	2884 (1.50-1.50)

The table below summarises the geometric issues observed across the polymeric chains and their fit to the electron density. The red, orange, yellow and green segments of the lower bar indicate the fraction of residues that contain outliers for >=3, 2, 1 and 0 types of geometric quality criteria respectively. A grey segment represents the fraction of residues that are not modelled. The numeric value for each fraction is indicated below the corresponding segment, with a dot representing fractions <=5% The upper red bar (where present) indicates the fraction of residues that have poor fit to the electron density. The numeric value is given above the bar.

Mol	Chain	Length	Quality of chain
1	A	540	3%<br>95% • •
2	E	2	50% 50%

The following table lists non-polymeric compounds, carbohydrate monomers and non-standard residues in protein, DNA, RNA chains that are outliers for geometric or electron-density-fit criteria:

CONFIDENTIAL VALIDATION REPORT

Mol	Type	Chain	Res	Chirality	Geometry	Clashes	Electron density
3	EDO	A	601	-	-	X	-

CONFIDENTIAL VALIDATION REPORT

2 Entry composition (i)

There are 7 unique types of molecules in this entry. The entry contains 4735 atoms, of which 0 are hydrogens and 0 are deuteriums.

In the tables below, the ZeroOcc column contains the number of atoms modelled with zero occupancy, the AltConf column contains the number of residues with at least one atom in alternate conformation and the Trace column contains the number of residues modelled with at most 2 atoms.

- Molecule 1 is a protein called Peptide ABC transporter, periplasmic peptide-binding protein.

Mol	Chain	Residues	Atoms					ZeroOcc	AltConf	Trace
			Total	C	N	O	S			
1	A	537	4338	2778	719	828	13	0	8	0

There are 8 discrepancies between the modelled and reference sequences:

Chain	Residue	Modelled	Actual	Comment	Reference
A	531	LEU	-	expression tag	UNP D0XC84
A	532	GLU	-	expression tag	UNP D0XC84
A	533	HIS	-	expression tag	UNP D0XC84
A	534	HIS	-	expression tag	UNP D0XC84
A	535	HIS	-	expression tag	UNP D0XC84
A	536	HIS	-	expression tag	UNP D0XC84
A	537	HIS	-	expression tag	UNP D0XC84
A	538	HIS	-	expression tag	UNP D0XC84

- Molecule 2 is an oligosaccharide called 2-acetamido-2-deoxy-beta-D-glucopyranose-(1-4)-2-acetamido-2-deoxy-beta-D-glucopyranose.

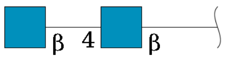

Mol	Chain	Residues	Atoms				ZeroOcc	AltConf	Trace
			Total	C	N	O			
2	E	2	29	16	2	11	0	0	0

- Molecule 3 is 1,2-ETHANEDIOL (three-letter code: EDO) (formula: $C_2H_6O_2$).

CONFIDENTIAL VALIDATION REPORT

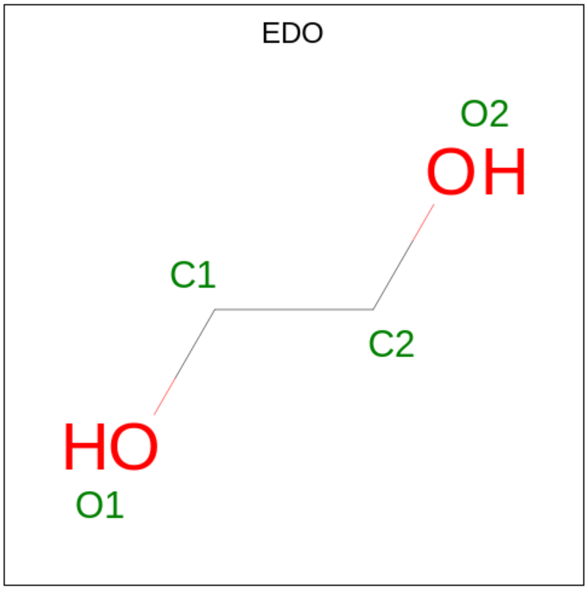

Mol	Chain	Residues	Atoms	ZeroOcc	AltConf
3	A	1	Total C O<br>4 2 2	0	0
3	A	1	Total C O<br>4 2 2	0	0
3	A	1	Total C O<br>4 2 2	0	0
3	A	1	Total C O<br>4 2 2	0	0
3	A	1	Total C O<br>4 2 2	0	0
3	A	1	Total C O<br>4 2 2	0	0
3	A	1	Total C O<br>4 2 2	0	0
3	A	1	Total C O<br>4 2 2	0	0
3	A	1	Total C O<br>4 2 2	0	0
3	A	1	Total C O<br>4 2 2	0	0
3	A	1	Total C O<br>4 2 2	0	0
3	A	1	Total C O<br>4 2 2	0	0
3	A	1	Total C O<br>4 2 2	0	0

- Molecule 4 is MAGNESIUM ION (three-letter code: MG) (formula: Mg).

CONFIDENTIAL VALIDATION REPORT

Mol	Chain	Residues	Atoms		ZeroOcc	AltConf
			Total	Mg		
4	A	4	4	4	0	0

- Molecule 5 is CALCIUM ION (three-letter code: CA) (formula: Ca).

Mol	Chain	Residues	Atoms		ZeroOcc	AltConf
			Total	Ca		
5	A	2	2	2	0	0

- Molecule 6 is CHLORIDE ION (three-letter code: CL) (formula: Cl).

Mol	Chain	Residues	Atoms		ZeroOcc	AltConf
			Total	Cl		
6	A	2	2	2	0	0

- Molecule 7 is water.

Mol	Chain	Residues	Atoms		ZeroOcc	AltConf
			Total	O		
7	A	308	308	308	0	0

CONFIDENTIAL VALIDATION REPORT

3 Residue-property plots (i)

These plots are drawn for all protein, RNA, DNA and oligosaccharide chains in the entry. The first graphic for a chain summarises the proportions of the various outlier classes displayed in the second graphic. The second graphic shows the sequence view annotated by issues in geometry and electron density. Residues are color-coded according to the number of geometric quality criteria for which they contain at least one outlier: green = 0, yellow = 1, orange = 2 and red = 3 or more. A red dot above a residue indicates a poor fit to the electron density (RSRZ > 2). Stretches of 2 or more consecutive residues without any outlier are shown as a green connector. Residues present in the sample, but not in the model, are shown in grey.

• Molecule 1: Peptide ABC transporter, periplasmic peptide-binding protein

Chain A:

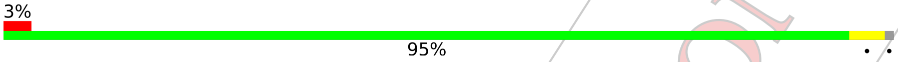

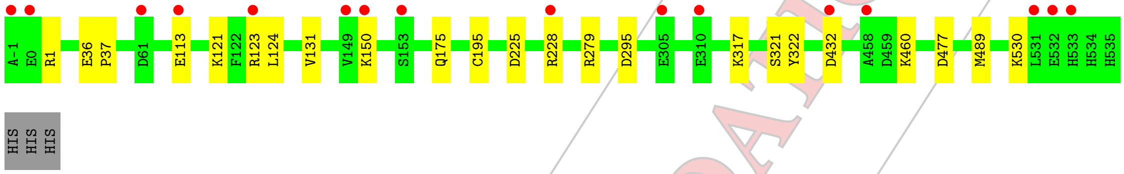

• Molecule 2: 2-acetamido-2-deoxy-beta-D-glucopyranose-(1-4)-2-acetamido-2-deoxy-beta-D-gluc opyranose

Chain E:

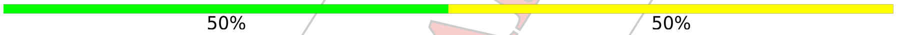

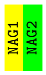

CONFIDENTIAL VALIDATION REPORT

4 Data and refinement statistics (i)

Property	Value	Source
Space group	P 1 21 1	Depositor
Cell constants<br>a, b, c, α, β, γ	61.88Å 57.11Å 82.13Å<br>90.00° 99.54° 90.00°	Depositor
Resolution (Å)	24.49 – 1.50<br>24.49 – 1.50	Depositor<br>EDS
% Data completeness<br>(in resolution range)	95.5 (24.49-1.50)<br>95.5 (24.49-1.50)	Depositor<br>EDS
R_{merge}	(Not available)	Depositor
R_{sym}	(Not available)	Depositor
$< I/\sigma(I) >$ [1]	3.07 (at 1.49Å)	Xtriage
Refinement program	PHENIX 1.9_1692	Depositor
R, R_{free}	0.174 , 0.192<br>0.174 , 0.191	Depositor<br>DCC
R_{free} test set	4261 reflections (4.89%)	wwPDB-VP
Wilson B-factor (Å^2)	13.0	Xtriage
Anisotropy	0.050	Xtriage
Bulk solvent k_{sol}(e/Å^3), B_{sol}(Å^2)	0.41 , 54.7	EDS
L-test for twinning[2]	$< \|L\| > = 0.47$, $< L^2 > = 0.30$	Xtriage
Estimated twinning fraction	No twinning to report.	Xtriage
F_o,F_c correlation	0.96	EDS
Total number of atoms	4735	wwPDB-VP
Average B, all atoms (Å^2)	16.0	wwPDB-VP

Xtriage's analysis on translational NCS is as follows: *The largest off-origin peak in the Patterson function is 5.13% of the height of the origin peak. No significant pseudotranslation is detected.*

[1] Intensities estimated from amplitudes.

[2] Theoretical values of $< |L| >$, $< L^2 >$ for acentric reflections are 0.5, 0.333 respectively for untwinned datasets, and 0.375, 0.2 for perfectly twinned datasets.

CONFIDENTIAL VALIDATION REPORT

5 Model quality (i)

5.1 Standard geometry (i)

Bond lengths and bond angles in the following residue types are not validated in this section: NAG, MG, CL, EDO, CA

The Z score for a bond length (or angle) is the number of standard deviations the observed value is removed from the expected value. A bond length (or angle) with $|Z| > 5$ is considered an outlier worth inspection. RMSZ is the root-mean-square of all Z scores of the bond lengths (or angles).

Mol	Chain	Bond lengths		Bond angles	
		RMSZ	$\#|Z|>5$	RMSZ	$\#|Z|>5$
1	A	0.35	0/4474	0.52	0/6093

There are no bond length outliers.

There are no bond angle outliers.

There are no chirality outliers.

There are no planarity outliers.

5.2 Too-close contacts (i)

In the following table, the Non-H and H(model) columns list the number of non-hydrogen atoms and hydrogen atoms in the chain respectively. The H(added) column lists the number of hydrogen atoms added and optimized by MolProbity. The Clashes column lists the number of clashes within the asymmetric unit, whereas Symm-Clashes lists symmetry-related clashes.

Mol	Chain	Non-H	H(model)	H(added)	Clashes	Symm-Clashes
1	A	4338	0	4126	20	0
2	E	29	0	27	0	0
3	A	52	0	77	6	0
4	A	4	0	0	0	0
5	A	2	0	0	0	0
6	A	2	0	0	0	0
7	A	308	0	0	5	0
All	All	4735	0	4230	20	0

The all-atom clashscore is defined as the number of clashes found per 1000 atoms (including hydrogen atoms). The all-atom clashscore for this structure is 2.

All (20) close contacts within the same asymmetric unit are listed below, sorted by their clash magnitude.

CONFIDENTIAL VALIDATION REPORT

Atom-1	Atom-2	Interatomic distance (Å)	Clash overlap (Å)
1:A:113:GLU:OE1	1:A:123:ARG:NH1	2.14	0.80
1:A:322:TYR:HB2	3:A:601:EDO:H21	1.69	0.72
1:A:279:ARG:H	3:A:601:EDO:H22	1.58	0.69
1:A:113:GLU:HG2	1:A:121:LYS:HB3	1.76	0.66
1:A:150:LYS:HD3	1:A:150:LYS:N	2.13	0.62
1:A:225:ASP:OD2	1:A:228:ARG:NE	2.25	0.60
1:A:489[A]:MET:SD	7:A:845:HOH:O	2.57	0.60
1:A:321:SER:HA	3:A:601:EDO:H12	1.84	0.59
1:A:279:ARG:H	3:A:601:EDO:C2	2.19	0.56
1:A:432:ASP:O	7:A:701:HOH:O	2.20	0.49
1:A:124:LEU:HD11	1:A:131:VAL:HG11	1.95	0.48
1:A:530[B]:LYS:HG3	7:A:706:HOH:O	2.14	0.46
1:A:295:ASP:OD2	1:A:317:LYS:HE3	2.17	0.45
1:A:36:GLU:HG3	1:A:37:PRO:HD2	1.99	0.44
1:A:175:GLN:NE2	7:A:704:HOH:O	2.39	0.43
1:A:460:LYS:HE2	7:A:968:HOH:O	2.18	0.43
1:A:477:ASP:OD1	3:A:610:EDO:O2	2.32	0.41
1:A:530[B]:LYS:HD2	1:A:530[B]:LYS:O	2.20	0.41
1:A:279:ARG:N	3:A:601:EDO:H22	2.29	0.41
1:A:1:ARG:HD2	1:A:195[B]:CYS:SG	2.61	0.40

There are no symmetry-related clashes.

5.3 Torsion angles (i)

5.3.1 Protein backbone (i)

In the following table, the Percentiles column shows the percent Ramachandran outliers of the chain as a percentile score with respect to all X-ray entries followed by that with respect to entries of similar resolution.

The Analysed column shows the number of residues for which the backbone conformation was analysed, and the total number of residues.

Mol	Chain	Analysed	Favoured	Allowed	Outliers	Percentiles	
1	A	543/540 (101%)	531 (98%)	12 (2%)	0	100	100

There are no Ramachandran outliers to report.

CONFIDENTIAL VALIDATION REPORT

5.3.2 Protein sidechains (i)

In the following table, the Percentiles column shows the percent sidechain outliers of the chain as a percentile score with respect to all X-ray entries followed by that with respect to entries of similar resolution.

The Analysed column shows the number of residues for which the sidechain conformation was analysed, and the total number of residues.

Mol	Chain	Analysed	Rotameric	Outliers	Percentiles	
1	A	466/464 (100%)	466 (100%)	0	100	100

There are no protein residues with a non-rotameric sidechain to report.

Sometimes sidechains can be flipped to improve hydrogen bonding and reduce clashes. There are no such sidechains identified.

5.3.3 RNA (i)

There are no RNA molecules in this entry.

5.4 Non-standard residues in protein, DNA, RNA chains (i)

There are no non-standard protein/DNA/RNA residues in this entry.

5.5 Carbohydrates (i)

2 monosaccharides are modelled in this entry.

In the following table, the Counts columns list the number of bonds (or angles) for which Mogul statistics could be retrieved, the number of bonds (or angles) that are observed in the model and the number of bonds (or angles) that are defined in the Chemical Component Dictionary. The Link column lists molecule types, if any, to which the group is linked. The Z score for a bond length (or angle) is the number of standard deviations the observed value is removed from the expected value. A bond length (or angle) with $|Z| > 2$ is considered an outlier worth inspection. RMSZ is the root-mean-square of all Z scores of the bond lengths (or angles).

Mol	Type	Chain	Res	Link	Bond lengths			Bond angles		
					Counts	RMSZ	$\#\|Z\| > 2$	Counts	RMSZ	$\#\|Z\| > 2$
2	NAG	E	1	2	15,15,15	0.43	0	21,21,21	1.01	2 (9%)
2	NAG	E	2	2	14,14,15	0.31	0	17,19,21	0.81	0

In the following table, the Chirals column lists the number of chiral outliers, the number of chiral centers analysed, the number of these observed in the model and the number defined in the

CONFIDENTIAL VALIDATION REPORT

Chemical Component Dictionary. Similar counts are reported in the Torsion and Rings columns. '-' means no outliers of that kind were identified.

Mol	Type	Chain	Res	Link	Chirals	Torsions	Rings
2	NAG	E	1	2	-	0/6/26/26	0/1/1/1
2	NAG	E	2	2	-	0/6/23/26	0/1/1/1

There are no bond length outliers.

All (2) bond angle outliers are listed below:

Mol	Chain	Res	Type	Atoms	Z	Observed(^{o})	Ideal(^{o})
2	E	1	NAG	C1-C2-C3	-2.60	107.00	110.54
2	E	1	NAG	C1-O5-C5	-2.06	109.77	113.66

There are no chirality outliers.

There are no torsion outliers.

There are no ring outliers.

No monomer is involved in short contacts.

5.6 Ligand geometry (i)

Of 21 ligands modelled in this entry, 8 are monoatomic - leaving 13 for Mogul analysis.

In the following table, the Counts columns list the number of bonds (or angles) for which Mogul statistics could be retrieved, the number of bonds (or angles) that are observed in the model and the number of bonds (or angles) that are defined in the Chemical Component Dictionary. The Link column lists molecule types, if any, to which the group is linked. The Z score for a bond length (or angle) is the number of standard deviations the observed value is removed from the expected value. A bond length (or angle) with $|Z| > 2$ is considered an outlier worth inspection. RMSZ is the root-mean-square of all Z scores of the bond lengths (or angles).

Mol	Type	Chain	Res	Link	Bond lengths			Bond angles		
					Counts	RMSZ	$\#\|Z\| > 2$	Counts	RMSZ	$\#\|Z\| > 2$
3	EDO	A	606	-	3,3,3	0.49	0	2,2,2	0.34	0
3	EDO	A	607	-	3,3,3	0.41	0	2,2,2	0.07	0
3	EDO	A	601	-	3,3,3	0.24	0	2,2,2	1.04	0
3	EDO	A	613	4	3,3,3	0.44	0	2,2,2	0.38	0
3	EDO	A	611	4	3,3,3	0.48	0	2,2,2	0.40	0
3	EDO	A	608	-	3,3,3	0.44	0	2,2,2	0.43	0
3	EDO	A	612	-	3,3,3	0.47	0	2,2,2	0.46	0
3	EDO	A	609	-	3,3,3	0.44	0	2,2,2	0.41	0
3	EDO	A	605	-	3,3,3	0.48	0	2,2,2	0.31	0
3	EDO	A	610	-	3,3,3	0.45	0	2,2,2	0.56	0

CONFIDENTIAL VALIDATION REPORT

Mol	Type	Chain	Res	Link	Bond lengths			Bond angles		
					Counts	RMSZ	#|Z| > 2	Counts	RMSZ	#|Z| > 2
3	EDO	A	602	-	3,3,3	0.42	0	2,2,2	0.40	0
3	EDO	A	603	4	3,3,3	0.47	0	2,2,2	0.34	0
3	EDO	A	604	-	3,3,3	0.44	0	2,2,2	0.34	0

In the following table, the Chirals column lists the number of chiral outliers, the number of chiral centers analysed, the number of these observed in the model and the number defined in the Chemical Component Dictionary. Similar counts are reported in the Torsion and Rings columns. '-' means no outliers of that kind were identified.

Mol	Type	Chain	Res	Link	Chirals	Torsions	Rings
3	EDO	A	606	-	-	0/1/1/1	-
3	EDO	A	607	-	-	1/1/1/1	-
3	EDO	A	601	-	-	1/1/1/1	-
3	EDO	A	613	4	-	0/1/1/1	-
3	EDO	A	611	4	-	0/1/1/1	-
3	EDO	A	608	-	-	0/1/1/1	-
3	EDO	A	612	-	-	0/1/1/1	-
3	EDO	A	609	-	-	0/1/1/1	-
3	EDO	A	605	-	-	0/1/1/1	-
3	EDO	A	610	-	-	0/1/1/1	-
3	EDO	A	602	-	-	0/1/1/1	-
3	EDO	A	603	4	-	0/1/1/1	-
3	EDO	A	604	-	-	0/1/1/1	-

There are no bond length outliers.

There are no bond angle outliers.

There are no chirality outliers.

All (2) torsion outliers are listed below:

Mol	Chain	Res	Type	Atoms
3	A	601	EDO	O1-C1-C2-O2
3	A	607	EDO	O1-C1-C2-O2

There are no ring outliers.

2 monomers are involved in 6 short contacts:

Mol	Chain	Res	Type	Clashes	Symm-Clashes
3	A	601	EDO	5	0
3	A	610	EDO	1	0

CONFIDENTIAL VALIDATION REPORT

5.7 Other polymers (i)

There are no such residues in this entry.

5.8 Polymer linkage issues (i)

There are no chain breaks in this entry.

CONFIDENTIAL VALIDATION REPORT

6 Fit of model and data (i)

6.1 Protein, DNA and RNA chains (i)

In the following table, the column labelled '#RSRZ> 2' contains the number (and percentage) of RSRZ outliers, followed by percent RSRZ outliers for the chain as percentile scores relative to all X-ray entries and entries of similar resolution. The OWAB column contains the minimum, median, 95th percentile and maximum values of the occupancy-weighted average B-factor per residue. The column labelled 'Q< 0.9' lists the number of (and percentage) of residues with an average occupancy less than 0.9.

Mol	Chain	Analysed	<RSRZ>	#RSRZ>2			OWAB(Å^2)	Q<0.9
1	A	537/540 (99%)	-0.08	16 (2%)	50	55	7, 15, 29, 46	0

All (16) RSRZ outliers are listed below:

Mol	Chain	Res	Type	RSRZ
1	A	531	LEU	10.3
1	A	-1	ALA	6.8
1	A	150	LYS	3.9
1	A	458	ALA	2.6
1	A	61	ASP	2.6
1	A	123	ARG	2.5
1	A	432	ASP	2.4
1	A	0	GLU	2.3
1	A	305	GLU	2.3
1	A	228	ARG	2.2
1	A	532	GLU	2.2
1	A	533	HIS	2.2
1	A	149	VAL	2.2
1	A	113	GLU	2.1
1	A	153	SER	2.1
1	A	310	GLU	2.1

6.2 Non-standard residues in protein, DNA, RNA chains (i)

There are no non-standard protein/DNA/RNA residues in this entry.

6.3 Carbohydrates (i)

In the following table, the Atoms column lists the number of modelled atoms in the group and the number defined in the chemical component dictionary. The B-factors column lists the minimum,

CONFIDENTIAL VALIDATION REPORT

median, 95th percentile and maximum values of B factors of atoms in the group. The column labelled ‘Q< 0.9’ lists the number of atoms with occupancy less than 0.9.

Mol	Type	Chain	Res	Atoms	RSCC	RSR	B-factors(Å^2)	Q<0.9
2	NAG	E	1	15/15	0.98	0.06	6,10,13,15	0
2	NAG	E	2	14/15	0.98	0.07	6,8,9,9	0

6.4 Ligands (i)

In the following table, the Atoms column lists the number of modelled atoms in the group and the number defined in the chemical component dictionary. The B-factors column lists the minimum, median, 95th percentile and maximum values of B factors of atoms in the group. The column labelled ‘Q< 0.9’ lists the number of atoms with occupancy less than 0.9.

Mol	Type	Chain	Res	Atoms	RSCC	RSR	B-factors(Å^2)	Q<0.9
3	EDO	A	610	4/4	0.78	0.12	25,28,32,35	0
3	EDO	A	605	4/4	0.86	0.18	23,29,30,34	0
3	EDO	A	612	4/4	0.87	0.18	24,27,29,29	0
3	EDO	A	611	4/4	0.88	0.09	27,34,34,34	0
3	EDO	A	609	4/4	0.90	0.12	28,32,33,36	0
3	EDO	A	608	4/4	0.90	0.15	25,30,31,33	0
3	EDO	A	603	4/4	0.91	0.08	20,21,22,25	0
3	EDO	A	607	4/4	0.92	0.07	14,20,20,32	0
3	EDO	A	602	4/4	0.92	0.09	15,17,19,22	0
3	EDO	A	601	4/4	0.93	0.14	17,20,23,23	0
3	EDO	A	606	4/4	0.93	0.13	19,20,24,25	0
3	EDO	A	604	4/4	0.94	0.11	24,27,29,32	0
3	EDO	A	613	4/4	0.94	0.17	26,26,29,30	0
4	MG	A	616	1/1	0.95	0.08	22,22,22,22	0
6	CL	A	620	1/1	0.97	0.06	26,26,26,26	0
4	MG	A	617	1/1	0.98	0.10	24,24,24,24	0
4	MG	A	615	1/1	0.99	0.06	14,14,14,14	0
5	CA	A	618	1/1	0.99	0.04	13,13,13,13	0
5	CA	A	619	1/1	0.99	0.03	10,10,10,10	0
4	MG	A	614	1/1	0.99	0.05	13,13,13,13	0
6	CL	A	621	1/1	1.00	0.03	11,11,11,11	0

6.5 Other polymers (i)

There are no such residues in this entry.

CONFIDENTIAL VALIDATION REPORT

Full wwPDB X-ray Structure Validation Report ⓘ

Feb 27, 2020 – 04:22 PM JST

PDB ID	:	6LZU
Title	:	F411A mutant of chitin-specific solute binding protein from Vibrio harveyi co-crystalized with chitotetraose.
Deposited on	:	2020-02-19
Resolution	:	1.90 Å(reported)

This is a Full wwPDB X-ray Structure Validation Report.

This report is produced by the wwPDB biocuration pipeline after annotation of the structure.

We welcome your comments at *validation@mail.wwpdb.org*
A user guide is available at
https://www.wwpdb.org/validation/2017/XrayValidationReportHelp
with specific help available everywhere you see the ⓘ symbol.

The following versions of software and data (see references ⓘ) were used in the production of this report:

MolProbity	:	4.02b-467
Mogul	:	1.8.0 (224370), CSD as540be (2019)
Xtriage (Phenix)	:	1.13
EDS	:	2.9
buster-report	:	1.1.7 (2018)
Percentile statistics	:	20171227.v01 (using entries in the PDB archive December 27th 2017)
Refmac	:	5.8.0158
CCP4	:	7.0.044 (Gargrove)
Ideal geometry (proteins)	:	Engh & Huber (2001)
Ideal geometry (DNA, RNA)	:	Parkinson et al. (1996)
Validation Pipeline (wwPDB-VP)	:	2.9

CONFIDENTIAL VALIDATION REPORT

1 Overall quality at a glance (i)

The following experimental techniques were used to determine the structure:
X-RAY DIFFRACTION

The reported resolution of this entry is 1.90 Å.

Percentile scores (ranging between 0-100) for global validation metrics of the entry are shown in the following graphic. The table shows the number of entries on which the scores are based.

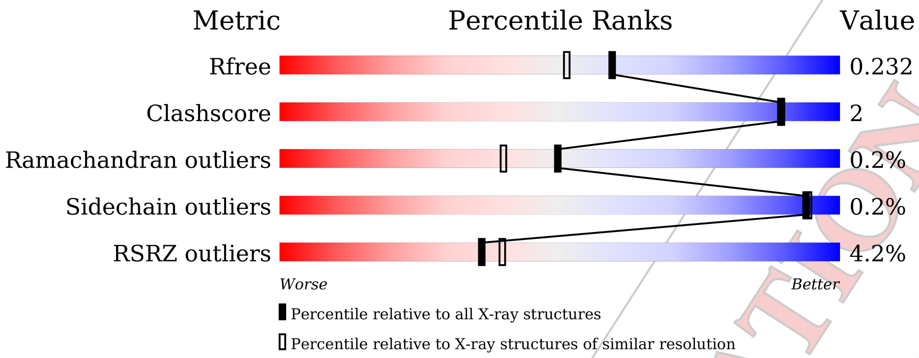

Metric	Whole archive (#Entries)	Similar resolution (#Entries, resolution range(Å))
R_{free}	111664	5502 (1.90-1.90)
Clashscore	122126	6115 (1.90-1.90)
Ramachandran outliers	120053	6048 (1.90-1.90)
Sidechain outliers	120020	6048 (1.90-1.90)
RSRZ outliers	108989	5379 (1.90-1.90)

The table below summarises the geometric issues observed across the polymeric chains and their fit to the electron density. The red, orange, yellow and green segments on the lower bar indicate the fraction of residues that contain outliers for >=3, 2, 1 and 0 types of geometric quality criteria respectively. A grey segment represents the fraction of residues that are not modelled. The numeric value for each fraction is indicated below the corresponding segment, with a dot representing fractions <=5% The upper red bar (where present) indicates the fraction of residues that have poor fit to the electron density. The numeric value is given above the bar.

Mol	Chain	Length	Quality of chain
1	A	538	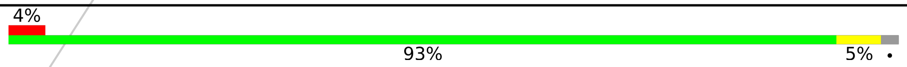

CONFIDENTIAL VALIDATION REPORT

2 Entry composition (i)

There are 7 unique types of molecules in this entry. The entry contains 4459 atoms, of which 0 are hydrogens and 0 are deuteriums.

In the tables below, the ZeroOcc column contains the number of atoms modelled with zero occupancy, the AltConf column contains the number of residues with at least one atom in alternate conformation and the Trace column contains the number of residues modelled with at most 2 atoms.

- Molecule 1 is a protein called Peptide ABC transporter, periplasmic peptide-binding protein.

Mol	Chain	Residues	Atoms					ZeroOcc	AltConf	Trace
			Total	C	N	O	S			
1	A	527	4158	2660	691	796	11	0	1	0

There are 7 discrepancies between the modelled and reference sequences:

Chain	Residue	Modelled	Actual	Comment	Reference
A	411	ALA	PHE	engineered mutation	UNP D0XC84
A	531	HIS	-	expression tag	UNP D0XC84
A	532	HIS	-	expression tag	UNP D0XC84
A	533	HIS	-	expression tag	UNP D0XC84
A	534	HIS	-	expression tag	UNP D0XC84
A	535	HIS	-	expression tag	UNP D0XC84
A	536	HIS	-	expression tag	UNP D0XC84

- Molecule 2 is DI(N-ACETYL-D-GLUCOSAMINE) (three-letter code: CBS) (formula: $C_{16}H_{28}N_2O_{11}$) (labeled as "Ligand of Interest" by author).

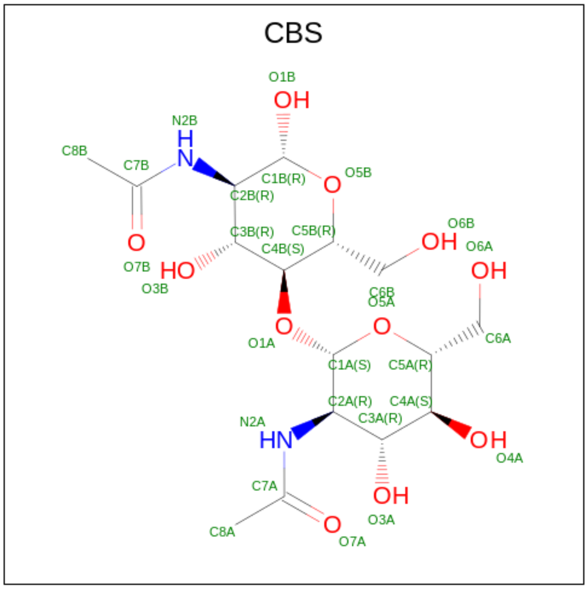

Mol	Chain	Residues	Atoms				ZeroOcc	AltConf
			Total	C	N	O		
2	A	1	29	16	2	11	0	0

- Molecule 3 is 1,2-ETHANEDIOL (three-letter code: EDO) (formula: $C_2H_6O_2$).

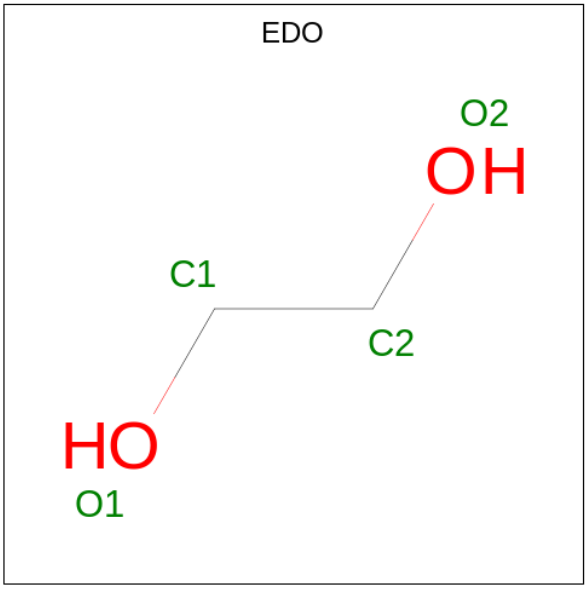

Mol	Chain	Residues	Atoms			ZeroOcc	AltConf
			Total	C	O		
3	A	1	4	2	2	0	0
3	A	1	4	2	2	0	0
3	A	1	4	2	2	0	0

- Molecule 4 is MAGNESIUM ION (three-letter code: MG) (formula: Mg).

Mol	Chain	Residues	Atoms		ZeroOcc	AltConf
			Total	Mg		
4	A	1	1	1	0	0

- Molecule 5 is CALCIUM ION (three-letter code: CA) (formula: Ca).

Mol	Chain	Residues	Atoms		ZeroOcc	AltConf
			Total	Ca		
5	A	2	2	2	0	0

- Molecule 6 is CHLORIDE ION (three-letter code: CL) (formula: Cl).

CONFIDENTIAL VALIDATION REPORT

Mol	Chain	Residues	Atoms		ZeroOcc	AltConf
			Total	Cl		
6	A	1	1	1	0	0

- Molecule 7 is water.

Mol	Chain	Residues	Atoms		ZeroOcc	AltConf
			Total	O		
7	A	256	256	256	0	0

CONFIDENTIAL VALIDATION REPORT

3 Residue-property plots (i)

These plots are drawn for all protein, RNA and DNA chains in the entry. The first graphic for a chain summarises the proportions of the various outlier classes displayed in the second graphic. The second graphic shows the sequence view annotated by issues in geometry and electron density. Residues are color-coded according to the number of geometric quality criteria for which they contain at least one outlier: green = 0, yellow = 1, orange = 2 and red = 3 or more. A red dot above a residue indicates a poor fit to the electron density (RSRZ > 2). Stretches of 2 or more consecutive residues without any outlier are shown as a green connector. Residues present in the sample, but not in the model, are shown in grey.

- Molecule 1: Peptide ABC transporter, periplasmic peptide-binding protein

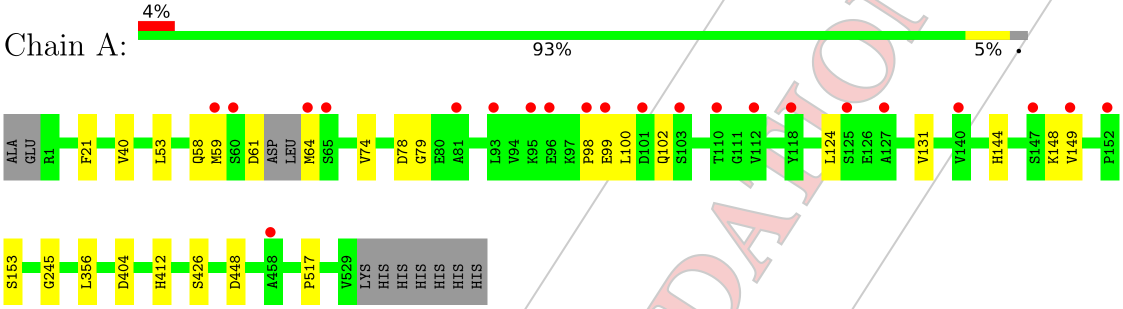

CONFIDENTIAL VALIDATION REPORT

4 Data and refinement statistics (i)

Property	Value	Source
Space group	P 1 21 1	Depositor
Cell constants<br>a, b, c, α, β, γ	62.32Å 57.23Å 82.02Å<br>90.00° 100.49° 90.00°	Depositor
Resolution (Å)	24.15 – 1.90<br>24.15 – 1.90	Depositor<br>EDS
% Data completeness<br>(in resolution range)	90.8 (24.15-1.90)<br>90.7 (24.15-1.90)	Depositor<br>EDS
R_{merge}	0.10	Depositor
R_{sym}	0.10	Depositor
$< I/\sigma(I) >$ [1]	2.80 (at 1.89Å)	Xtriage
Refinement program	PHENIX 1.9_1692	Depositor
R, R_{free}	0.186 , 0.234<br>0.188 , 0.232	Depositor<br>DCC
R_{free} test set	2071 reflections (5.05%)	wwPDB-VP
Wilson B-factor (Å^2)	20.3	Xtriage
Anisotropy	0.149	Xtriage
Bulk solvent k_{sol}(e/Å^3), B_{sol}(Å^2)	0.38 , 61.6	EDS
L-test for twinning[2]	$< \|L\| > = 0.44$, $< L^2 > = 0.27$	Xtriage
Estimated twinning fraction	No twinning to report.	Xtriage
F_o,F_c correlation	0.94	EDS
Total number of atoms	4459	wwPDB-VP
Average B, all atoms (Å^2)	23.0	wwPDB-VP

Xtriage's analysis on translational NCS is as follows: *The largest off-origin peak in the Patterson function is 4.99% of the height of the origin peak. No significant pseudotranslation is detected.*

[1] Intensities estimated from amplitudes.

[2] Theoretical values of $< |L| >$, $< L^2 >$ for acentric reflections are 0.5, 0.333 respectively for untwinned datasets, and 0.375, 0.2 for perfectly twinned datasets.

CONFIDENTIAL VALIDATION REPORT

5 Model quality (i)

5.1 Standard geometry (i)

Bond lengths and bond angles in the following residue types are not validated in this section: CBS, MG, EDO, CA, CL

The Z score for a bond length (or angle) is the number of standard deviations the observed value is removed from the expected value. A bond length (or angle) with $|Z| > 5$ is considered an outlier worth inspection. RMSZ is the root-mean-square of all Z scores of the bond lengths (or angles).

Mol	Chain	Bond lengths		Bond angles	
		RMSZ	$\#|Z| > 5$	RMSZ	$\#|Z| > 5$
1	A	0.40	0/4278	0.51	0/5842

There are no bond length outliers.

There are no bond angle outliers.

There are no chirality outliers.

There are no planarity outliers.

5.2 Too-close contacts (i)

In the following table, the Non-H and H(model) columns list the number of non-hydrogen atoms and hydrogen atoms in the chain respectively. The H(added) column lists the number of hydrogen atoms added and optimized by MolProbity. The Clashes column lists the number of clashes within the asymmetric unit, whereas Symm-Clashes lists symmetry related clashes.

Mol	Chain	Non-H	H(model)	H(added)	Clashes	Symm-Clashes
1	A	4158	0	3879	16	0
2	A	29	0	28	0	0
3	A	12	0	18	0	0
4	A	1	0	0	0	0
5	A	2	0	0	0	0
6	A	1	0	0	0	0
7	A	256	0	0	1	0
All	All	4459	0	3925	16	0

The all-atom clashscore is defined as the number of clashes found per 1000 atoms (including hydrogen atoms). The all-atom clashscore for this structure is 2.

All (16) close contacts within the same asymmetric unit are listed below, sorted by their clash magnitude.

CONFIDENTIAL VALIDATION REPORT

Atom-1	Atom-2	Interatomic distance (Å)	Clash overlap (Å)
1:A:78:ASP:OD2	1:A:144:HIS:NE2	2.21	0.72
1:A:124:LEU:HD21	1:A:131:VAL:HG21	1.76	0.67
1:A:21:PHE:O	1:A:102:GLN:NE2	2.28	0.65
1:A:426:SER:OG	1:A:448:ASP:OD2	2.13	0.65
1:A:40:VAL:HG13	1:A:517:PRO:HB2	1.86	0.58
1:A:61:ASP:HA	1:A:64:MET:N	2.20	0.56
1:A:99:GLU:OE2	1:A:153:SER:OG	2.21	0.54
1:A:58:GLN:NE2	1:A:59[B]:MET:O	2.37	0.52
1:A:78:ASP:OD1	1:A:79:GLY:N	2.44	0.51
1:A:58:GLN:NE2	1:A:59[A]:MET:O	2.45	0.46
1:A:21:PHE:HB3	1:A:100:LEU:HB3	1.99	0.44
1:A:102:GLN:N	7:A:708:HOH:O	2.40	0.44
1:A:53:LEU:HD22	1:A:74:VAL:HG11	2.01	0.42
1:A:356:LEU:O	1:A:404:ASP:HB2	2.19	0.41
1:A:148:LYS:HE2	1:A:148:LYS:HB2	1.89	0.41
1:A:245:GLY:HA3	1:A:412:HIS:HB2	2.02	0.41

There are no symmetry-related clashes.

5.3 Torsion angles (i)

5.3.1 Protein backbone (i)

In the following table, the Percentiles column shows the percent Ramachandran outliers of the chain as a percentile score with respect to all X-ray entries followed by that with respect to entries of similar resolution.

The Analysed column shows the number of residues for which the backbone conformation was analysed, and the total number of residues.

Mol	Chain	Analysed	Favoured	Allowed	Outliers	Percentiles
1	A	524/538 (97%)	504 (96%)	19 (4%)	1 (0%)	49 40

All (1) Ramachandran outliers are listed below:

Mol	Chain	Res	Type
1	A	98	PRO

CONFIDENTIAL VALIDATION REPORT

5.3.2 Protein sidechains (i)

In the following table, the Percentiles column shows the percent sidechain outliers of the chain as a percentile score with respect to all X-ray entries followed by that with respect to entries of similar resolution.

The Analysed column shows the number of residues for which the sidechain conformation was analysed, and the total number of residues.

Mol	Chain	Analysed	Rotameric	Outliers	Percentiles	
1	A	436/461 (95%)	435 (100%)	1 (0%)	94	94

All (1) residues with a non-rotameric sidechain are listed below:

Mol	Chain	Res	Type
1	A	149	VAL

Some sidechains can be flipped to improve hydrogen bonding and reduce clashes. There are no such sidechains identified.

5.3.3 RNA (i)

There are no RNA molecules in this entry.

5.4 Non-standard residues in protein, DNA, RNA chains (i)

There are no non-standard protein/DNA/RNA residues in this entry.

5.5 Carbohydrates (i)

There are no carbohydrates in this entry.

5.6 Ligand geometry (i)

Of 8 ligands modelled in this entry, 4 are monoatomic - leaving 4 for Mogul analysis.

In the following table, the Counts columns list the number of bonds (or angles) for which Mogul statistics could be retrieved, the number of bonds (or angles) that are observed in the model and the number of bonds (or angles) that are defined in the Chemical Component Dictionary. The Link column lists molecule types, if any, to which the group is linked. The Z score for a bond length (or angle) is the number of standard deviations the observed value is removed from the expected value. A bond length (or angle) with $|Z| > 2$ is considered an outlier worth inspection. RMSZ is the root-mean-square of all Z scores of the bond lengths (or angles).

CONFIDENTIAL VALIDATION REPORT

Mol	Type	Chain	Res	Link	Bond lengths			Bond angles		
					Counts	RMSZ	#|Z| > 2	Counts	RMSZ	#|Z| > 2
3	EDO	A	604	-	3,3,3	0.50	0	2,2,2	0.32	0
3	EDO	A	603	-	3,3,3	0.46	0	2,2,2	0.39	0
2	CBS	A	601	-	30,30,30	0.47	0	43,43,43	0.82	1 (2%)
3	EDO	A	602	-	3,3,3	0.50	0	2,2,2	0.27	0

In the following table, the Chirals column lists the number of chiral outliers, the number of chiral centers analysed, the number of these observed in the model and the number defined in the Chemical Component Dictionary. Similar counts are reported in the Torsion and Rings columns. '-' means no outliers of that kind were identified.

Mol	Type	Chain	Res	Link	Chirals	Torsions	Rings
3	EDO	A	604	-	-	1/1/1/1	-
3	EDO	A	603	-	-	0/1/1/1	-
2	CBS	A	601	-	-	0/16/56/56	0/2/2/2
3	EDO	A	602	-	-	0/1/1/1	-

There are no bond length outliers.

All (1) bond angle outliers are listed below:

Mol	Chain	Res	Type	Atoms	Z	Observed(^{o})	Ideal(^{o})
2	A	601	CBS	C4A-C3A-C2A	2.34	113.82	110.35

There are no chirality outliers.

All (1) torsion outliers are listed below:

Mol	Chain	Res	Type	Atoms
3	A	604	EDO	O1-C1-C2-O2

There are no ring outliers.

No monomer is involved in short contacts.

The following is a two-dimensional graphical depiction of Mogul quality analysis of bond lengths, bond angles, torsion angles, and ring geometry for all instances of the Ligand of Interest. In addition, ligands with molecular weight > 250 and outliers as shown on the validation Tables will also be included. For torsion angles, if less then 5% of the Mogul distribution of torsion angles is within 10 degrees of the torsion angle in question, then that torsion angle is considered an outlier. Any bond that is central to one or more torsion angles identified as an outlier by Mogul will be highlighted in the graph. For rings, the root-mean-square deviation (RMSD) between the ring in question and similar rings identified by Mogul is calculated over all ring torsion angles. If the average RMSD is greater than 60 degrees and the minimal RMSD between the ring in question and any Mogul-identified rings is also greater than 60 degrees, then that ring is considered an outlier.

CONFIDENTIAL VALIDATION REPORT

The outliers are highlighted in purple. The color gray indicates Mogul did not find sufficient equivalents in the CSD to analyse the geometry.

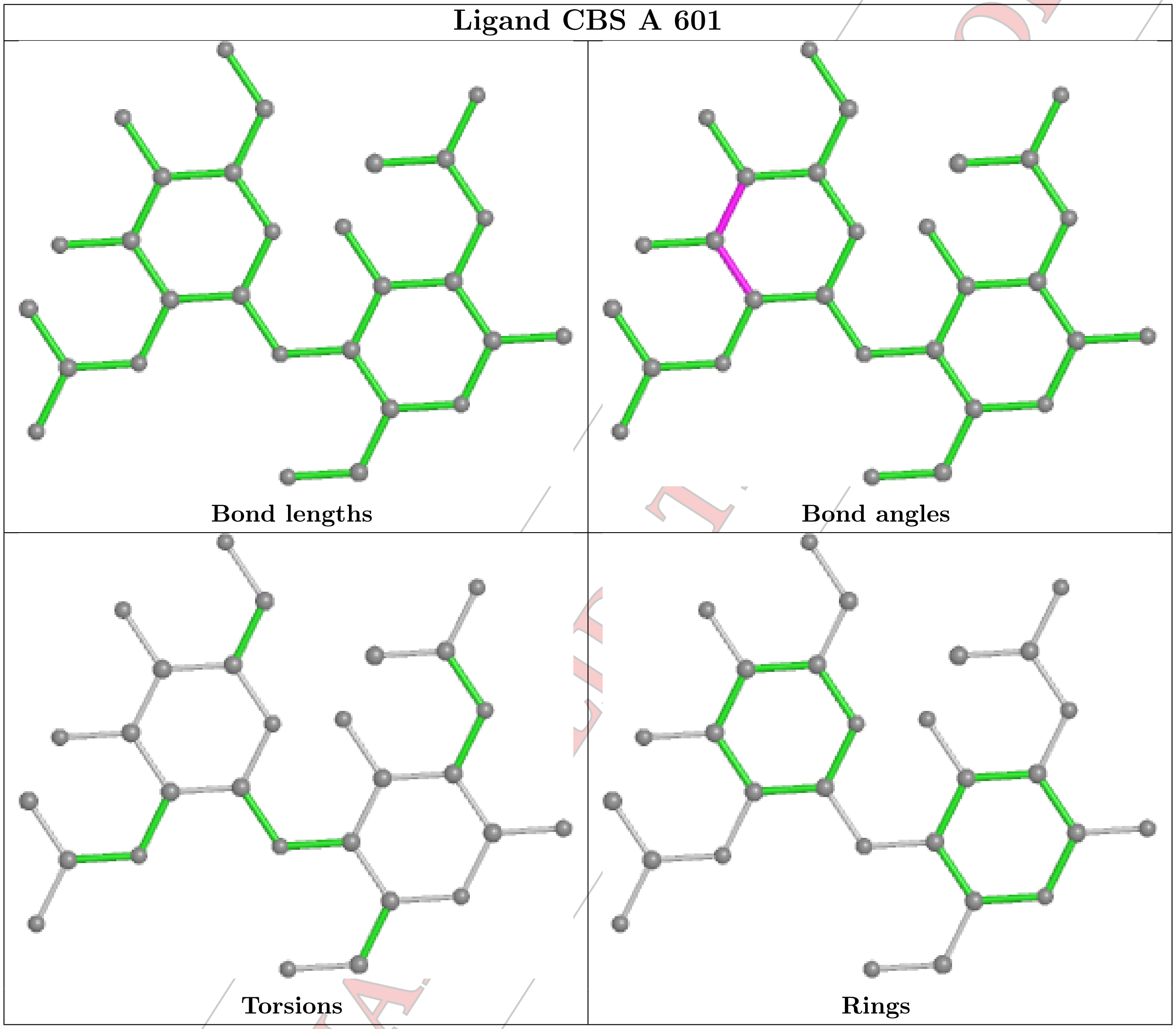

5.7 Other polymers (i)

There are no such residues in this entry.

5.8 Polymer linkage issues (i)

There are no chain breaks in this entry.

CONFIDENTIAL REPORT

6 Fit of model and data

6.1 Protein, DNA and RNA chains

In the following table, the column labelled '#RSRZ> 2' contains the number (and percentage) of RSRZ outliers, followed by percent RSRZ outliers for the chain as percentile scores relative to all X-ray entries and entries of similar resolution. The OWAB column contains the minimum, median, 95th percentile and maximum values of the occupancy-weighted average B-factor per residue. The column labelled 'Q< 0.9' lists the number of (and percentage) of residues with an average occupancy less than 0.9.

Mol	Chain	Analysed	<RSRZ>	#RSRZ>2			OWAB(Å^2)	Q<0.9
1	A	527/538 (97%)	0.03	22 (4%)	36	40	10, 20, 49, 66	0

All (22) RSRZ outliers are listed below:

Mol	Chain	Res	Type	RSRZ
1	A	149	VAL	3.8
1	A	93	LEU	3.5
1	A	147	SER	3.4
1	A	458	ALA	3.1
1	A	96	GLU	3.1
1	A	99	GLU	3.0
1	A	98	PRO	2.8
1	A	127	ALA	2.8
1	A	81	ALA	2.7
1	A	101	ASP	2.7
1	A	152	PRO	2.6
1	A	118	TYR	2.6
1	A	60	SER	2.6
1	A	112	VAL	2.6
1	A	103	SER	2.5
1	A	110	THR	2.4
1	A	59[A]	MET	2.4
1	A	125	SER	2.2
1	A	65	SER	2.1
1	A	64	MET	2.1
1	A	95	LYS	2.0
1	A	140	VAL	2.0

6.2 Non-standard residues in protein, DNA, RNA chains

There are no non-standard protein/DNA/RNA residues in this entry.

CONFIDENTIAL VALIDATION REPORT

6.3 Carbohydrates (i)

There are no carbohydrates in this entry.

6.4 Ligands (i)

In the following table, the Atoms column lists the number of modelled atoms in the group and the number defined in the chemical component dictionary. The B-factors column lists the minimum, median, 95th percentile and maximum values of B factors of atoms in the group. The column labelled 'Q< 0.9' lists the number of atoms with occupancy less than 0.9.

Mol	Type	Chain	Res	Atoms	RSCC	RSR	B-factors(Å^2)	Q<0.9
3	EDO	A	604	4/4	0.84	0.27	30,35,38,40	0
3	EDO	A	603	4/4	0.92	0.24	27,33,33,39	0
6	CL	A	608	1/1	0.93	0.09	44,44,44,44	0
2	CBS	A	601	29/29	0.95	0.09	9,11,14,21	0
3	EDO	A	602	4/4	0.95	0.08	23,29,31,33	0
4	MG	A	605	1/1	0.98	0.08	24,24,24,24	0
5	CA	A	606	1/1	0.99	0.04	16,16,16,16	0
5	CA	A	607	1/1	0.99	0.03	26,26,26,26	0

The following is a graphical depiction of the model fit to experimental electron density of all instances of the Ligand of Interest. In addition, ligands with molecular weight > 250 and outliers as shown on the geometry validation Tables will also be included. Each fit is shown from different orientation to approximate a three-dimensional view.

CONFIDENTIAL VALIDATION REPORT

Electron density around CBS A 601:

2mF$_o$-DF$_c$ (at 0.7 rmsd) in gray
mF$_o$-DF$_c$ (at 3 rmsd) in purple (negative)
and green (positive)

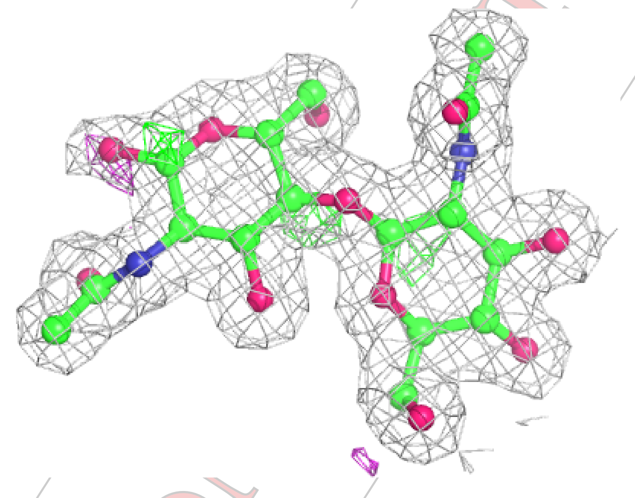

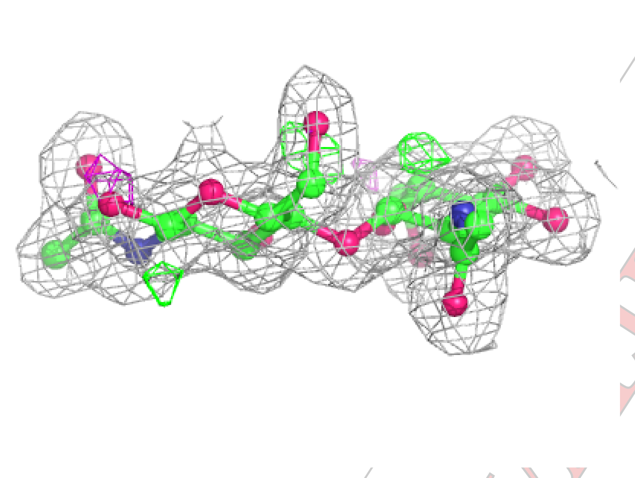

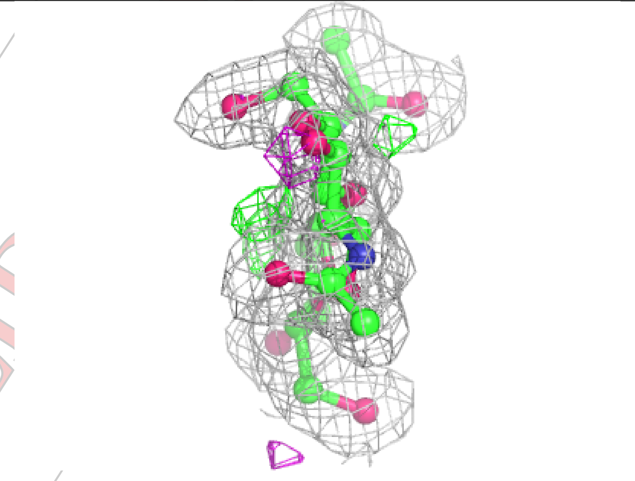

6.5 Other polymers (i)

There are no such residues in this entry.

CONFIDENTIAL

Full wwPDB X-ray Structure Validation Report (i)

Feb 27, 2020 – 04:27 PM JST

PDB ID	:	6LZV
Title	:	F437A mutant of chitin-specific solute binding protein from Vibrio harveyi co-crystalized with chitotetraose.
Deposited on	:	2020-02-19
Resolution	:	2.20 Å(reported)

This is a Full wwPDB X-ray Structure Validation Report.

This report is produced by the wwPDB biocuration pipeline after annotation of the structure.

We welcome your comments at *validation@mail.wwpdb.org*
A user guide is available at
https://www.wwpdb.org/validation/2017/XrayValidationReportHelp
with specific help available everywhere you see the (i) symbol.

CONFIDENTIAL VALIDATION REPORT

The following versions of software and data (see references (i)) were used in the production of this report:

MolProbity	:	4.02b-467
Mogul	:	1.8.0 (224370), CSD as540be (2019)
Xtriage (Phenix)	:	1.13
EDS	:	2.9
buster-report	:	1.1.7 (2018)
Percentile statistics	:	20171227.v01 (using entries in the PDB archive December 27th 2017)
Refmac	:	5.8.0158
CCP4	:	7.0.044 (Gargrove)
Ideal geometry (proteins)	:	Engh & Huber (2001)
Ideal geometry (DNA, RNA)	:	Parkinson et al. (1996)
Validation Pipeline (wwPDB-VP)	:	2.9

1 Overall quality at a glance (i)

The following experimental techniques were used to determine the structure:
X-RAY DIFFRACTION

The reported resolution of this entry is 2.20 Å.

Percentile scores (ranging between 0-100) for global validation metrics of the entry are shown in the following graphic. The table shows the number of entries on which the scores are based.

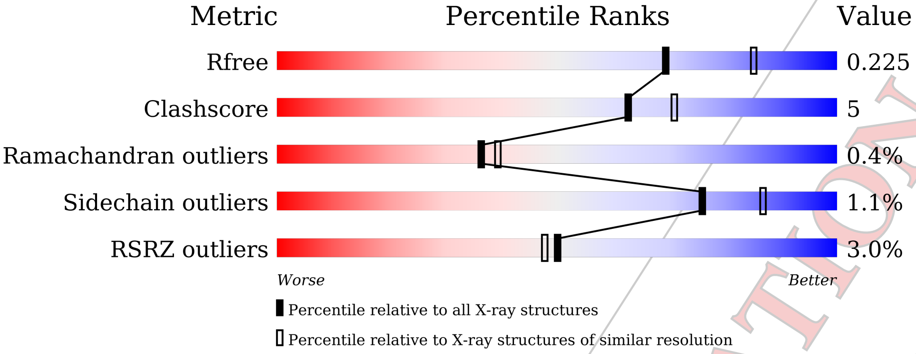

Metric	Whole archive (#Entries)	Similar resolution (#Entries, resolution range(Å))
R_{free}	111664	4343 (2.20-2.20)
Clashscore	122126	5027 (2.20-2.20)
Ramachandran outliers	120053	4952 (2.20-2.20)
Sidechain outliers	120020	4953 (2.20-2.20)
RSRZ outliers	108989	4245 (2.20-2.20)

The table below summarises the geometric issues observed across the polymeric chains and their fit to the electron density. The red, orange, yellow and green segments on the lower bar indicate the fraction of residues that contain outliers for >=3, 2, 1 and 0 types of geometric quality criteria respectively. A grey segment represents the fraction of residues that are not modelled. The numeric value for each fraction is indicated below the corresponding segment, with a dot representing fractions <=5% The upper red bar (where present) indicates the fraction of residues that have poor fit to the electron density. The numeric value is given above the bar.

Mol	Chain	Length	Quality of chain
1	A	540	3% 88% 10% •

2 Entry composition (i)

There are 6 unique types of molecules in this entry. The entry contains 4473 atoms, of which 0 are hydrogens and 0 are deuteriums.

In the tables below, the ZeroOcc column contains the number of atoms modelled with zero occupancy, the AltConf column contains the number of residues with at least one atom in alternate conformation and the Trace column contains the number of residues modelled with at most 2 atoms.

- Molecule 1 is a protein called Peptide ABC transporter, periplasmic peptide-binding protein.

Mol	Chain	Residues	Atoms					ZeroOcc	AltConf	Trace
			Total	C	N	O	S			
1	A	535	4301	2751	718	820	12	0	4	0

There are 9 discrepancies between the modelled and reference sequences:

Chain	Residue	Modelled	Actual	Comment	Reference
A	437	ALA	PHE	engineered mutation	UNP D0XC84
A	531	LEU	-	expression tag	UNP D0XC84
A	532	GLU	-	expression tag	UNP D0XC84
A	533	HIS	-	expression tag	UNP D0XC84
A	534	HIS	-	expression tag	UNP D0XC84
A	535	HIS	-	expression tag	UNP D0XC84
A	536	HIS	-	expression tag	UNP D0XC84
A	537	HIS	-	expression tag	UNP D0XC84
A	538	HIS	-	expression tag	UNP D0XC84

- Molecule 2 is DI(N-ACETYL-D-GLUCOSAMINE) (three-letter code: CBS) (formula: $C_{16}H_{28}N_2O_{11}$) (labeled as "Ligand of Interest" by author).

CONFIDENTIAL VALIDATION REPORT

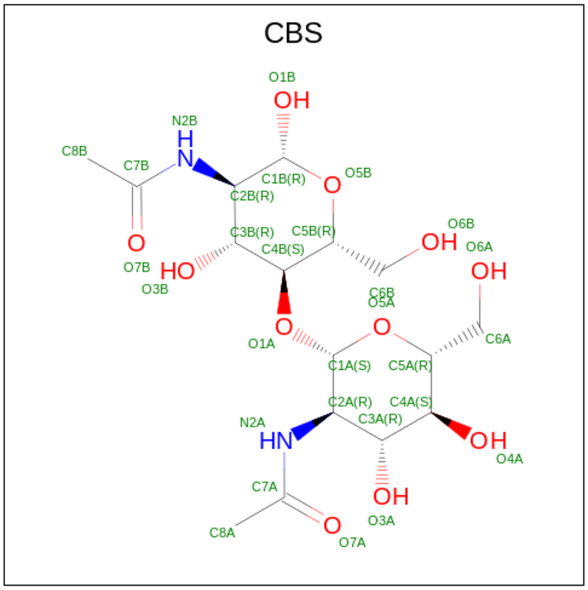

Mol	Chain	Residues	Atoms	ZeroOcc	AltConf
2	A	1	Total C N O<br>29 16 2 11	0	0

- Molecule 3 is 1,2-ETHANEDIOL (three-letter code: EDO) (formula: $C_2H_6O_2$).

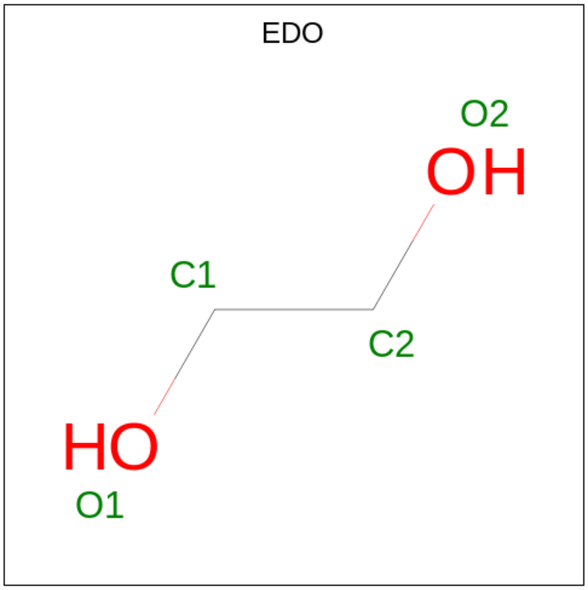

Mol	Chain	Residues	Atoms	ZeroOcc	AltConf
3	A	1	Total C O<br>4 2 2	0	0

- Molecule 4 is CHLORIDE ION (three-letter code: CL) (formula: Cl).

Mol	Chain	Residues	Atoms		ZeroOcc	AltConf
			Total	Cl		
4	A	3	3	3	0	0

- Molecule 5 is CALCIUM ION (three-letter code: CA) (formula: Ca).

Mol	Chain	Residues	Atoms		ZeroOcc	AltConf
			Total	Ca		
5	A	3	3	3	0	0

- Molecule 6 is water.

Mol	Chain	Residues	Atoms		ZeroOcc	AltConf
			Total	O		
6	A	133	133	133	0	0

CONFIDENTIAL VALIDATION REPORT

3 Residue-property plots (i)

These plots are drawn for all protein, RNA and DNA chains in the entry. The first graphic for a chain summarises the proportions of the various outlier classes displayed in the second graphic. The second graphic shows the sequence view annotated by issues in geometry and electron density. Residues are color-coded according to the number of geometric quality criteria for which they contain at least one outlier: green = 0, yellow = 1, orange = 2 and red = 3 or more. A red dot above a residue indicates a poor fit to the electron density (RSRZ > 2). Stretches of 2 or more consecutive residues without any outlier are shown as a green connector. Residues present in the sample, but not in the model, are shown in grey.

- Molecule 1: Peptide ABC transporter, periplasmic peptide-binding protein

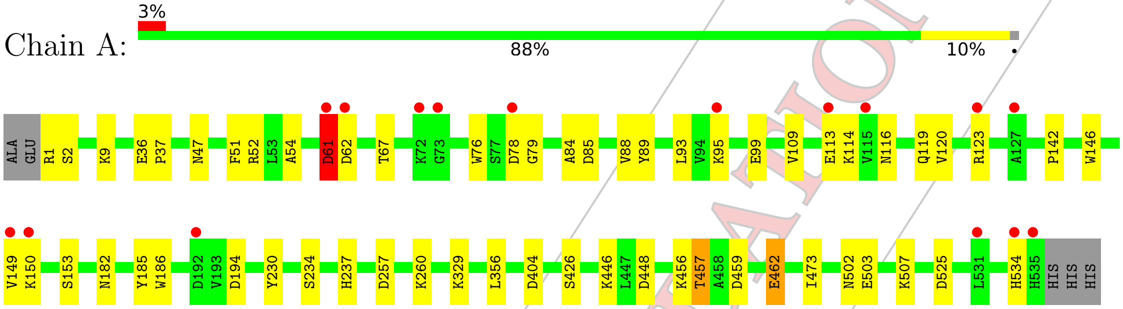

CONFIDENTIAL VALIDATION REPORT

4 Data and refinement statistics (i)

Property	Value	Source
Space group	P 1 21 1	Depositor
Cell constants<br>a, b, c, α, β, γ	59.52Å 56.96Å 85.15Å<br>90.00° 101.36° 90.00°	Depositor
Resolution (Å)	19.90 – 2.20<br>19.90 – 2.20	Depositor<br>EDS
% Data completeness<br>(in resolution range)	94.1 (19.90-2.20)<br>94.1 (19.90-2.20)	Depositor<br>EDS
R_{merge}	0.07	Depositor
R_{sym}	0.04	Depositor
$< I/\sigma(I) >$ [1]	2.16 (at 2.19Å)	Xtriage
Refinement program	PHENIX 1.9_1692	Depositor
R, R_{free}	0.171 , 0.225<br>0.173 , 0.225	Depositor<br>DCC
R_{free} test set	1317 reflections (4.88%)	wwPDB-VP
Wilson B-factor (Å^2)	31.5	Xtriage
Anisotropy	0.112	Xtriage
Bulk solvent k_{sol}(e/Å^3), B_{sol}(Å^2)	0.34 , 49.8	EDS
L-test for twinning[2]	$< \|L\| > = 0.48$, $< L^2 > = 0.31$	Xtriage
Estimated twinning fraction	No twinning to report.	Xtriage
F_o,F_c correlation	0.96	EDS
Total number of atoms	4473	wwPDB-VP
Average B, all atoms (Å^2)	37.0	wwPDB-VP

Xtriage's analysis on translational NCS is as follows: *The largest off-origin peak in the Patterson function is 5.40% of the height of the origin peak. No significant pseudotranslation is detected.*

[1] Intensities estimated from amplitudes.
[2] Theoretical values of $< |L| >$, $< L^2 >$ for acentric reflections are 0.5, 0.333 respectively for untwinned datasets, and 0.375, 0.2 for perfectly twinned datasets.

CONFIDENTIAL VALIDATION REPORT

5 Model quality (i)

5.1 Standard geometry (i)

Bond lengths and bond angles in the following residue types are not validated in this section: CBS, CA, EDO, CL

The Z score for a bond length (or angle) is the number of standard deviations the observed value is removed from the expected value. A bond length (or angle) with $|Z| > 5$ is considered an outlier worth inspection. RMSZ is the root-mean-square of all Z scores of the bond lengths (or angles).

Mol	Chain	Bond lengths		Bond angles	
		RMSZ	#$|Z|$ >5	RMSZ	#$|Z|$ >5
1	A	0.44	0/4427	0.56	0/6030

There are no bond length outliers.

There are no bond angle outliers.

There are no chirality outliers.

There are no planarity outliers.

5.2 Too-close contacts (i)

In the following table, the Non-H and H(model) columns list the number of non-hydrogen atoms and hydrogen atoms in the chain respectively. The H(added) column lists the number of hydrogen atoms added and optimized by MolProbity. The Clashes column lists the number of clashes within the asymmetric unit, whereas Symm-Clashes lists symmetry related clashes.

Mol	Chain	Non-H	H(model)	H(added)	Clashes	Symm-Clashes
1	A	4301	0	4079	40	0
2	A	29	0	28	1	0
3	A	4	0	6	0	0
4	A	3	0	0	0	0
5	A	3	0	0	0	0
6	A	133	0	0	1	0
All	All	4473	0	4113	40	0

The all-atom clashscore is defined as the number of clashes found per 1000 atoms (including hydrogen atoms). The all-atom clashscore for this structure is 5.

All (40) close contacts within the same asymmetric unit are listed below, sorted by their clash magnitude.

CONFIDENTIAL VALIDATION REPORT

Atom-1	Atom-2	Interatomic distance (Å)	Clash overlap (Å)
1:A:150:LYS:HD2	1:A:150:LYS:H	1.58	0.69
1:A:507:LYS:HE2	1:A:525:ASP:OD2	1.93	0.68
1:A:459:ASP:HB3	1:A:462[B]:GLU:HB3	1.76	0.67
1:A:150:LYS:CD	1:A:150:LYS:H	2.09	0.64
1:A:36:GLU:OE2	1:A:52[B]:ARG:NH2	2.30	0.64
1:A:457:THR:HG22	1:A:459:ASP:H	1.65	0.62
1:A:99:GLU:OE2	1:A:153:SER:OG	2.18	0.62
1:A:182:ASN:HB3	1:A:185:TYR:HB2	1.90	0.54
1:A:113:GLU:OE2	1:A:123:ARG:NH1	2.36	0.54
1:A:257:ASP:OD1	1:A:260:LYS:HG3	2.10	0.51
1:A:61:ASP:N	1:A:61:ASP:OD1	2.44	0.51
1:A:459:ASP:HB3	1:A:462[A]:GLU:HB2	1.94	0.50
1:A:78:ASP:OD1	1:A:79:GLY:N	2.45	0.49
1:A:89:TYR:CZ	1:A:93:LEU:HD22	2.46	0.49
1:A:88:VAL:HG21	1:A:114:LYS:HB2	1.94	0.49
1:A:426:SER:OG	1:A:448:ASP:OD2	2.22	0.48
1:A:2:SER:O	1:A:194:ASP:HB2	2.15	0.47
1:A:502:ASN:HB2	1:A:503:GLU:OE2	2.14	0.46
1:A:52[B]:ARG:NH2	1:A:186:TRP:HZ2	2.13	0.46
1:A:116:ASN:OD1	1:A:119:GLN:HB2	2.15	0.46
1:A:457:THR:CG2	1:A:459:ASP:H	2.27	0.46
1:A:36:GLU:HG3	1:A:37:PRO:HD2	1.97	0.46
1:A:146:TRP:O	1:A:149:VAL:HG22	2.16	0.45
1:A:230:TYR:CZ	1:A:237:HIS:HB3	2.52	0.45
1:A:95:LYS:HE3	1:A:109:VAL:O	2.17	0.44
1:A:1:ARG:N	6:A:707:HOH:O	2.49	0.44
1:A:52[B]:ARG:HD2	1:A:52[B]:ARG:HA	1.69	0.44
1:A:62:ASP:N	1:A:62:ASP:OD1	2.48	0.44
1:A:9:LYS:HD2	2:A:601:CBS:H62B	1.99	0.44
1:A:356:LEU:O	1:A:404:ASP:HB2	2.17	0.44
1:A:89:TYR:OH	1:A:93:LEU:HD22	2.17	0.43
1:A:84:ALA:O	1:A:88:VAL:HG23	2.18	0.43
1:A:85:ASP:HA	1:A:88:VAL:HB	2.01	0.43
1:A:61:ASP:HB2	1:A:62:ASP:H	1.73	0.42
1:A:67:THR:HA	1:A:120:VAL:O	2.20	0.41
1:A:446:LYS:HD3	1:A:473:ILE:HD11	2.03	0.40
1:A:234:SER:HB3	1:A:237:HIS:CE1	2.57	0.40
1:A:456:LYS:O	1:A:456:LYS:HG2	2.20	0.40
1:A:51:PHE:HB3	1:A:54:ALA:O	2.21	0.40
1:A:76:TRP:CE3	1:A:142:PRO:HG3	2.56	0.40

There are no symmetry-related clashes.

CONFIDENTIAL VALIDATION REPORT

5.3 Torsion angles (i)

5.3.1 Protein backbone (i)

In the following table, the Percentiles column shows the percent Ramachandran outliers of the chain as a percentile score with respect to all X-ray entries followed by that with respect to entries of similar resolution.

The Analysed column shows the number of residues for which the backbone conformation was analysed, and the total number of residues.

Mol	Chain	Analysed	Favoured	Allowed	Outliers	Percentiles
1	A	537/540 (99%)	505 (94%)	30 (6%)	2 (0%)	36 39

All (2) Ramachandran outliers are listed below:

Mol	Chain	Res	Type
1	A	61	ASP
1	A	534	HIS

5.3.2 Protein sidechains (i)

In the following table, the Percentiles column shows the percent sidechain outliers of the chain as a percentile score with respect to all X-ray entries followed by that with respect to entries of similar resolution.

The Analysed column shows the number of residues for which the sidechain conformation was analysed, and the total number of residues.

Mol	Chain	Analysed	Rotameric	Outliers	Percentiles
1	A	459/463 (99%)	453 (99%)	6 (1%)	71 83

All (6) residues with a non-rotameric sidechain are listed below:

Mol	Chain	Res	Type
1	A	47	ASN
1	A	61	ASP
1	A	329	LYS
1	A	457	THR
1	A	462[A]	GLU
1	A	462[B]	GLU

Some sidechains can be flipped to improve hydrogen bonding and reduce clashes. All (1) such sidechains are listed below:

CONFIDENTIAL VALIDATION REPORT

Mol	Chain	Res	Type
1	A	213	ASN

5.3.3 RNA (i)

There are no RNA molecules in this entry.

5.4 Non-standard residues in protein, DNA, RNA chains (i)

There are no non-standard protein/DNA/RNA residues in this entry.

5.5 Carbohydrates (i)

There are no carbohydrates in this entry.

5.6 Ligand geometry (i)

Of 8 ligands modelled in this entry, 6 are monoatomic - leaving 2 for Mogul analysis.

In the following table, the Counts columns list the number of bonds (or angles) for which Mogul statistics could be retrieved, the number of bonds (or angles) that are observed in the model and the number of bonds (or angles) that are defined in the Chemical Component Dictionary. The Link column lists molecule types, if any, to which the group is linked. The Z score for a bond length (or angle) is the number of standard deviations the observed value is removed from the expected value. A bond length (or angle) with $|Z| > 2$ is considered an outlier worth inspection. RMSZ is the root-mean-square of all Z scores of the bond lengths (or angles).

Mol	Type	Chain	Res	Link	Bond lengths			Bond angles		
					Counts	RMSZ	$\#\|Z\| > 2$	Counts	RMSZ	$\#\|Z\| > 2$
3	EDO	A	602	-	3,3,3	0.56	0	2,2,2	0.32	0
2	CBS	A	601	-	30,30,30	0.47	0	43,43,43	1.21	3 (6%)

In the following table, the Chirals column lists the number of chiral outliers, the number of chiral centers analysed, the number of these observed in the model and the number defined in the Chemical Component Dictionary. Similar counts are reported in the Torsion and Rings columns. '-' means no outliers of that kind were identified.

Mol	Type	Chain	Res	Link	Chirals	Torsions	Rings
3	EDO	A	602	-	-	1/1/1/1	-
2	CBS	A	601	-	-	0/16/56/56	0/2/2/2

There are no bond length outliers.

CONFIDENTIAL VALIDATION REPORT

All (3) bond angle outliers are listed below:

Mol	Chain	Res	Type	Atoms	Z	Observed(°)	Ideal(°)
2	A	601	CBS	C1B-C2B-C3B	-3.93	105.19	110.54
2	A	601	CBS	C1B-O5B-C5B	-2.60	108.71	113.68
2	A	601	CBS	C1A-O1A-C4B	-2.20	112.47	117.97

There are no chirality outliers.

All (1) torsion outliers are listed below:

Mol	Chain	Res	Type	Atoms
3	A	602	EDO	O1-C1-C2-O2

There are no ring outliers.

1 monomer is involved in 1 short contact:

Mol	Chain	Res	Type	Clashes	Symm-Clashes
2	A	601	CBS	1	0

The following is a two-dimensional graphical depiction of Mogul quality analysis of bond lengths, bond angles, torsion angles, and ring geometry for all instances of the Ligand of Interest. In addition, ligands with molecular weight > 250 and outliers as shown on the validation Tables will also be included. For torsion angles, if less then 5% of the Mogul distribution of torsion angles is within 10 degrees of the torsion angle in question, then that torsion angle is considered an outlier. Any bond that is central to one or more torsion angles identified as an outlier by Mogul will be highlighted in the graph. For rings, the root-mean-square deviation (RMSD) between the ring in question and similar rings identified by Mogul is calculated over all ring torsion angles. If the average RMSD is greater than 60 degrees and the minimal RMSD between the ring in question and any Mogul-identified rings is also greater than 60 degrees, then that ring is considered an outlier. The outliers are highlighted in purple. The color gray indicates Mogul did not find sufficient equivalents in the CSD to analyse the geometry.

CONFIDENTIAL VALIDATION REPORT

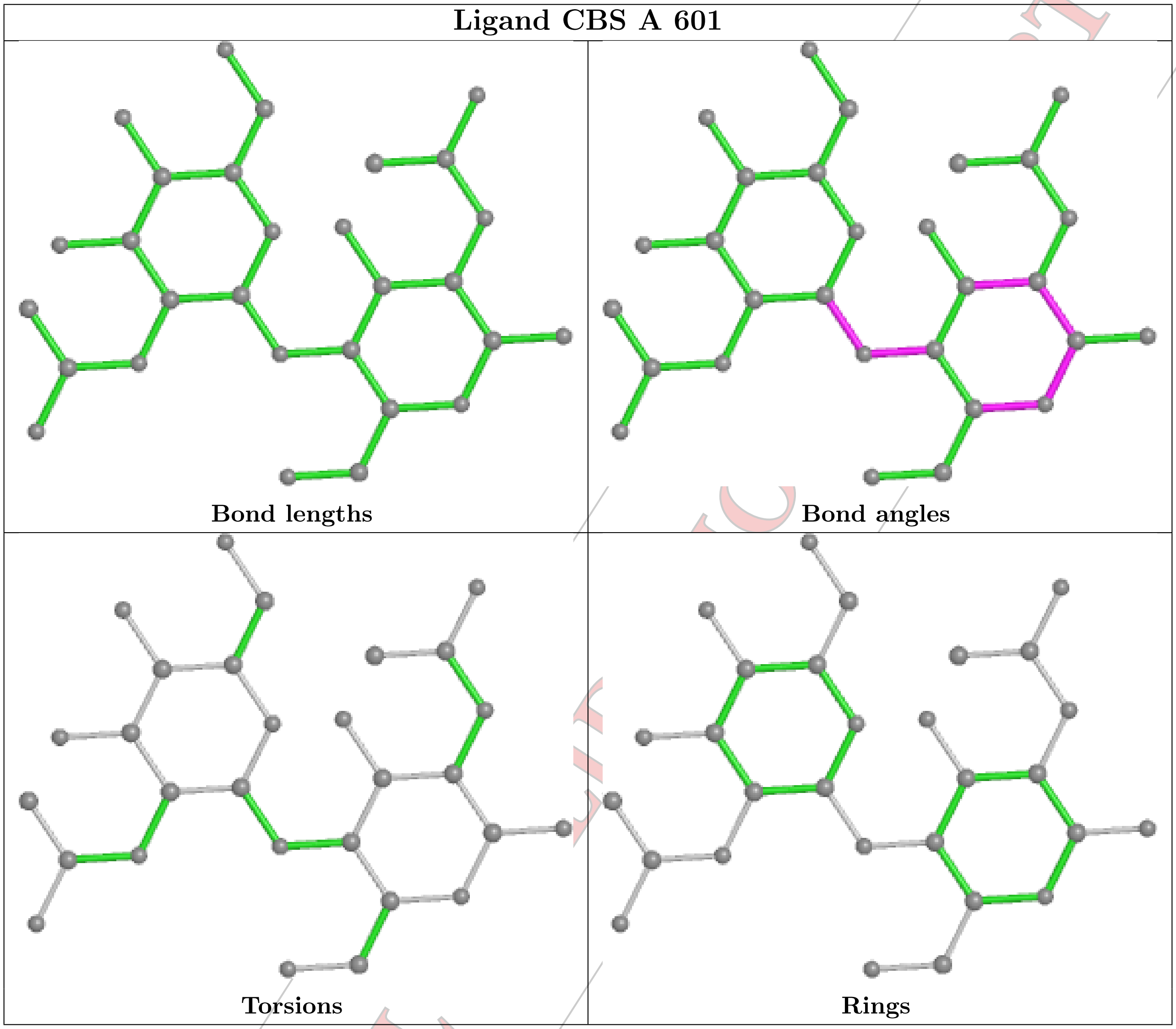

5.7 Other polymers (i)

There are no such residues in this entry.

5.8 Polymer linkage issues (i)

There are no chain breaks in this entry.

CONFIDENTIAL

6 Fit of model and data (i)

6.1 Protein, DNA and RNA chains (i)

In the following table, the column labelled '#RSRZ> 2' contains the number (and percentage) of RSRZ outliers, followed by percent RSRZ outliers for the chain as percentile scores relative to all X-ray entries and entries of similar resolution. The OWAB column contains the minimum, median, 95th percentile and maximum values of the occupancy-weighted average B-factor per residue. The column labelled 'Q< 0.9' lists the number of (and percentage) of residues with an average occupancy less than 0.9.

Mol	Chain	Analysed	<RSRZ>	#RSRZ>2			OWAB(Å^2)	Q<0.9
1	A	535/540 (99%)	-0.10	16 (2%)	50	48	15, 35, 63, 84	0

All (16) RSRZ outliers are listed below:

Mol	Chain	Res	Type	RSRZ
1	A	534	HIS	4.3
1	A	150	LYS	3.9
1	A	61	ASP	3.8
1	A	115	VAL	3.2
1	A	535	HIS	3.0
1	A	73	GLY	2.8
1	A	62	ASP	2.7
1	A	72	LYS	2.5
1	A	113	GLU	2.3
1	A	149	VAL	2.2
1	A	192	ASP	2.1
1	A	531	LEU	2.1
1	A	127	ALA	2.1
1	A	95	LYS	2.1
1	A	78	ASP	2.1
1	A	123	ARG	2.0

6.2 Non-standard residues in protein, DNA, RNA chains (i)

There are no non-standard protein/DNA/RNA residues in this entry.

6.3 Carbohydrates (i)

There are no carbohydrates in this entry.

CONFIDENTIAL VALIDATION REPORT

6.4 Ligands ⓘ

In the following table, the Atoms column lists the number of modelled atoms in the group and the number defined in the chemical component dictionary. The B-factors column lists the minimum, median, 95th percentile and maximum values of B factors of atoms in the group. The column labelled 'Q< 0.9' lists the number of atoms with occupancy less than 0.9.

Mol	Type	Chain	Res	Atoms	RSCC	RSR	B-factors(Å^2)	Q<0.9
5	CA	A	607	1/1	0.65	0.10	70,70,70,70	0
4	CL	A	605	1/1	0.85	0.12	67,67,67,67	0
3	EDO	A	602	4/4	0.87	0.18	33,42,49,50	0
4	CL	A	604	1/1	0.93	0.05	58,58,58,58	0
5	CA	A	608	1/1	0.94	0.04	47,47,47,47	0
2	CBS	A	601	29/29	0.96	0.11	16,21,26,31	0
4	CL	A	603	1/1	0.99	0.09	28,28,28,28	0
5	CA	A	606	1/1	1.00	0.03	22,22,22,22	0

The following is a graphical depiction of the model fit to experimental electron density of all instances of the Ligand of Interest. In addition, ligands with molecular weight > 250 and outliers as shown on the geometry validation Tables will also be included. Each fit is shown from different orientation to approximate a three-dimensional view.

CONFIDENTIAL VALIDATION REPORT

Electron density around CBS A 601:

$2mF_o$-DF_c (at 0.7 rmsd) in gray
mF_o-DF_c (at 3 rmsd) in purple (negative)
and green (positive)

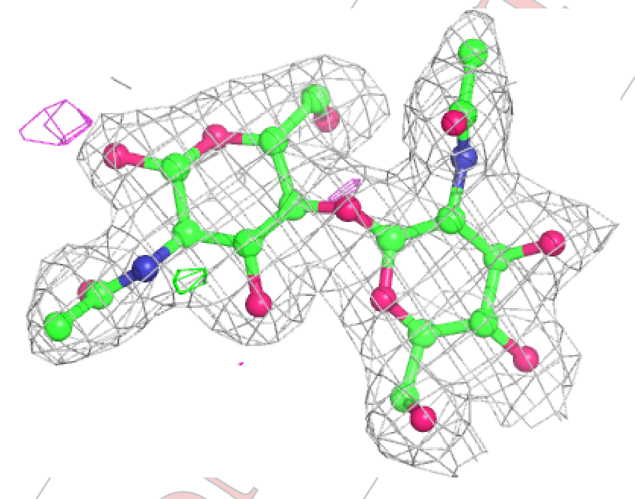

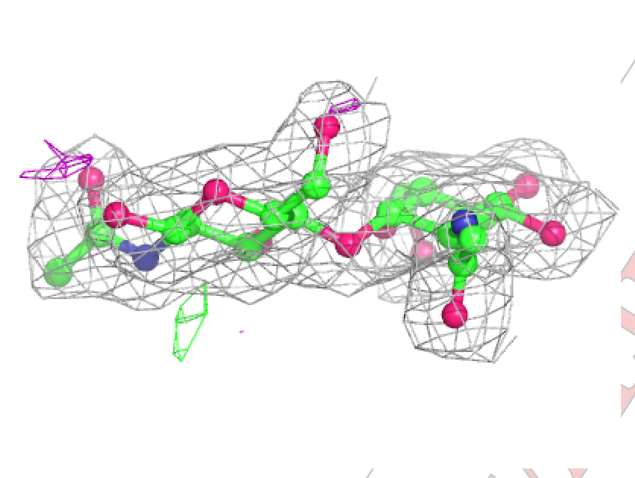

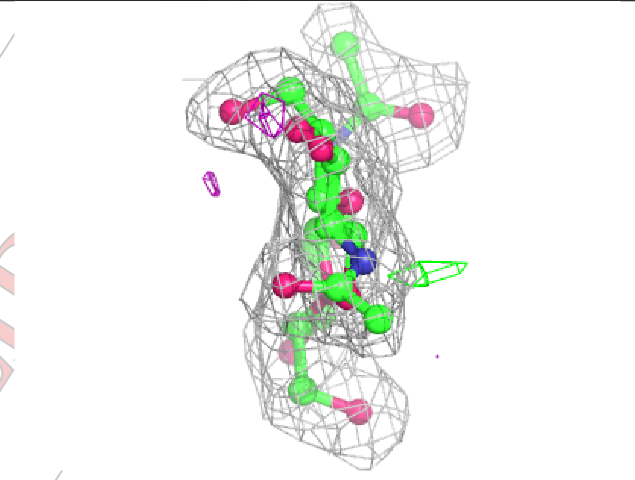

6.5 Other polymers (i)

There are no such residues in this entry.

CONFIDENTIAL VALIDATION REPORT

Full wwPDB X-ray Structure Validation Report (i)

Feb 27, 2020 – 04:21 PM JST

PDB ID	:	6LZT
Title	:	Chitin-specific solute binding protein from Vibrio harveyi co-crystalized with chitotetraose.
Deposited on	:	2020-02-19
Resolution	:	1.85 Å(reported)

This is a Full wwPDB X-ray Structure Validation Report.

This report is produced by the wwPDB biocuration pipeline after annotation of the structure.

We welcome your comments at *validation@mail.wwpdb.org*
A user guide is available at
https://www.wwpdb.org/validation/2017/XrayValidationReportHelp
with specific help available everywhere you see the (i) symbol.

The following versions of software and data (see references (i)) were used in the production of this report:

MolProbity	:	4.02b-467
Mogul	:	1.8.0 (224370), CSD as540be (2019)
Xtriage (Phenix)	:	1.13
EDS	:	2.9
buster-report	:	1.1.7 (2018)
Percentile statistics	:	20171227.v01 (using entries in the PDB archive December 27th 2017)
Refmac	:	5.8.0158
CCP4	:	7.0.044 (Gargrove)
Ideal geometry (proteins)	:	Engh & Huber (2001)
Ideal geometry (DNA, RNA)	:	Parkinson et al. (1996)
Validation Pipeline (wwPDB-VP)	:	2.9

CONFIDENTIAL VALIDATION REPORT

1 Overall quality at a glance (i)

The following experimental techniques were used to determine the structure:
X-RAY DIFFRACTION

The reported resolution of this entry is 1.85 Å.

Percentile scores (ranging between 0-100) for global validation metrics of the entry are shown in the following graphic. The table shows the number of entries on which the scores are based.

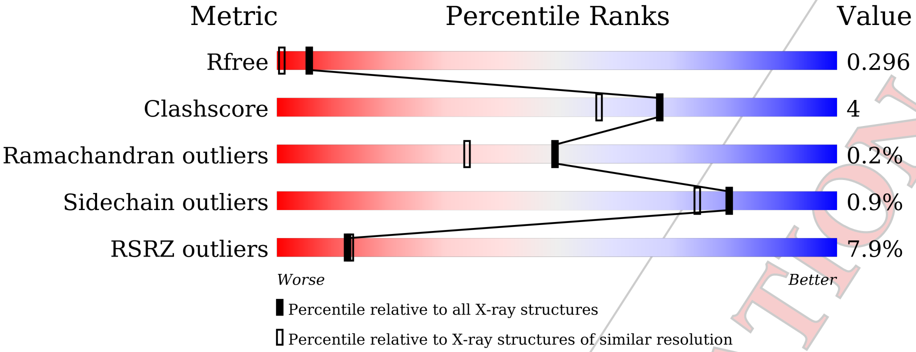

Metric	Whole archive (#Entries)	Similar resolution (#Entries, resolution range(Å))
R_{free}	111664	2111 (1.86-1.86)
Clashscore	122126	2258 (1.86-1.86)
Ramachandran outliers	120053	2234 (1.86-1.86)
Sidechain outliers	120020	2234 (1.86-1.86)
RSRZ outliers	108989	2075 (1.86-1.86)

The table below summarises the geometric issues observed across the polymeric chains and their fit to the electron density. The red, orange, yellow and green segments on the lower bar indicate the fraction of residues that contain outliers for >=3, 2, 1 and 0 types of geometric quality criteria respectively. A grey segment represents the fraction of residues that are not modelled. The numeric value for each fraction is indicated below the corresponding segment, with a dot representing fractions <=5% The upper red bar (where present) indicates the fraction of residues that have poor fit to the electron density. The numeric value is given above the bar.

Mol	Chain	Length	Quality of chain
1	A	538	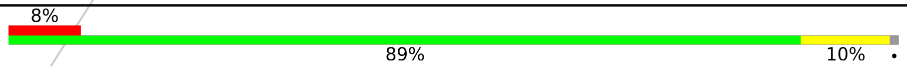

CONFIDENTIAL VALIDATION REPORT

2 Entry composition (i)

There are 5 unique types of molecules in this entry. The entry contains 4455 atoms, of which 0 are hydrogens and 0 are deuteriums.

In the tables below, the ZeroOcc column contains the number of atoms modelled with zero occupancy, the AltConf column contains the number of residues with at least one atom in alternate conformation and the Trace column contains the number of residues modelled with at most 2 atoms.

- Molecule 1 is a protein called Peptide ABC transporter, periplasmic peptide-binding protein.

Mol	Chain	Residues	Atoms					ZeroOcc	AltConf	Trace
			Total	C	N	O	S			
1	A	530	4289	2750	713	815	11	0	6	0

There are 7 discrepancies between the modelled and reference sequences:

Chain	Residue	Modelled	Actual	Comment	Reference
A	409	ALA	ASN	engineered mutation	UNP D0XC84
A	531	HIS	-	expression tag	UNP D0XC84
A	532	HIS	-	expression tag	UNP D0XC84
A	533	HIS	-	expression tag	UNP D0XC84
A	534	HIS	-	expression tag	UNP D0XC84
A	535	HIS	-	expression tag	UNP D0XC84
A	536	HIS	-	expression tag	UNP D0XC84

- Molecule 2 is DI(N-ACETYL-D-GLUCOSAMINE) (three-letter code: CBS) (formula: $C_{16}H_{28}N_2O_{11}$) (labeled as "Ligand of Interest" by author).

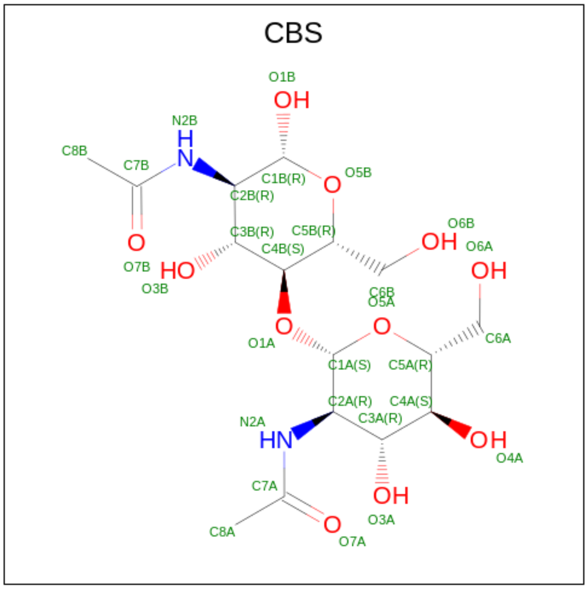

Mol	Chain	Residues	Atoms	ZeroOcc	AltConf
2	A	1	Total C N O<br>29 16 2 11	0	0

- Molecule 3 is MAGNESIUM ION (three-letter code: MG) (formula: Mg).

Mol	Chain	Residues	Atoms	ZeroOcc	AltConf
3	A	1	Total Mg<br>1 1	0	0

- Molecule 4 is CALCIUM ION (three-letter code: CA) (formula: Ca).

Mol	Chain	Residues	Atoms	ZeroOcc	AltConf
4	A	1	Total Ca<br>1 1	0	0

- Molecule 5 is water.

Mol	Chain	Residues	Atoms	ZeroOcc	AltConf
5	A	135	Total O<br>135 135	0	0

CONFIDENTIAL VALIDATION REPORT

3 Residue-property plots (i)

These plots are drawn for all protein, RNA and DNA chains in the entry. The first graphic for a chain summarises the proportions of the various outlier classes displayed in the second graphic. The second graphic shows the sequence view annotated by issues in geometry and electron density. Residues are color-coded according to the number of geometric quality criteria for which they contain at least one outlier: green = 0, yellow = 1, orange = 2 and red = 3 or more. A red dot above a residue indicates a poor fit to the electron density (RSRZ > 2). Stretches of 2 or more consecutive residues without any outlier are shown as a green connector. Residues present in the sample, but not in the model, are shown in grey.

- Molecule 1: Peptide ABC transporter, periplasmic peptide-binding protein

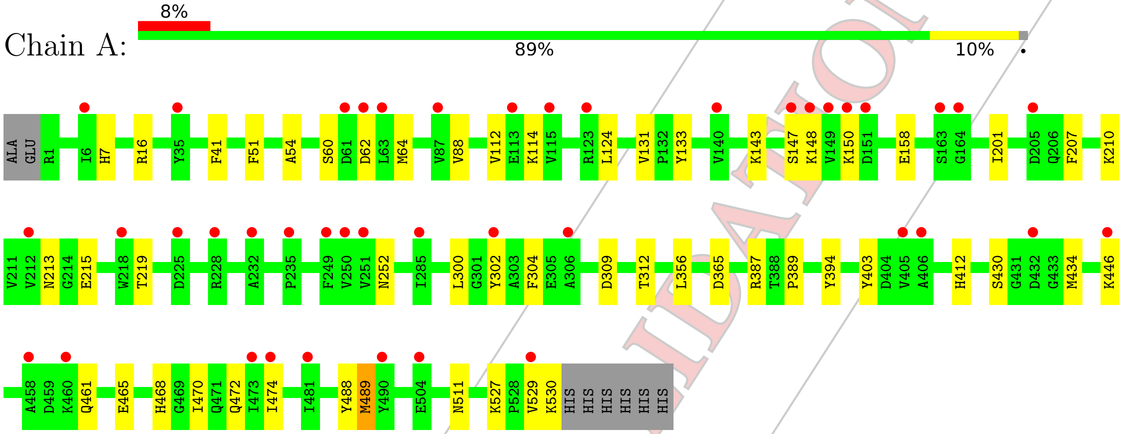

CONFIDENTIAL VALIDATION REPORT

4 Data and refinement statistics (i)

Property	Value	Source
Space group	P 1 21 1	Depositor
Cell constants<br>a, b, c, α, β, γ	59.47Å 56.44Å 80.88Å<br>90.00° 100.98° 90.00°	Depositor
Resolution (Å)	23.65 – 1.85<br>23.65 – 1.85	Depositor<br>EDS
% Data completeness<br>(in resolution range)	98.6 (23.65-1.85)<br>98.6 (23.65-1.85)	Depositor<br>EDS
R_{merge}	0.11	Depositor
R_{sym}	0.13	Depositor
$< I/\sigma(I) >$ [1]	2.36 (at 1.85Å)	Xtriage
Refinement program	PHENIX 1.9_1692	Depositor
R, R_{free}	0.235 , 0.296<br>0.237 , 0.296	Depositor<br>DCC
R_{free} test set	2236 reflections (5.05%)	wwPDB-VP
Wilson B-factor (Å^2)	28.8	Xtriage
Anisotropy	0.375	Xtriage
Bulk solvent k_{sol}(e/Å^3), B_{sol}(Å^2)	0.36 , 53.2	EDS
L-test for twinning[2]	$< \|L\| > = 0.46$, $< L^2 > = 0.29$	Xtriage
Estimated twinning fraction	No twinning to report.	Xtriage
F_o,F_c correlation	0.94	EDS
Total number of atoms	4455	wwPDB-VP
Average B, all atoms (Å^2)	36.0	wwPDB-VP

Xtriage's analysis on translational NCS is as follows: *The largest off-origin peak in the Patterson function is 12.22% of the height of the origin peak. No significant pseudotranslation is detected.*

[1] Intensities estimated from amplitudes.

[2] Theoretical values of $< |L| >$, $< L^2 >$ for acentric reflections are 0.5, 0.333 respectively for untwinned datasets, and 0.375, 0.2 for perfectly twinned datasets.

CONFIDENTIAL VALIDATION REPORT

5 Model quality (i)

5.1 Standard geometry (i)

Bond lengths and bond angles in the following residue types are not validated in this section: CBS, CA, MG

The Z score for a bond length (or angle) is the number of standard deviations the observed value is removed from the expected value. A bond length (or angle) with $|Z| > 5$ is considered an outlier worth inspection. RMSZ is the root-mean-square of all Z scores of the bond lengths (or angles).

Mol	Chain	Bond lengths		Bond angles	
		RMSZ	$\#\|Z\| > 5$	RMSZ	$\#\|Z\| > 5$
1	A	0.41	0/4417	0.55	0/6012

There are no bond length outliers.

There are no bond angle outliers.

There are no chirality outliers.

There are no planarity outliers.

5.2 Too-close contacts (i)

In the following table, the Non-H and H(model) columns list the number of non-hydrogen atoms and hydrogen atoms in the chain respectively. The H(added) column lists the number of hydrogen atoms added and optimized by MolProbity. The Clashes column lists the number of clashes within the asymmetric unit, whereas Symm-Clashes lists symmetry related clashes.

Mol	Chain	Non-H	H(model)	H(added)	Clashes	Symm-Clashes
1	A	4289	0	4095	35	0
2	A	29	0	28	0	0
3	A	1	0	0	0	0
4	A	1	0	0	0	0
5	A	135	0	0	1	0
All	All	4455	0	4123	35	0

The all-atom clashscore is defined as the number of clashes found per 1000 atoms (including hydrogen atoms). The all-atom clashscore for this structure is 4.

All (35) close contacts within the same asymmetric unit are listed below, sorted by their clash magnitude.

CONFIDENTIAL VALIDATION REPORT

Atom-1	Atom-2	Interatomic distance (Å)	Clash overlap (Å)
1:A:88:VAL:HG21	1:A:114:LYS:HB2	1.79	0.64
1:A:461:GLN:O	1:A:465:GLU:HG2	2.05	0.57
1:A:124:LEU:HD11	1:A:131:VAL:HG11	1.88	0.55
1:A:529:VAL:HG22	1:A:530[B]:LYS:HD3	1.87	0.55
1:A:529:VAL:HG22	1:A:530[A]:LYS:H	1.72	0.54
1:A:60:SER:HB2	1:A:62:ASP:OD1	2.08	0.52
1:A:389:PRO:HG2	1:A:394:TYR:HB2	1.91	0.51
1:A:7:HIS:HB3	1:A:219:THR:HG22	1.93	0.49
1:A:300:LEU:HD11	1:A:304:PHE:O	2.13	0.49
1:A:529:VAL:HG22	1:A:530[B]:LYS:H	1.78	0.49
1:A:62:ASP:O	1:A:64:MET:HG2	2.14	0.48
1:A:16:ARG:HG2	1:A:158:GLU:HB3	1.95	0.48
1:A:213:ASN:OD1	5:A:701:HOH:O	2.20	0.48
1:A:312:THR:OG1	1:A:472:GLN:HG2	2.14	0.48
1:A:7:HIS:CE1	1:A:201:ILE:HB	2.49	0.47
1:A:489:MET:HG3	1:A:489:MET:H	1.43	0.47
1:A:430:SER:O	1:A:434:MET:HG3	2.14	0.47
1:A:207:PHE:HE1	1:A:219:THR:HG21	1.80	0.46
1:A:530[B]:LYS:HE2	1:A:530[B]:LYS:HB2	1.70	0.46
1:A:412:HIS:HB3	1:A:488:TYR:OH	2.16	0.46
1:A:470:ILE:O	1:A:474[A]:ILE:HG12	2.16	0.45
1:A:210[A]:LYS:NZ	1:A:215:GLU:OE2	2.41	0.44
1:A:365:ASP:OD1	1:A:365:ASP:N	2.51	0.44
1:A:252:ASN:HB2	1:A:403:TYR:CZ	2.53	0.43
1:A:60:SER:HB2	1:A:62:ASP:CG	2.39	0.43
1:A:309:ASP:HB2	1:A:468:HIS:CG	2.53	0.42
1:A:446:LYS:H	1:A:446:LYS:HG2	1.70	0.42
1:A:530[B]:LYS:HD3	1:A:530[B]:LYS:H	1.85	0.42
1:A:51:PHE:HB3	1:A:54:ALA:O	2.20	0.42
1:A:41:PHE:CE1	1:A:133:TYR:HA	2.55	0.42
1:A:150:LYS:HD2	1:A:150:LYS:HA	1.57	0.42
1:A:488:TYR:HB3	1:A:511:ASN:HD21	1.85	0.41
1:A:356:LEU:HD13	1:A:387:ARG:CZ	2.51	0.41
1:A:143:LYS:HB3	1:A:143:LYS:HE2	1.75	0.40
1:A:88:VAL:HA	1:A:112:VAL:HG21	2.04	0.40

There are no symmetry-related clashes.

CONFIDENTIAL VALIDATION REPORT

5.3 Torsion angles (i)

5.3.1 Protein backbone (i)

In the following table, the Percentiles column shows the percent Ramachandran outliers of the chain as a percentile score with respect to all X-ray entries followed by that with respect to entries of similar resolution.

The Analysed column shows the number of residues for which the backbone conformation was analysed, and the total number of residues.

Mol	Chain	Analysed	Favoured	Allowed	Outliers	Percentiles
1	A	533/538 (99%)	508 (95%)	24 (4%)	1 (0%)	49 34

All (1) Ramachandran outliers are listed below:

Mol	Chain	Res	Type
1	A	302	TYR

5.3.2 Protein sidechains (i)

In the following table, the Percentiles column shows the percent sidechain outliers of the chain as a percentile score with respect to all X-ray entries followed by that with respect to entries of similar resolution.

The Analysed column shows the number of residues for which the sidechain conformation was analysed, and the total number of residues.

Mol	Chain	Analysed	Rotameric	Outliers	Percentiles
1	A	460/461 (100%)	456 (99%)	4 (1%)	81 75

All (4) residues with a non-rotameric sidechain are listed below:

Mol	Chain	Res	Type
1	A	147	SER
1	A	148	LYS
1	A	489	MET
1	A	527	LYS

Some sidechains can be flipped to improve hydrogen bonding and reduce clashes. There are no such sidechains identified.

CONFIDENTIAL VALIDATION REPORT

5.3.3 RNA (i)

There are no RNA molecules in this entry.

5.4 Non-standard residues in protein, DNA, RNA chains (i)

There are no non-standard protein/DNA/RNA residues in this entry.

5.5 Carbohydrates (i)

There are no carbohydrates in this entry.

5.6 Ligand geometry (i)

Of 3 ligands modelled in this entry, 2 are monoatomic - leaving 1 for Mogul analysis.

In the following table, the Counts columns list the number of bonds (or angles) for which Mogul statistics could be retrieved, the number of bonds (or angles) that are observed in the model and the number of bonds (or angles) that are defined in the Chemical Component Dictionary. The Link column lists molecule types, if any, to which the group is linked. The Z score for a bond length (or angle) is the number of standard deviations the observed value is removed from the expected value. A bond length (or angle) with $|Z| > 2$ is considered an outlier worth inspection. RMSZ is the root-mean-square of all Z scores of the bond lengths (or angles).

Mol	Type	Chain	Res	Link	Bond lengths			Bond angles		
					Counts	RMSZ	$\#\|Z\| > 2$	Counts	RMSZ	$\#\|Z\| > 2$
2	CBS	A	601	-	30,30,30	0.47	0	43,43,43	1.14	5 (11%)

In the following table, the Chirals column lists the number of chiral outliers, the number of chiral centers analysed, the number of these observed in the model and the number defined in the Chemical Component Dictionary. Similar counts are reported in the Torsion and Rings columns. '-' means no outliers of that kind were identified.

Mol	Type	Chain	Res	Link	Chirals	Torsions	Rings
2	CBS	A	601	-	-	0/16/56/56	0/2/2/2

There are no bond length outliers.

All (5) bond angle outliers are listed below:

Mol	Chain	Res	Type	Atoms	Z	Observed(^{o})	Ideal(^{o})
2	A	601	CBS	C1B-O5B-C5B	-2.64	108.63	113.68
2	A	601	CBS	O1B-C1B-C2B	2.58	114.58	109.22

Continued on next page...

CONFIDENTIAL VALIDATION REPORT

Continued from previous page...

Mol	Chain	Res	Type	Atoms	Z	Observed(^{o})	Ideal(^{o})
2	A	601	CBS	O3A-C3A-C4A	-2.18	105.28	110.34
2	A	601	CBS	C1B-C2B-C3B	-2.14	107.63	110.54
2	A	601	CBS	C1A-O1A-C4B	-2.11	112.70	117.97

There are no chirality outliers.

There are no torsion outliers.

There are no ring outliers.

No monomer is involved in short contacts.

The following is a two-dimensional graphical depiction of Mogul quality analysis of bond lengths, bond angles, torsion angles, and ring geometry for all instances of the Ligand of Interest. In addition, ligands with molecular weight > 250 and outliers as shown on the validation Tables will also be included. For torsion angles, if less then 5% of the Mogul distribution of torsion angles is within 10 degrees of the torsion angle in question, then that torsion angle is considered an outlier. Any bond that is central to one or more torsion angles identified as an outlier by Mogul will be highlighted in the graph. For rings, the root-mean-square deviation (RMSD) between the ring in question and similar rings identified by Mogul is calculated over all ring torsion angles. If the average RMSD is greater than 60 degrees and the minimal RMSD between the ring in question and any Mogul-identified rings is also greater than 60 degrees, then that ring is considered an outlier. The outliers are highlighted in purple. The color gray indicates Mogul did not find sufficient equivalents in the CSD to analyse the geometry.

CONFIDENTIAL VALIDATION REPORT

Ligand CBS A 601

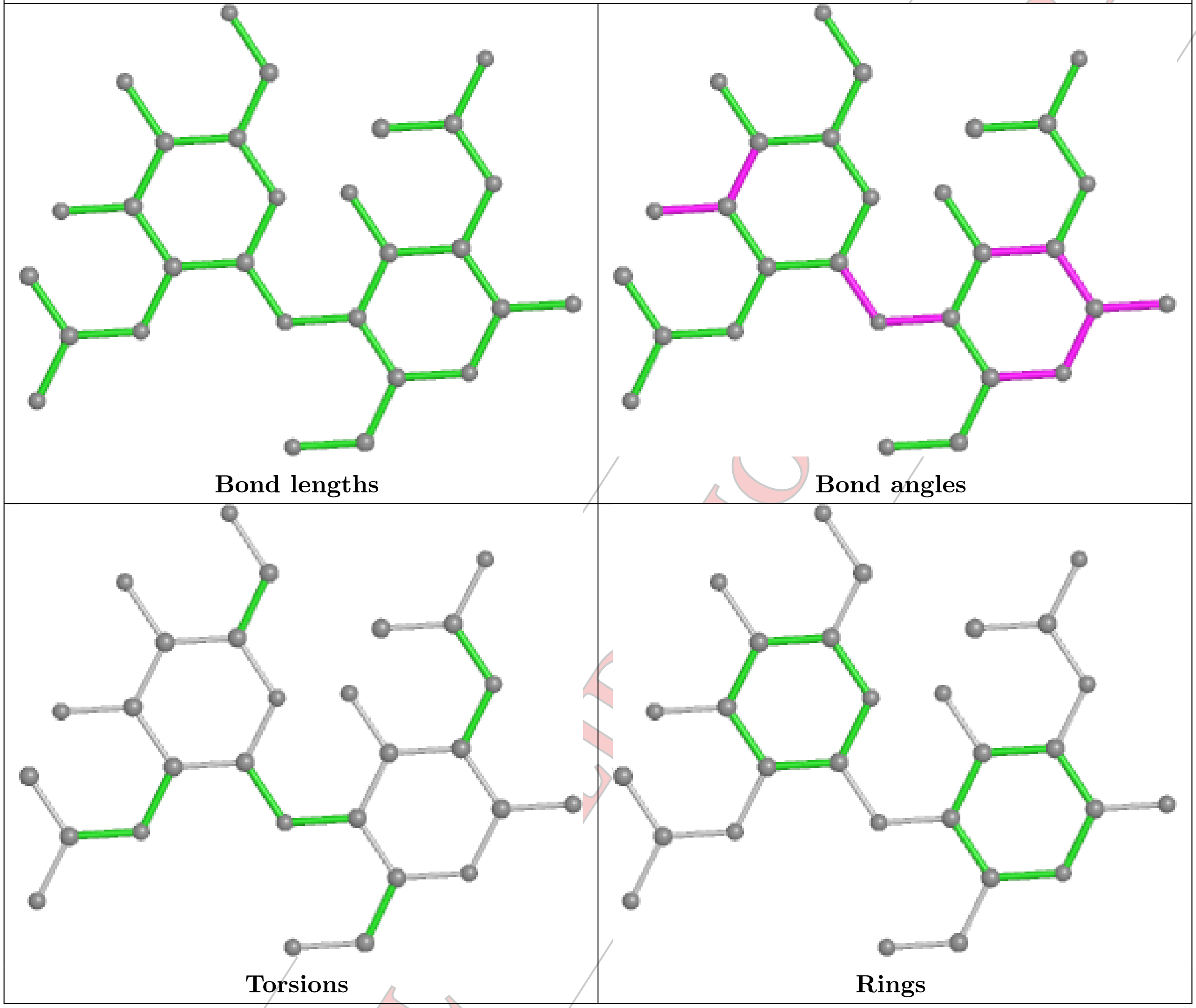

5.7 Other polymers (i)

There are no such residues in this entry.

5.8 Polymer linkage issues (i)

There are no chain breaks in this entry.

CONFIDENTIAL

6 Fit of model and data (i)

6.1 Protein, DNA and RNA chains (i)

In the following table, the column labelled '#RSRZ> 2' contains the number (and percentage) of RSRZ outliers, followed by percent RSRZ outliers for the chain as percentile scores relative to all X-ray entries and entries of similar resolution. The OWAB column contains the minimum, median, 95th percentile and maximum values of the occupancy-weighted average B-factor per residue. The column labelled 'Q< 0.9' lists the number of (and percentage) of residues with an average occupancy less than 0.9.

Mol	Chain	Analysed	<RSRZ>	#RSRZ>2			OWAB(Å^2)	Q<0.9
1	A	530/538 (98%)	0.65	42 (7%)	12	13	20, 35, 55, 76	0

All (42) RSRZ outliers are listed below:

Mol	Chain	Res	Type	RSRZ
1	A	232	ALA	5.2
1	A	529	VAL	5.1
1	A	149	VAL	5.0
1	A	302	TYR	4.8
1	A	147	SER	3.7
1	A	150	LYS	3.7
1	A	458	ALA	3.6
1	A	61	ASP	3.4
1	A	62	ASP	3.3
1	A	123	ARG	3.3
1	A	251	VAL	3.1
1	A	115	VAL	3.0
1	A	205	ASP	2.9
1	A	481	ILE	2.9
1	A	250	VAL	2.8
1	A	63	LEU	2.8
1	A	151	ASP	2.7
1	A	306	ALA	2.7
1	A	460	LYS	2.7
1	A	113	GLU	2.7
1	A	218	TRP	2.5
1	A	148	LYS	2.5
1	A	504	GLU	2.5
1	A	249	PHE	2.4
1	A	212	VAL	2.4
1	A	87	VAL	2.4
1	A	228[A]	ARG	2.3

Continued on next page...

CONFIDENTIAL VALIDATION REPORT

Continued from previous page...

Mol	Chain	Res	Type	RSRZ
1	A	235	PRO	2.3
1	A	225	ASP	2.2
1	A	490	TYR	2.2
1	A	405	VAL	2.2
1	A	140	VAL	2.2
1	A	432	ASP	2.2
1	A	285	ILE	2.1
1	A	163	SER	2.1
1	A	473	ILE	2.1
1	A	35	TYR	2.1
1	A	446	LYS	2.1
1	A	474[A]	ILE	2.1
1	A	6	ILE	2.0
1	A	164	GLY	2.0
1	A	406	ALA	2.0

6.2 Non-standard residues in protein, DNA, RNA chains (i)

There are no non-standard protein/DNA/RNA residues in this entry.

6.3 Carbohydrates (i)

There are no carbohydrates in this entry.

6.4 Ligands (i)

In the following table, the Atoms column lists the number of modelled atoms in the group and the number defined in the chemical component dictionary. The B-factors column lists the minimum, median, 95th percentile and maximum values of B factors of atoms in the group. The column labelled 'Q< 0.9' lists the number of atoms with occupancy less than 0.9.

Mol	Type	Chain	Res	Atoms	RSCC	RSR	B-factors($Å^2$)	Q<0.9
3	MG	A	602	1/1	0.89	0.09	22,22,22,22	0
2	CBS	A	601	29/29	0.94	0.10	17,24,28,30	0
4	CA	A	603	1/1	0.98	0.05	31,31,31,31	0

The following is a graphical depiction of the model fit to experimental electron density of all instances of the Ligand of Interest. In addition, ligands with molecular weight > 250 and outliers as shown on the geometry validation Tables will also be included. Each fit is shown from different orientation to approximate a three-dimensional view.

CONFIDENTIAL VALIDATION REPORT

Electron density around CBS A 601:

2mF$_o$-DF$_c$ (at 0.7 rmsd) in gray
mF$_o$-DF$_c$ (at 3 rmsd) in purple (negative)
and green (positive)

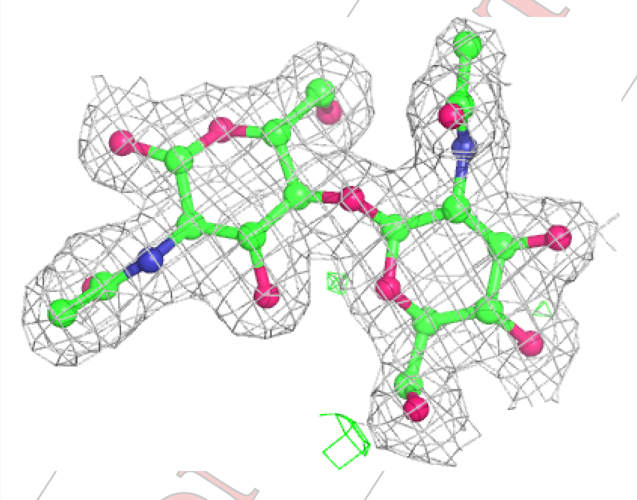

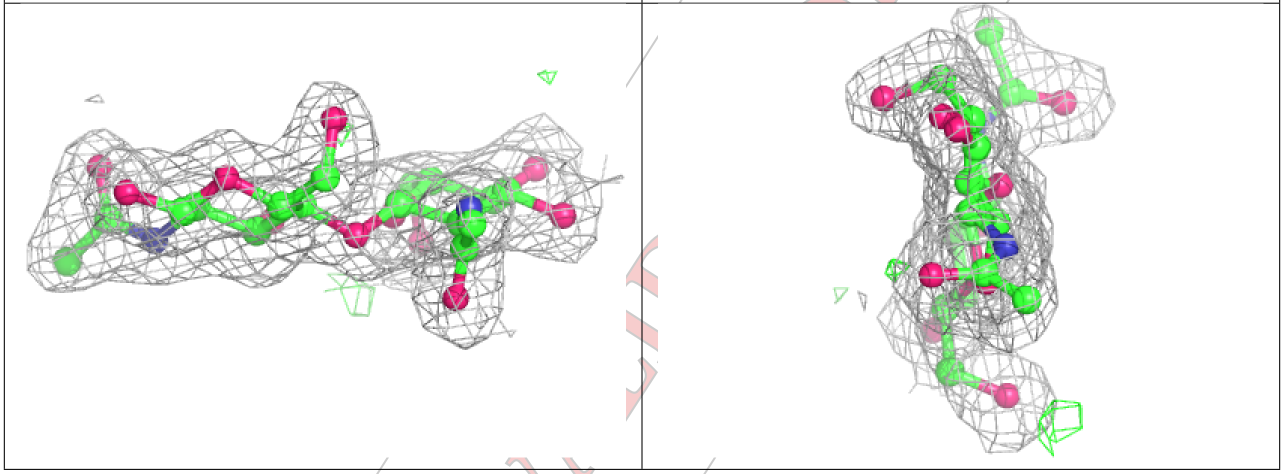

6.5 Other polymers ⓘ

There are no such residues in this entry.

CONFIDENTIAL

CONFIDENTIAL VALIDATION REPORT

Full wwPDB X-ray Structure Validation Report (i)

Mar 16, 2021 – 04:50 PM JST

PDB ID	:	7EBM
Title	:	W363A mutant of Chitin-specific solute binding protein from Vibrio harveyi in complex with chitobiose.
Deposited on	:	2021-03-10
Resolution	:	1.90 Å(reported)

This is a Full wwPDB X-ray Structure Validation Report.

This report is produced by the wwPDB biocuration pipeline after annotation of the structure.

We welcome your comments at *validation@mail.wwpdb.org*
A user guide is available at
https://www.wwpdb.org/validation/2017/XrayValidationReportHelp
with specific help available everywhere you see the (i) symbol.

The following versions of software and data (see references (i)) were used in the production of this report:

MolProbity	:	4.02b-467
Mogul	:	1.8.5 (274361), CSD as541be (2020)
Xtriage (Phenix)	:	1.13
EDS	:	2.17.1
Percentile statistics	:	20191225.v01 (using entries in the PDB archive December 25th 2019)
Refmac	:	5.8.0158
CCP4	:	7.0.044 (Gargrove)
Ideal geometry (proteins)	:	Engh & Huber (2001)
Ideal geometry (DNA, RNA)	:	Parkinson et al. (1996)
Validation Pipeline (wwPDB-VP)	:	2.17.1

1 Overall quality at a glance (i)

The following experimental techniques were used to determine the structure:
X-RAY DIFFRACTION

The reported resolution of this entry is 1.90 Å.

Percentile scores (ranging between 0-100) for global validation metrics of the entry are shown in the following graphic. The table shows the number of entries on which the scores are based.

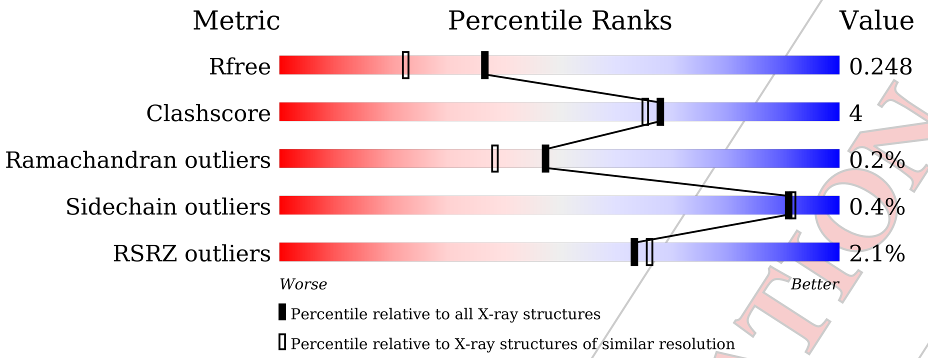

Metric	Whole archive (#Entries)	Similar resolution (#Entries, resolution range(Å))
R_{free}	130704	6207 (1.90-1.90)
Clashscore	141614	6847 (1.90-1.90)
Ramachandran outliers	138981	6760 (1.90-1.90)
Sidechain outliers	138945	6760 (1.90-1.90)
RSRZ outliers	127900	6082 (1.90-1.90)

The table below summarises the geometric issues observed across the polymeric chains and their fit to the electron density. The red, orange, yellow and green segments of the lower bar indicate the fraction of residues that contain outliers for >=3, 2, 1 and 0 types of geometric quality criteria respectively. A grey segment represents the fraction of residues that are not modelled. The numeric value for each fraction is indicated below the corresponding segment, with a dot representing fractions <=5% The upper red bar (where present) indicates the fraction of residues that have poor fit to the electron density. The numeric value is given above the bar.

Mol	Chain	Length	Quality of chain
1	A	540	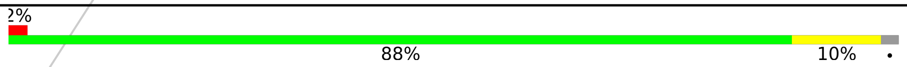 2% / 88% 10% •
2	E	2	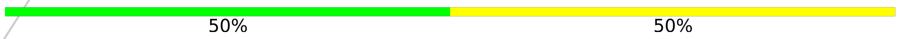 50% 50%

CONFIDENTIAL VALIDATION REPORT

2 Entry composition (i)

There are 7 unique types of molecules in this entry. The entry contains 4548 atoms, of which 0 are hydrogens and 0 are deuteriums.

In the tables below, the ZeroOcc column contains the number of atoms modelled with zero occupancy, the AltConf column contains the number of residues with at least one atom in alternate conformation and the Trace column contains the number of residues modelled with at most 2 atoms.

- Molecule 1 is a protein called Peptide ABC transporter, periplasmic peptide-binding protein.

Mol	Chain	Residues	Atoms					ZeroOcc	AltConf	Trace
			Total	C	N	O	S			
1	A	530	4250	2718	705	814	13	0	3	0

There are 9 discrepancies between the modelled and reference sequences:

Chain	Residue	Modelled	Actual	Comment	Reference
A	363	ALA	TRP	engineered mutation	UNP D0XC84
A	531	LEU	-	expression tag	UNP D0XC84
A	532	GLU	-	expression tag	UNP D0XC84
A	533	HIS	-	expression tag	UNP D0XC84
A	534	HIS	-	expression tag	UNP D0XC84
A	535	HIS	-	expression tag	UNP D0XC84
A	536	HIS	-	expression tag	UNP D0XC84
A	537	HIS	-	expression tag	UNP D0XC84
A	538	HIS	-	expression tag	UNP D0XC84

- Molecule 2 is an oligosaccharide called 2-acetamido-2-deoxy-beta-D-glucopyranose-(1-4)-2-acetamido-2-deoxy-beta-D-glucopyranose.

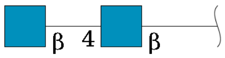

Mol	Chain	Residues	Atoms				ZeroOcc	AltConf	Trace
			Total	C	N	O			
2	E	2	29	16	2	11	0	0	0

- Molecule 3 is 1,2-ETHANEDIOL (three-letter code: EDO) (formula: $C_2H_6O_2$).

CONFIDENTIAL VALIDATION REPORT

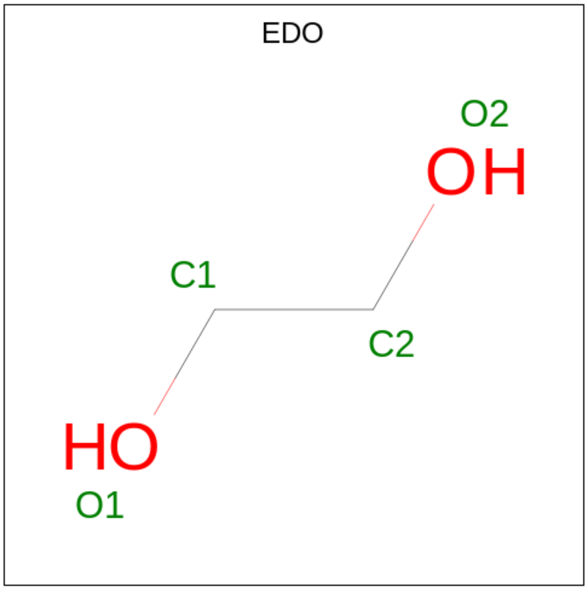

Mol	Chain	Residues	Atoms	ZeroOcc	AltConf
3	A	1	Total C O<br>4 2 2	0	0

- Molecule 4 is CALCIUM ION (three-letter code: CA) (formula: Ca).

Mol	Chain	Residues	Atoms	ZeroOcc	AltConf
4	A	2	Total Ca<br>2 2	0	0

- Molecule 5 is CHLORIDE ION (three-letter code: CL) (formula: Cl).

Mol	Chain	Residues	Atoms	ZeroOcc	AltConf
5	A	1	Total Cl<br>1 1	0	0

- Molecule 6 is MAGNESIUM ION (three-letter code: MG) (formula: Mg).

Mol	Chain	Residues	Atoms	ZeroOcc	AltConf
6	A	1	Total Mg<br>1 1	0	0

- Molecule 7 is water.

Mol	Chain	Residues	Atoms	ZeroOcc	AltConf
7	A	261	Total O<br>261 261	0	0

CONFIDENTIAL VALIDATION REPORT

3 Residue-property plots (i)

These plots are drawn for all protein, RNA, DNA and oligosaccharide chains in the entry. The first graphic for a chain summarises the proportions of the various outlier classes displayed in the second graphic. The second graphic shows the sequence view annotated by issues in geometry and electron density. Residues are color-coded according to the number of geometric quality criteria for which they contain at least one outlier: green = 0, yellow = 1, orange = 2 and red = 3 or more. A red dot above a residue indicates a poor fit to the electron density (RSRZ > 2). Stretches of 2 or more consecutive residues without any outlier are shown as a green connector. Residues present in the sample, but not in the model, are shown in grey.

- Molecule 1: Peptide ABC transporter, periplasmic peptide-binding protein

Chain A:

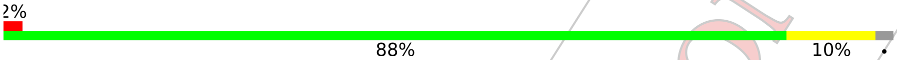

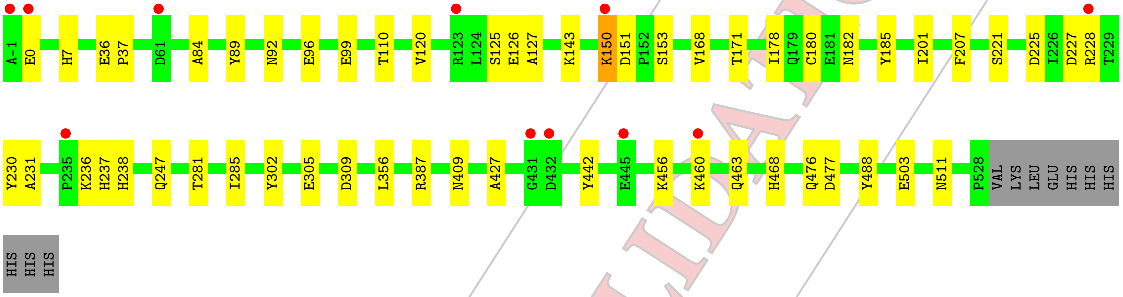

- Molecule 2: 2-acetamido-2-deoxy-beta-D-glucopyranose-(1-4)-2-acetamido-2-deoxy-beta-D-glucopyranose

Chain E:

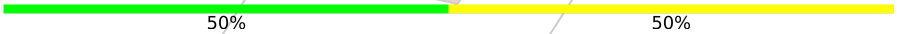

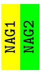

CONFIDENTIAL VALIDATION REPORT

4 Data and refinement statistics (i)

Property	Value	Source
Space group	P 1 21 1	Depositor
Cell constants<br>a, b, c, α, β, γ	58.57Å 55.99Å 80.32Å<br>90.00° 101.84° 90.00°	Depositor
Resolution (Å)	23.73 – 1.90<br>23.73 – 1.89	Depositor<br>EDS
% Data completeness<br>(in resolution range)	94.2 (23.73-1.90)<br>94.2 (23.73-1.89)	Depositor<br>EDS
R_{merge}	(Not available)	Depositor
R_{sym}	(Not available)	Depositor
$< I/\sigma(I) >$ [1]	2.46 (at 1.89Å)	Xtriage
Refinement program	PHENIX 1.9_1692	Depositor
R, R_{free}	0.183 , 0.249<br>0.184 , 0.248	Depositor<br>DCC
R_{free} test set	1914 reflections (4.94%)	wwPDB-VP
Wilson B-factor (Å^2)	19.5	Xtriage
Anisotropy	0.097	Xtriage
Bulk solvent k_{sol}(e/Å^3), B_{sol}(Å^2)	0.38 , 53.9	EDS
L-test for twinning[2]	$< \|L\| > = 0.48$, $< L^2 > = 0.31$	Xtriage
Estimated twinning fraction	No twinning to report.	Xtriage
F_o,F_c correlation	0.95	EDS
Total number of atoms	4548	wwPDB-VP
Average B, all atoms (Å^2)	21.0	wwPDB-VP

Xtriage's analysis on translational NCS is as follows: *The largest off-origin peak in the Patterson function is 6.55% of the height of the origin peak. No significant pseudotranslation is detected.*

[1] Intensities estimated from amplitudes.
[2] Theoretical values of $< |L| >$, $< L^2 >$ for acentric reflections are 0.5, 0.333 respectively for untwinned datasets, and 0.375, 0.2 for perfectly twinned datasets.

CONFIDENTIAL VALIDATION REPORT

5 Model quality (i)

5.1 Standard geometry (i)

Bond lengths and bond angles in the following residue types are not validated in this section: NAG, CA, EDO, MG, CL

The Z score for a bond length (or angle) is the number of standard deviations the observed value is removed from the expected value. A bond length (or angle) with $|Z| > 5$ is considered an outlier worth inspection. RMSZ is the root-mean-square of all Z scores of the bond lengths (or angles).

Mol	Chain	Bond lengths		Bond angles	
		RMSZ	$\#|Z|>5$	RMSZ	$\#|Z|>5$
1	A	0.41	0/4374	0.53	0/5958

There are no bond length outliers.

There are no bond angle outliers.

There are no chirality outliers.

There are no planarity outliers.

5.2 Too-close contacts (i)

In the following table, the Non-H and H(model) columns list the number of non-hydrogen atoms and hydrogen atoms in the chain respectively. The H(added) column lists the number of hydrogen atoms added and optimized by MolProbity. The Clashes column lists the number of clashes within the asymmetric unit, whereas Symm-Clashes lists symmetry-related clashes.

Mol	Chain	Non-H	H(model)	H(added)	Clashes	Symm-Clashes
1	A	4250	0	4035	32	0
2	E	29	0	27	0	0
3	A	4	0	6	0	0
4	A	2	0	0	0	0
5	A	1	0	0	0	0
6	A	1	0	0	0	0
7	A	261	0	0	5	0
All	All	4548	0	4068	32	0

The all-atom clashscore is defined as the number of clashes found per 1000 atoms (including hydrogen atoms). The all-atom clashscore for this structure is 4.

All (32) close contacts within the same asymmetric unit are listed below, sorted by their clash magnitude.

CONFIDENTIAL VALIDATION REPORT

Atom-1	Atom-2	Interatomic distance (Å)	Clash overlap (Å)
1:A:463:GLN:NE2	7:A:805:HOH:O	2.23	0.71
1:A:476:GLN:NE2	7:A:802:HOH:O	2.20	0.70
1:A:281:THR:HG23	1:A:285:ILE:HD13	1.77	0.67
1:A:225:ASP:OD2	1:A:228:ARG:NH2	2.28	0.66
1:A:460:LYS:NZ	7:A:811:HOH:O	2.33	0.62
1:A:127:ALA:HB3	1:A:456:LYS:HD3	1.82	0.62
1:A:89:TYR:HB2	1:A:143:LYS:HD3	1.85	0.58
1:A:227:ASP:HA	1:A:231:ALA:HB3	1.86	0.57
1:A:36:GLU:HG3	1:A:37:PRO:HD2	1.91	0.52
1:A:126:GLU:HG3	1:A:456:LYS:NZ	2.26	0.51
1:A:427:ALA:O	7:A:801:HOH:O	2.19	0.50
1:A:302:TYR:O	1:A:305:GLU:HG2	2.11	0.50
1:A:182:ASN:HB3	1:A:185:TYR:HB2	1.95	0.48
1:A:92:ASN:O	1:A:96:GLU:HG2	2.15	0.47
1:A:110:THR:HG23	1:A:125:SER:HB3	1.97	0.46
1:A:99:GLU:OE2	1:A:153:SER:HB3	2.15	0.46
1:A:171:THR:HB	1:A:178:ILE:HG13	1.98	0.45
1:A:236:LYS:HA	1:A:236:LYS:HD3	1.75	0.45
1:A:168:VAL:HB	1:A:180[B]:CYS:SG	2.57	0.44
1:A:247:GLN:HG2	1:A:409:ASN:HB3	1.99	0.44
1:A:84:ALA:HB1	1:A:120:VAL:HG22	1.99	0.43
1:A:150:LYS:H	1:A:150:LYS:HG3	1.58	0.42
1:A:7:HIS:CE1	1:A:201:ILE:HB	2.55	0.41
1:A:207:PHE:CE2	1:A:221:SER:HB2	2.55	0.41
1:A:230:TYR:CZ	1:A:237:HIS:HB3	2.54	0.41
1:A:309:ASP:HB2	1:A:468:HIS:CG	2.56	0.41
1:A:442:TYR:OH	1:A:477:ASP:OD2	2.32	0.41
1:A:356:LEU:HD13	1:A:387:ARG:CZ	2.51	0.40
1:A:150:LYS:HB2	1:A:151:ASP:H	1.51	0.40
1:A:238:HIS:ND1	7:A:804:HOH:O	2.23	0.40
1:A:511:ASN:ND2	1:A:511:ASN:H	2.20	0.40
1:A:488:TYR:HB3	1:A:511:ASN:HD21	1.85	0.40

There are no symmetry-related clashes.

5.3 Torsion angles (i)

5.3.1 Protein backbone (i)

In the following table, the Percentiles column shows the percent Ramachandran outliers of the chain as a percentile score with respect to all X-ray entries followed by that with respect to entries of similar resolution.

CONFIDENTIAL VALIDATION REPORT

The Analysed column shows the number of residues for which the backbone conformation was analysed, and the total number of residues.

Mol	Chain	Analysed	Favoured	Allowed	Outliers	Percentiles
1	A	531/540 (98%)	514 (97%)	16 (3%)	1 (0%)	47 38

All (1) Ramachandran outliers are listed below:

Mol	Chain	Res	Type
1	A	150	LYS

5.3.2 Protein sidechains (i)

In the following table, the Percentiles column shows the percent sidechain outliers of the chain as a percentile score with respect to all X-ray entries followed by that with respect to entries of similar resolution.

The Analysed column shows the number of residues for which the sidechain conformation was analysed, and the total number of residues.

Mol	Chain	Analysed	Rotameric	Outliers	Percentiles
1	A	456/463 (98%)	454 (100%)	2 (0%)	91 91

All (2) residues with a non-rotameric sidechain are listed below:

Mol	Chain	Res	Type
1	A	0	GLU
1	A	503	GLU

Sometimes sidechains can be flipped to improve hydrogen bonding and reduce clashes. There are no such sidechains identified.

5.3.3 RNA (i)

There are no RNA molecules in this entry.

5.4 Non-standard residues in protein, DNA, RNA chains (i)

There are no non-standard protein/DNA/RNA residues in this entry.

CONFIDENTIAL VALIDATION REPORT

5.5 Carbohydrates (i)

2 monosaccharides are modelled in this entry.

In the following table, the Counts columns list the number of bonds (or angles) for which Mogul statistics could be retrieved, the number of bonds (or angles) that are observed in the model and the number of bonds (or angles) that are defined in the Chemical Component Dictionary. The Link column lists molecule types, if any, to which the group is linked. The Z score for a bond length (or angle) is the number of standard deviations the observed value is removed from the expected value. A bond length (or angle) with $|Z| > 2$ is considered an outlier worth inspection. RMSZ is the root-mean-square of all Z scores of the bond lengths (or angles).

Mol	Type	Chain	Res	Link	Bond lengths			Bond angles		
					Counts	RMSZ	$\#\|Z\| > 2$	Counts	RMSZ	$\#\|Z\| > 2$
2	NAG	E	1	2	15,15,15	0.41	0	21,21,21	1.12	2 (9%)
2	NAG	E	2	2	14,14,15	0.37	0	17,19,21	0.54	0

In the following table, the Chirals column lists the number of chiral outliers, the number of chiral centers analysed, the number of these observed in the model and the number defined in the Chemical Component Dictionary. Similar counts are reported in the Torsion and Rings columns. '-' means no outliers of that kind were identified.

Mol	Type	Chain	Res	Link	Chirals	Torsions	Rings
2	NAG	E	1	2	-	0/6/26/26	0/1/1/1
2	NAG	E	2	2	-	0/6/23/26	0/1/1/1

There are no bond length outliers.

All (2) bond angle outliers are listed below:

Mol	Chain	Res	Type	Atoms	Z	Observed(^{o})	Ideal(^{o})
2	E	1	NAG	O5-C1-C2	-2.51	106.99	109.52
2	E	1	NAG	C1-C2-N2	2.43	113.54	110.73

There are no chirality outliers.

There are no torsion outliers.

There are no ring outliers.

No monomer is involved in short contacts.

5.6 Ligand geometry (i)

Of 5 ligands modelled in this entry, 4 are monoatomic - leaving 1 for Mogul analysis.

CONFIDENTIAL VALIDATION REPORT

In the following table, the Counts columns list the number of bonds (or angles) for which Mogul statistics could be retrieved, the number of bonds (or angles) that are observed in the model and the number of bonds (or angles) that are defined in the Chemical Component Dictionary. The Link column lists molecule types, if any, to which the group is linked. The Z score for a bond length (or angle) is the number of standard deviations the observed value is removed from the expected value. A bond length (or angle) with $|Z| > 2$ is considered an outlier worth inspection. RMSZ is the root-mean-square of all Z scores of the bond lengths (or angles).

Mol	Type	Chain	Res	Link	Bond lengths			Bond angles		
					Counts	RMSZ	$\#\|Z\| > 2$	Counts	RMSZ	$\#\|Z\| > 2$
3	EDO	A	701	-	3,3,3	0.45	0	2,2,2	0.61	0

In the following table, the Chirals column lists the number of chiral outliers, the number of chiral centers analysed, the number of these observed in the model and the number defined in the Chemical Component Dictionary. Similar counts are reported in the Torsion and Rings columns. '-' means no outliers of that kind were identified.

Mol	Type	Chain	Res	Link	Chirals	Torsions	Rings
3	EDO	A	701	-	-	0/1/1/1	-

There are no bond length outliers.

There are no bond angle outliers.

There are no chirality outliers.

There are no torsion outliers.

There are no ring outliers.

No monomer is involved in short contacts.

5.7 Other polymers (i)

There are no such residues in this entry.

5.8 Polymer linkage issues (i)

There are no chain breaks in this entry.

CONFIDENTIAL VALIDATION REPORT

6 Fit of model and data (i)

6.1 Protein, DNA and RNA chains (i)

In the following table, the column labelled '#RSRZ> 2' contains the number (and percentage) of RSRZ outliers, followed by percent RSRZ outliers for the chain as percentile scores relative to all X-ray entries and entries of similar resolution. The OWAB column contains the minimum, median, 95th percentile and maximum values of the occupancy-weighted average B-factor per residue. The column labelled 'Q< 0.9' lists the number of (and percentage) of residues with an average occupancy less than 0.9.

Mol	Chain	Analysed	<RSRZ>	#RSRZ>2			OWAB(Å^2)	Q<0.9
1	A	530/540 (98%)	-0.05	11 (2%)	63	66	8, 20, 37, 57	0

All (11) RSRZ outliers are listed below:

Mol	Chain	Res	Type	RSRZ
1	A	-1	ALA	7.8
1	A	432	ASP	4.6
1	A	431	GLY	3.2
1	A	0	GLU	3.0
1	A	150	LYS	2.9
1	A	61	ASP	2.8
1	A	228	ARG	2.7
1	A	460	LYS	2.4
1	A	235	PRO	2.4
1	A	123	ARG	2.1
1	A	445	GLU	2.0

6.2 Non-standard residues in protein, DNA, RNA chains (i)

There are no non-standard protein/DNA/RNA residues in this entry.

6.3 Carbohydrates (i)

In the following table, the Atoms column lists the number of modelled atoms in the group and the number defined in the chemical component dictionary. The B-factors column lists the minimum, median, 95th percentile and maximum values of B factors of atoms in the group. The column labelled 'Q< 0.9' lists the number of atoms with occupancy less than 0.9.

Mol	Type	Chain	Res	Atoms	RSCC	RSR	B-factors(Å^2)	Q<0.9
2	NAG	E	1	15/15	0.96	0.08	10,13,17,20	0

Continued on next page...

CONFIDENTIAL VALIDATION REPORT

Continued from previous page...

Mol	Type	Chain	Res	Atoms	RSCC	RSR	B-factors(Å^2)	Q<0.9
2	NAG	E	2	14/15	0.97	0.09	9,11,15,15	0

6.4 Ligands ⓘ

In the following table, the Atoms column lists the number of modelled atoms in the group and the number defined in the chemical component dictionary. The B-factors column lists the minimum, median, 95th percentile and maximum values of B factors of atoms in the group. The column labelled 'Q< 0.9' lists the number of atoms with occupancy less than 0.9.

Mol	Type	Chain	Res	Atoms	RSCC	RSR	B-factors(Å^2)	Q<0.9
3	EDO	A	701	4/4	0.78	0.17	32,35,35,37	0
4	CA	A	702	1/1	0.96	0.05	21,21,21,21	0
6	MG	A	705	1/1	0.97	0.04	18,18,18,18	0
5	CL	A	704	1/1	0.99	0.04	17,17,17,17	0
4	CA	A	703	1/1	0.99	0.02	16,16,16,16	0

6.5 Other polymers ⓘ

There are no such residues in this entry.

CONFIDENTIAL VALIDATION REPORT

Full wwPDB X-ray Structure Validation Report ⓘ

Feb 8, 2021 – 05:14 PM JST

PDB ID	:	6LZW
Title	:	W513A mutant of chitin-specific solute binding protein from Vibrio harveyi co-crystalized with chitobiose.
Deposited on	:	2020-02-19
Resolution	:	1.90 Å(reported)

This is a Full wwPDB X-ray Structure Validation Report.

This report is produced by the wwPDB biocuration pipeline after annotation of the structure.

We welcome your comments at *validation@mail.wwpdb.org*
A user guide is available at
https://www.wwpdb.org/validation/2017/XrayValidationReportHelp
with specific help available everywhere you see the ⓘ symbol.

CONFIDENTIAL VALIDATION REPORT

The following versions of software and data (see references ⓘ) were used in the production of this report:

MolProbity	:	4.02b-467
Mogul	:	1.8.5 (274361), CSD as541be (2020)
Xtriage (Phenix)	:	1.13
EDS	:	2.16
Percentile statistics	:	20191225.v01 (using entries in the PDB archive December 25th 2019)
Refmac	:	5.8.0158
CCP4	:	7.0.044 (Gargrove)
Ideal geometry (proteins)	:	Engh & Huber (2001)
Ideal geometry (DNA, RNA)	:	Parkinson et al. (1996)
Validation Pipeline (wwPDB-VP)	:	2.16

1 Overall quality at a glance (i)

The following experimental techniques were used to determine the structure:
X-RAY DIFFRACTION

The reported resolution of this entry is 1.90 Å.

Percentile scores (ranging between 0-100) for global validation metrics of the entry are shown in the following graphic. The table shows the number of entries on which the scores are based.

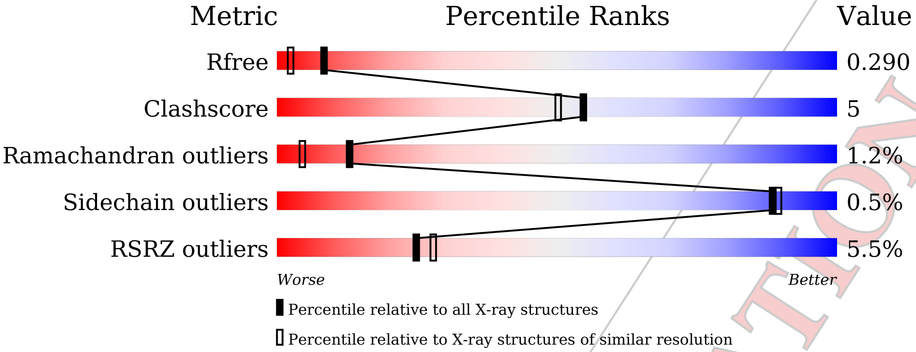

Metric	Whole archive (#Entries)	Similar resolution (#Entries, resolution range(Å))
R_{free}	130704	6207 (1.90-1.90)
Clashscore	141614	6847 (1.90-1.90)
Ramachandran outliers	138981	6760 (1.90-1.90)
Sidechain outliers	138945	6760 (1.90-1.90)
RSRZ outliers	127900	6082 (1.90-1.90)

The table below summarises the geometric issues observed across the polymeric chains and their fit to the electron density. The red, orange, yellow and green segments of the lower bar indicate the fraction of residues that contain outliers for >=3, 2, 1 and 0 types of geometric quality criteria respectively. A grey segment represents the fraction of residues that are not modelled. The numeric value for each fraction is indicated below the corresponding segment, with a dot representing fractions <=5% The upper red bar (where present) indicates the fraction of residues that have poor fit to the electron density. The numeric value is given above the bar.

Mol	Chain	Length	Quality of chain
1	A	538	5% / 85% 12% • •
2	G	2	100%

CONFIDENTIAL VALIDATION REPORT

2 Entry composition (i)

There are 7 unique types of molecules in this entry. The entry contains 4330 atoms, of which 0 are hydrogens and 0 are deuteriums.

In the tables below, the ZeroOcc column contains the number of atoms modelled with zero occupancy, the AltConf column contains the number of residues with at least one atom in alternate conformation and the Trace column contains the number of residues modelled with at most 2 atoms.

- Molecule 1 is a protein called Peptide ABC transporter, periplasmic peptide-binding protein.

Mol	Chain	Residues	Atoms					ZeroOcc	AltConf	Trace
			Total	C	N	O	S			
1	A	524	4161	2659	696	795	11	0	3	0

There are 7 discrepancies between the modelled and reference sequences:

Chain	Residue	Modelled	Actual	Comment	Reference
A	513	ALA	TRP	engineered mutation	UNP D0XC84
A	531	HIS	-	expression tag	UNP D0XC84
A	532	HIS	-	expression tag	UNP D0XC84
A	533	HIS	-	expression tag	UNP D0XC84
A	534	HIS	-	expression tag	UNP D0XC84
A	535	HIS	-	expression tag	UNP D0XC84
A	536	HIS	-	expression tag	UNP D0XC84

- Molecule 2 is an oligosaccharide called 2-acetamido-2-deoxy-beta-D-glucopyranose-(1-4)-2-acetamido-2-deoxy-beta-D-glucopyranose.

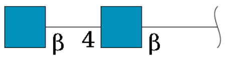

Mol	Chain	Residues	Atoms				ZeroOcc	AltConf	Trace
			Total	C	N	O			
2	G	2	29	16	2	11	0	0	0

- Molecule 3 is 1,2-ETHANEDIOL (three-letter code: EDO) (formula: $C_2H_6O_2$).

CONFIDENTIAL VALIDATION REPORT

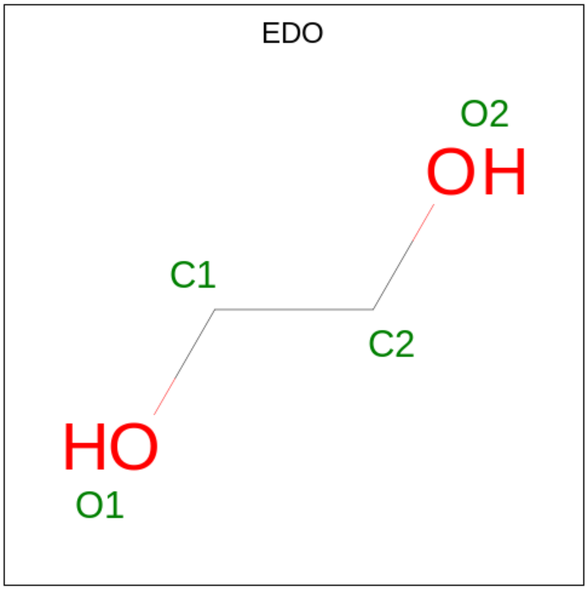

Mol	Chain	Residues	Atoms	ZeroOcc	AltConf
3	A	1	Total C O<br>4 2 2	0	0

- Molecule 4 is CALCIUM ION (three-letter code: CA) (formula: Ca).

Mol	Chain	Residues	Atoms	ZeroOcc	AltConf
4	A	1	Total Ca<br>1 1	0	0

- Molecule 5 is CHLORIDE ION (three-letter code: CL) (formula: Cl).

Mol	Chain	Residues	Atoms	ZeroOcc	AltConf
5	A	1	Total Cl<br>1 1	0	0

- Molecule 6 is MAGNESIUM ION (three-letter code: MG) (formula: Mg).

Mol	Chain	Residues	Atoms	ZeroOcc	AltConf
6	A	1	Total Mg<br>1 1	0	0

- Molecule 7 is water.

Mol	Chain	Residues	Atoms	ZeroOcc	AltConf
7	A	133	Total O<br>133 133	0	0

CONFIDENTIAL VALIDATION REPORT

3 Residue-property plots (i)

These plots are drawn for all protein, RNA, DNA and oligosaccharide chains in the entry. The first graphic for a chain summarises the proportions of the various outlier classes displayed in the second graphic. The second graphic shows the sequence view annotated by issues in geometry and electron density. Residues are color-coded according to the number of geometric quality criteria for which they contain at least one outlier: green = 0, yellow = 1, orange = 2 and red = 3 or more. A red dot above a residue indicates a poor fit to the electron density (RSRZ > 2). Stretches of 2 or more consecutive residues without any outlier are shown as a green connector. Residues present in the sample, but not in the model, are shown in grey.

• Molecule 1: Peptide ABC transporter, periplasmic peptide-binding protein

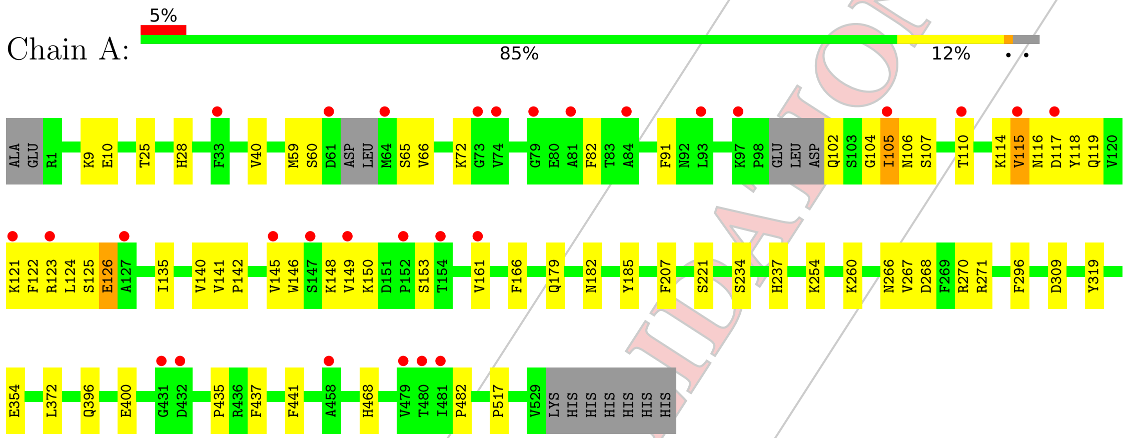

• Molecule 2: 2-acetamido-2-deoxy-beta-D-glucopyranose-(1-4)-2-acetamido-2-deoxy-beta-D-gluc opyranose

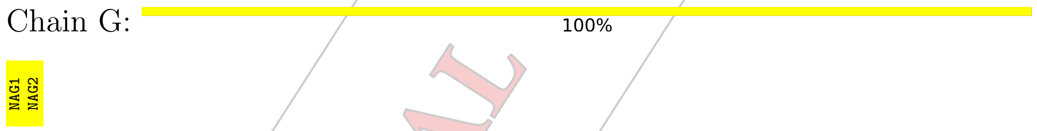

CONFIDENTIAL VALIDATION REPORT

4 Data and refinement statistics (i)

Property	Value	Source
Space group	P 1 21 1	Depositor
Cell constants<br>a, b, c, α, β, γ	61.68Å 57.11Å 81.52Å<br>90.00° 101.70° 90.00°	Depositor
Resolution (Å)	26.89 – 1.90<br>26.89 – 1.89	Depositor<br>EDS
% Data completeness<br>(in resolution range)	87.6 (26.89-1.90)<br>87.6 (26.89-1.89)	Depositor<br>EDS
R_{merge}	0.10	Depositor
R_{sym}	0.23	Depositor
$< I/\sigma(I) >$ [1]	1.28 (at 1.89Å)	Xtriage
Refinement program	PHENIX 1.9_1692	Depositor
R, R_{free}	0.227 , 0.290<br>0.228 , 0.290	Depositor<br>DCC
R_{free} test set	1990 reflections (5.06%)	wwPDB-VP
Wilson B-factor (Å^2)	27.4	Xtriage
Anisotropy	0.676	Xtriage
Bulk solvent k_{sol}(e/Å^3), B_{sol}(Å^2)	0.35 , 46.4	EDS
L-test for twinning[2]	$< \|L\| > = 0.45$, $< L^2 > = 0.28$	Xtriage
Estimated twinning fraction	No twinning to report.	Xtriage
F_o,F_c correlation	0.94	EDS
Total number of atoms	4330	wwPDB-VP
Average B, all atoms (Å^2)	34.0	wwPDB-VP

Xtriage's analysis on translational NCS is as follows: *The largest off-origin peak in the Patterson function is 5.10% of the height of the origin peak. No significant pseudotranslation is detected.*

[1] Intensities estimated from amplitudes.
[2] Theoretical values of $< |L| >$, $< L^2 >$ for acentric reflections are 0.5, 0.333 respectively for untwinned datasets, and 0.375, 0.2 for perfectly twinned datasets.

CONFIDENTIAL VALIDATION REPORT

5 Model quality (i)

5.1 Standard geometry (i)

Bond lengths and bond angles in the following residue types are not validated in this section: EDO, MG, CA, NAG, CL

The Z score for a bond length (or angle) is the number of standard deviations the observed value is removed from the expected value. A bond length (or angle) with $|Z| > 5$ is considered an outlier worth inspection. RMSZ is the root-mean-square of all Z scores of the bond lengths (or angles).

Mol	Chain	Bond lengths		Bond angles	
		RMSZ	#|Z| >5	RMSZ	#|Z| >5
1	A	0.42	0/4279	0.55	0/5836

There are no bond length outliers.

There are no bond angle outliers.

There are no chirality outliers.

There are no planarity outliers.

5.2 Too-close contacts (i)

In the following table, the Non-H and H(model) columns list the number of non-hydrogen atoms and hydrogen atoms in the chain respectively. The H(added) column lists the number of hydrogen atoms added and optimized by MolProbity. The Clashes column lists the number of clashes within the asymmetric unit, whereas Symm-Clashes lists symmetry-related clashes.

Mol	Chain	Non-H	H(model)	H(added)	Clashes	Symm-Clashes
1	A	4161	0	3890	42	0
2	G	29	0	27	0	0
3	A	4	0	6	0	0
4	A	1	0	0	0	0
5	A	1	0	0	0	0
6	A	1	0	0	0	0
7	A	133	0	0	2	0
All	All	4330	0	3923	42	0

The all-atom clashscore is defined as the number of clashes found per 1000 atoms (including hydrogen atoms). The all-atom clashscore for this structure is 5.

All (42) close contacts within the same asymmetric unit are listed below, sorted by their clash magnitude.

CONFIDENTIAL VALIDATION REPORT

Atom-1	Atom-2	Interatomic distance (Å)	Clash overlap (Å)
1:A:114:LYS:O	1:A:116:ASN:N	2.14	0.78
1:A:145:VAL:HG21	1:A:161:VAL:HG21	1.72	0.71
1:A:116:ASN:HB3	1:A:119:GLN:HB2	1.71	0.71
1:A:66:VAL:HG23	1:A:122:PHE:HB2	1.72	0.70
1:A:9:LYS:HD2	1:A:28:HIS:CE1	2.34	0.63
1:A:59[B]:MET:HG2	1:A:66:VAL:HG12	1.80	0.61
1:A:254:LYS:HE2	1:A:441:PHE:HB3	1.84	0.60
1:A:40:VAL:HG13	1:A:517:PRO:HB2	1.85	0.59
1:A:104:GLY:O	1:A:105:ILE:HB	2.04	0.58
1:A:142:PRO:HG3	1:A:161:VAL:HB	1.90	0.54
1:A:10:GLU:HG3	7:A:783:HOH:O	2.08	0.54
1:A:65:SER:HA	1:A:124:LEU:HD22	1.88	0.54
1:A:102:GLN:OE1	1:A:106:ASN:HB2	2.09	0.53
1:A:267:VAL:HG22	1:A:270:ARG:NH2	2.24	0.53
1:A:267:VAL:HG22	1:A:270:ARG:HH22	1.74	0.52
1:A:102:GLN:HE22	1:A:106:ASN:HB2	1.75	0.51
1:A:296:PHE:O	1:A:482:PRO:HG2	2.13	0.49
1:A:107:SER:OG	1:A:107:SER:O	2.27	0.48
1:A:105:ILE:HG23	1:A:107:SER:H	1.79	0.47
1:A:267:VAL:HG23	7:A:806:HOH:O	2.14	0.47
1:A:60:SER:HG	1:A:65:SER:H	1.63	0.47
1:A:115:VAL:HG11	1:A:121:LYS:HE3	1.97	0.47
1:A:117:ASP:OD1	1:A:117:ASP:N	2.43	0.46
1:A:271:ARG:HD3	1:A:319:TYR:CD1	2.50	0.46
1:A:125:SER:OG	1:A:126:GLU:N	2.49	0.46
1:A:260:LYS:HE2	1:A:354:GLU:O	2.16	0.45
1:A:110:THR:O	1:A:123:ARG:HB2	2.17	0.45
1:A:435:PRO:HB2	1:A:437:PHE:CD2	2.51	0.44
1:A:234:SER:HB3	1:A:237:HIS:CG	2.53	0.43
1:A:102:GLN:NE2	1:A:106:ASN:HB2	2.33	0.43
1:A:234:SER:HB3	1:A:237:HIS:CD2	2.54	0.43
1:A:266:ASN:OD1	1:A:268[A]:ASP:HB2	2.19	0.43
1:A:72:LYS:HE2	1:A:118:TYR:CD2	2.53	0.43
1:A:182:ASN:HB3	1:A:185:TYR:HB2	1.99	0.43
1:A:91:PHE:CZ	1:A:135:ILE:HG23	2.54	0.42
1:A:82:PHE:CZ	1:A:140:VAL:HG11	2.54	0.42
1:A:207:PHE:CE2	1:A:221:SER:HB2	2.55	0.41
1:A:396:GLN:NE2	1:A:400:GLU:OE2	2.53	0.41
1:A:309:ASP:HB2	1:A:468:HIS:CG	2.56	0.41
1:A:166:PHE:HB3	1:A:179:GLN:HG2	2.03	0.41
1:A:141:VAL:HB	1:A:146:TRP:CD1	2.56	0.41
1:A:372:LEU:HD23	1:A:372:LEU:HA	1.96	0.41

CONFIDENTIAL VALIDATION REPORT

There are no symmetry-related clashes.

5.3 Torsion angles (i)

5.3.1 Protein backbone (i)

In the following table, the Percentiles column shows the percent Ramachandran outliers of the chain as a percentile score with respect to all X-ray entries followed by that with respect to entries of similar resolution.

The Analysed column shows the number of residues for which the backbone conformation was analysed, and the total number of residues.

Mol	Chain	Analysed	Favoured	Allowed	Outliers	Percentiles	
1	A	521/538 (97%)	489 (94%)	26 (5%)	6 (1%)	13	4

All (6) Ramachandran outliers are listed below:

Mol	Chain	Res	Type
1	A	115	VAL
1	A	148	LYS
1	A	149	VAL
1	A	105	ILE
1	A	153	SER
1	A	150	LYS

5.3.2 Protein sidechains (i)

In the following table, the Percentiles column shows the percent sidechain outliers of the chain as a percentile score with respect to all X-ray entries followed by that with respect to entries of similar resolution.

The Analysed column shows the number of residues for which the sidechain conformation was analysed, and the total number of residues.

Mol	Chain	Analysed	Rotameric	Outliers	Percentiles	
1	A	437/461 (95%)	435 (100%)	2 (0%)	88	89

All (2) residues with a non-rotameric sidechain are listed below:

Mol	Chain	Res	Type
1	A	25	THR
1	A	126	GLU

CONFIDENTIAL VALIDATION REPORT

Sometimes sidechains can be flipped to improve hydrogen bonding and reduce clashes. All (2) such sidechains are listed below:

Mol	Chain	Res	Type
1	A	106	ASN
1	A	367	ASN

5.3.3 RNA (i)

There are no RNA molecules in this entry.

5.4 Non-standard residues in protein, DNA, RNA chains (i)

There are no non-standard protein/DNA/RNA residues in this entry.

5.5 Carbohydrates (i)

2 monosaccharides are modelled in this entry.

In the following table, the Counts columns list the number of bonds (or angles) for which Mogul statistics could be retrieved, the number of bonds (or angles) that are observed in the model and the number of bonds (or angles) that are defined in the Chemical Component Dictionary. The Link column lists molecule types, if any, to which the group is linked. The Z score for a bond length (or angle) is the number of standard deviations the observed value is removed from the expected value. A bond length (or angle) with $|Z| > 2$ is considered an outlier worth inspection. RMSZ is the root-mean-square of all Z scores of the bond lengths (or angles).

Mol	Type	Chain	Res	Link	Bond lengths			Bond angles		
					Counts	RMSZ	$\#\|Z\| > 2$	Counts	RMSZ	$\#\|Z\| > 2$
2	NAG	G	1	2	15,15,15	0.63	0	21,21,21	1.33	3 (14%)
2	NAG	G	2	2	14,14,15	0.37	0	17,19,21	0.89	1 (5%)

In the following table, the Chirals column lists the number of chiral outliers, the number of chiral centers analysed, the number of these observed in the model and the number defined in the Chemical Component Dictionary. Similar counts are reported in the Torsion and Rings columns. '-' means no outliers of that kind were identified.

Mol	Type	Chain	Res	Link	Chirals	Torsions	Rings
2	NAG	G	1	2	-	0/6/26/26	0/1/1/1
2	NAG	G	2	2	-	0/6/23/26	0/1/1/1

There are no bond length outliers.

All (4) bond angle outliers are listed below:

CONFIDENTIAL VALIDATION REPORT

Mol	Chain	Res	Type	Atoms	Z	Observed(°)	Ideal(°)
2	G	1	NAG	C1-C2-C3	-3.62	105.61	110.54
2	G	1	NAG	O5-C1-C2	-2.48	107.03	109.52
2	G	1	NAG	C1-O5-C5	-2.38	109.18	113.66
2	G	2	NAG	O5-C5-C6	2.32	110.84	107.20

There are no chirality outliers.

There are no torsion outliers.

There are no ring outliers.

No monomer is involved in short contacts.

5.6 Ligand geometry (i)

Of 4 ligands modelled in this entry, 3 are monoatomic - leaving 1 for Mogul analysis.

In the following table, the Counts columns list the number of bonds (or angles) for which Mogul statistics could be retrieved, the number of bonds (or angles) that are observed in the model and the number of bonds (or angles) that are defined in the Chemical Component Dictionary. The Link column lists molecule types, if any, to which the group is linked. The Z score for a bond length (or angle) is the number of standard deviations the observed value is removed from the expected value. A bond length (or angle) with $|Z| > 2$ is considered an outlier worth inspection. RMSZ is the root-mean-square of all Z scores of the bond lengths (or angles).

Mol	Type	Chain	Res	Link	Bond lengths			Bond angles		
					Counts	RMSZ	$\#\|Z\| > 2$	Counts	RMSZ	$\#\|Z\| > 2$
3	EDO	A	601	-	3,3,3	0.11	0	2,2,2	0.35	0

In the following table, the Chirals column lists the number of chiral outliers, the number of chiral centers analysed, the number of these observed in the model and the number defined in the Chemical Component Dictionary. Similar counts are reported in the Torsion and Rings columns. '-' means no outliers of that kind were identified.

Mol	Type	Chain	Res	Link	Chirals	Torsions	Rings
3	EDO	A	601	-	-	0/1/1/1	-

There are no bond length outliers.

There are no bond angle outliers.

There are no chirality outliers.

There are no torsion outliers.

There are no ring outliers.

No monomer is involved in short contacts.

CONFIDENTIAL VALIDATION REPORT

5.7 Other polymers (i)

There are no such residues in this entry.

5.8 Polymer linkage issues (i)

There are no chain breaks in this entry.

CONFIDENTIAL VALIDATION REPORT

6 Fit of model and data (i)

6.1 Protein, DNA and RNA chains (i)

In the following table, the column labelled '#RSRZ> 2' contains the number (and percentage) of RSRZ outliers, followed by percent RSRZ outliers for the chain as percentile scores relative to all X-ray entries and entries of similar resolution. The OWAB column contains the minimum, median, 95th percentile and maximum values of the occupancy-weighted average B-factor per residue. The column labelled 'Q< 0.9' lists the number of (and percentage) of residues with an average occupancy less than 0.9.

Mol	Chain	Analysed	<RSRZ>	#RSRZ>2	OWAB(Å^2)	Q<0.9
1	A	524/538 (97%)	0.30	29 (5%) 25 28	19, 31, 61, 72	0

All (29) RSRZ outliers are listed below:

Mol	Chain	Res	Type	RSRZ
1	A	145	VAL	4.2
1	A	431	GLY	4.0
1	A	154	THR	4.0
1	A	93	LEU	4.0
1	A	97	LYS	3.4
1	A	73	GLY	3.4
1	A	149	VAL	3.3
1	A	84	ALA	3.3
1	A	123	ARG	2.9
1	A	147	SER	2.9
1	A	152	PRO	2.8
1	A	432	ASP	2.8
1	A	61	ASP	2.7
1	A	117	ASP	2.7
1	A	480	THR	2.7
1	A	110	THR	2.5
1	A	64	MET	2.5
1	A	115	VAL	2.5
1	A	161	VAL	2.5
1	A	479	VAL	2.4
1	A	481	ILE	2.4
1	A	79	GLY	2.3
1	A	33	PHE	2.3
1	A	105	ILE	2.2
1	A	74	VAL	2.2
1	A	81	ALA	2.2
1	A	121	LYS	2.2

Continued on next page...

CONFIDENTIAL VALIDATION REPORT

Continued from previous page...

Mol	Chain	Res	Type	RSRZ
1	A	127	ALA	2.2
1	A	458	ALA	2.2

6.2 Non-standard residues in protein, DNA, RNA chains (i)

There are no non-standard protein/DNA/RNA residues in this entry.

6.3 Carbohydrates (i)

In the following table, the Atoms column lists the number of modelled atoms in the group and the number defined in the chemical component dictionary. The B-factors column lists the minimum, median, 95th percentile and maximum values of B factors of atoms in the group. The column labelled 'Q< 0.9' lists the number of atoms with occupancy less than 0.9.

Mol	Type	Chain	Res	Atoms	RSCC	RSR	B-factors(Å^2)	Q<0.9
2	NAG	G	1	15/15	0.91	0.12	18,24,32,38	0
2	NAG	G	2	14/15	0.95	0.11	19,23,28,30	0

6.4 Ligands (i)

In the following table, the Atoms column lists the number of modelled atoms in the group and the number defined in the chemical component dictionary. The B-factors column lists the minimum, median, 95th percentile and maximum values of B factors of atoms in the group. The column labelled 'Q< 0.9' lists the number of atoms with occupancy less than 0.9.

Mol	Type	Chain	Res	Atoms	RSCC	RSR	B-factors(Å^2)	Q<0.9
6	MG	A	604	1/1	0.96	0.12	25,25,25,25	0
3	EDO	A	601	4/4	0.96	0.10	24,25,26,33	0
5	CL	A	603	1/1	0.98	0.03	24,24,24,24	0
4	CA	A	602	1/1	0.99	0.03	25,25,25,25	0

6.5 Other polymers (i)

There are no such residues in this entry.

CONFIDENTIAL VALIDATION REPORT
